# Pyridylpiperazine-based carbodithioates as urease inhibitors: synthesis and biological evaluation

**DOI:** 10.3389/fchem.2024.1423385

**Published:** 2024-08-06

**Authors:** Muhammad Akash, Nehal Rana, Sana Aslam, Matloob Ahmad, Muhammad Jawwad Saif, Aneeza Asghar, Sadia Sultan, Sami A. Al-Hussain, Afifa Liaqat, Sumera Zaib, Magdi E. A. Zaki

**Affiliations:** ^1^ Department of Chemistry, Government College University Faisalabad, Faisalabad, Pakistan; ^2^ Department of Basic and Applied Chemistry, Faculty of Science and Technology, University of Central Punjab, Lahore, Pakistan; ^3^ Department of Chemistry, Government College Women University Faisalabad, Faisalabad, Pakistan; ^4^ Department of Applied Chemistry, Government College University Faisalabad, Faisalabad, Pakistan; ^5^ Faculty of Pharmacy, Universiti Teknologi MARA, Puncak Alam, Malaysia; ^6^ Atta-ur-Rahman Institute for Natural Products Discovery (AuRIns), Universiti Teknologi MARA, Puncak Alam, Malaysia; ^7^ Department of Chemistry, College of Science, Imam Mohammad Ibn Saud Islamic University (IMSIU), Riyadh, Saudi Arabia

**Keywords:** pyridine and piperazine derivatives, heterocyclic carbodithioates, urease inhibitors, molecular docking, synthesis and biological evaluation

## Abstract

The urease enzyme is recognized as a valuable therapeutic agent for treating the virulent *Helicobacter pylori* bacterium because of its pivotal role in aiding the colonization and growth of the bacterium within the gastric mucosa. In order to control the harmful consequences of bacterial infections, urease inhibition presents itself as a promising and effective approach. The current research aimed to synthesize pyridylpiperazine-based carbodithioate derivatives **5a–5n** and **7a–7n** that could serve as potential drug candidates for preventing bacterial infections through urease inhibition. The synthesized carbodithioate derivatives **5a–5n** and **7a–7n** were explored to assess their ability to inhibit the urease enzyme after their structural explication by gas chromatography–mass spectrometry (GC-MS). In the *in vitro* evaluation with thiourea as a standard drug, it was observed that all the synthesized compounds exhibited significant inhibitory activity compared to the reference drug. Among the compounds tested, **5j** (bearing an o-tolyl moiety) emerged as the most effective inhibitor, displaying strong urease inhibition with an IC_50_ value of 5.16 ± 2.68 μM. This IC_50_ value is notably lower than that of thiourea (23 ± 0.03 μM), indicating the significantly most potent potential of inhibition. In molecular docking of **5j** within the active site of urease, numerous noteworthy interactions were identified.

## 1 Introduction

Urease is an enzyme with two Ni^2+^ ions at its active site, which facilitates the breakdown of urea into carbonic acid and ammonia by the formation of carbamic acid. Within living organisms, this is the ultimate stage of nitrogen metabolism. It can be found in a diverse range of organisms, including algae, plants, and fungi (Svane et al., 2020). The principal physiological function of urease is to furnish organisms with nitrogen in the form of ammonia to support their growth. Despite that, excessive urease activity can result in the liberation of unusually high levels of ammonia into the atmosphere, potentially causing environmental and economic challenges (Hanif et al., 2012). Urease plays a significant role in the pathogenesis of diseases caused by *Helicobacter pylori* (Mahernia et al., 2015). *H. pylori* is a Gram-negative, microaerophilic bacterium that has a significant global presence, affecting more than half of the world population. It typically establishes itself during childhood and, if not addressed, can potentially endure throughout an individual’s lifetime (Tempera et al., 2022).

Urease is a key enzyme that benefits the *H. pylori* bacterium by enabling its survival in the acidic conditions of the stomach. Consequently, this bacterial presence can lead to gastrointestinal (GIT) diseases, peptic ulcer, gastritis, and even gastric cancer (Mahernia et al., 2015). Urease activity is a critical factor in *H. pylori* infection, as bacteria lacking functional urease lose their capacity to establish an infection and colonize the host (Gull et al., 2016). Over the past few decades, various treatment regimens have been proposed for curing *H. pylori* infection. Among these, the triple-therapy regimens comprising proton pump, amoxicillin, and clarithromycin inhibitors have been the most commonly prescribed method for *H. pylori* eradication. Despite achieving a success rate of 80%, it is imperative to acknowledge certain drawbacks, including unwanted side effects and the emergence of antibiotic resistance, all of which somewhat limit its clinical utility (Zhou et al., 2017). However, in the early stages of the infection, targeting the activity of urease can eliminate the bacterium. The research interest in designing novel urease inhibitors has surged due to the role of urease in bacterial infections (Imran et al., 2020). Therefore, the primary approach for managing infections caused by microorganisms that produce urease involves the use of urease inhibitors (Alqahtani et al., 2022).

In recent decades, researchers have successfully crystallized urease enzymes derived from various bacterial and plant sources. These crystals were obtained both in their isolated form and in conjunction with inhibitors. As a result, we now possess a comprehensive understanding of both the functions and structural characteristics of urease enzymes at the molecular level (Hameed et al., 2019). The primary approach has extensively focused on identifying urease inhibitors that either directly bind to the di-nickeled ions located in the active site enzyme or disrupt its catalytic cycle. Despite the existence of a considerable number of known urease inhibitors, this approach has yielded limited success to date. Only a small subset of these compounds has been evaluated in therapeutic studies, and they have raised concerns related to their effectiveness and safety when used *in vivo* (Tarsia et al., 2019).

**FIGURE 1:**
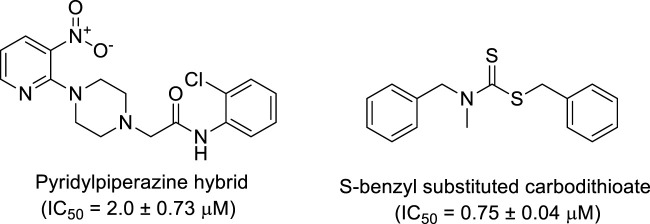
Chemical structures of the pyridylpiperazine hybrid and *S*-benzyl-substituted carbodithioate—highly potent urease inhibitors.

Additionally, some of these compounds have proved ineffective in therapy due to either their low stability, limited bioavailability, or the need for exceptionally high doses. Furthermore, these inhibitors often struggle to efficiently penetrate the plasma membrane of Gram-negative bacteria to access their target within the cytoplasm (Zambelli et al., 2014). To date, there exists only a single clinically approved effective inhibitor, acetohydroxamic acid, although it causes associated adverse side effects. Enzymes have emerged as crucial targets for drug development, and enzyme inhibitors have shown significant success as drugs (Yang et al., 2018). A prevalent approach in designing drugs that target enzymes involves the identification or development of structural analogs resembling the enzyme substrates, effectively mimicking their reactivity. Nevertheless, this approach can face limitations when enzyme active sites are not readily accessible to solvents or when enzyme substrates exhibit a high degree of specificity (Rutherford, 2014). In our recent studies, the pyridylpiperazine hybrid was found to be a highly active scaffold against the urease enzyme (Figure 1) (Akash et al., 2024). In another report, *S*-benzyl-substituted carbodithioate emerged as a highly potent urease inhibitor (Figure 1) (Khan et al., 2024). The coupling of two or more pharmacologically important scaffolds is a diverse approach that can produce highly potent hybrid molecules. In continuation of our previous research dealing with the development of urease enzyme inhibitors (Akash et al., 2024), we herein report a novel series of pyridylpiperazine-based carbodithioates as potent urease inhibitors.

## 2 Results and discussions

### 2.1 Chemistry

**SCHEME 1:**
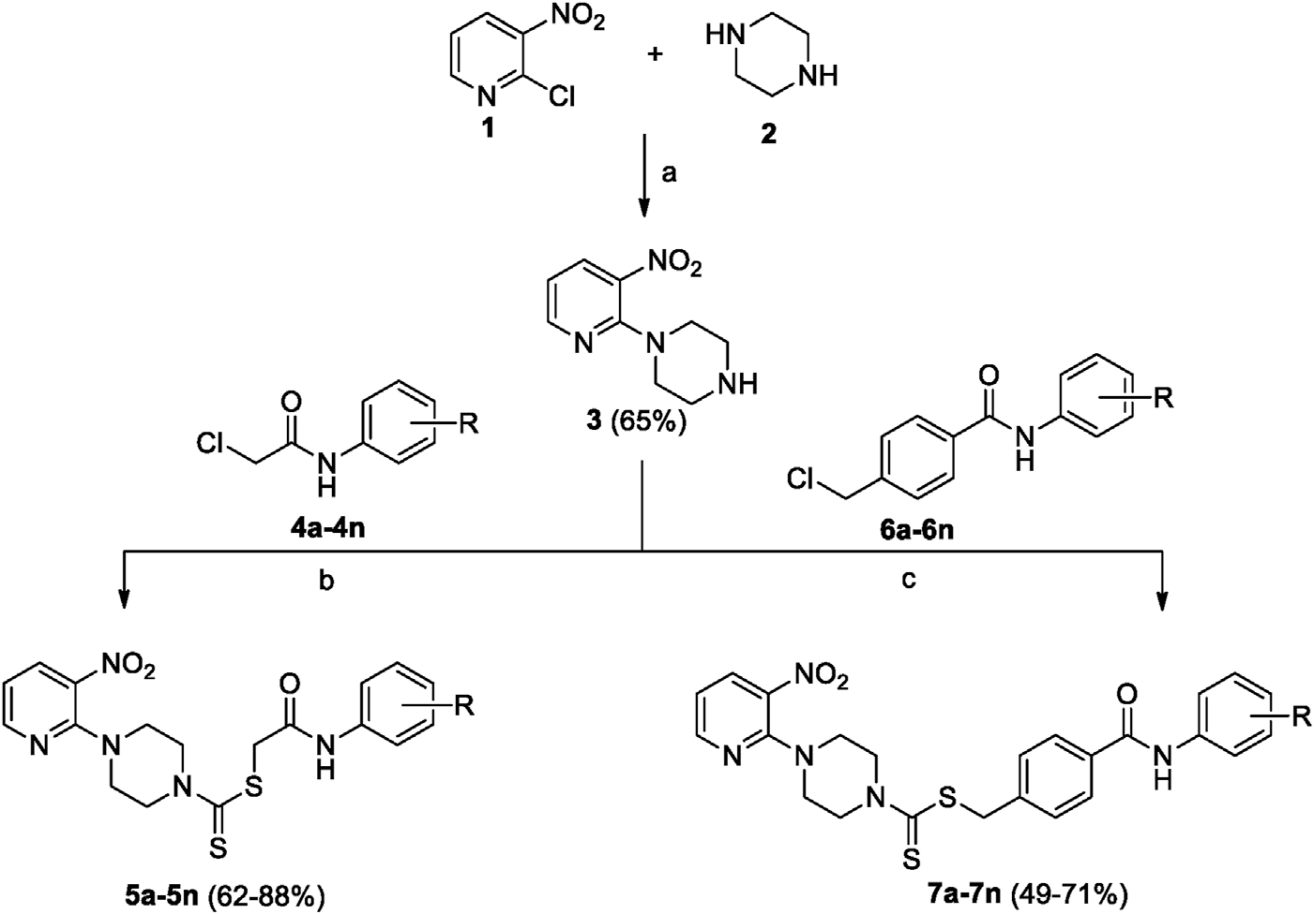
2-Oxo-2-(arylamino)ethyl 4-(3-nitropyridin-2-yl)piperazine-1-carbodithioates **5a**–**5n** and 4-((aryl)carbamoyl)benzyl 4-(3-nitropyridin-2-yl)piperazine-1-carbodithioates **7a**–**7n**. Reagents and conditions: (a) acetonitrile, reflux, 12 h; (b) CS_2_, NaOAc acetonitrile, reflux, 8–16 h; and (c) CS_2_, NaOAc, acetonitrile, reflux, 12–24 h.

**FIGURE 2:**
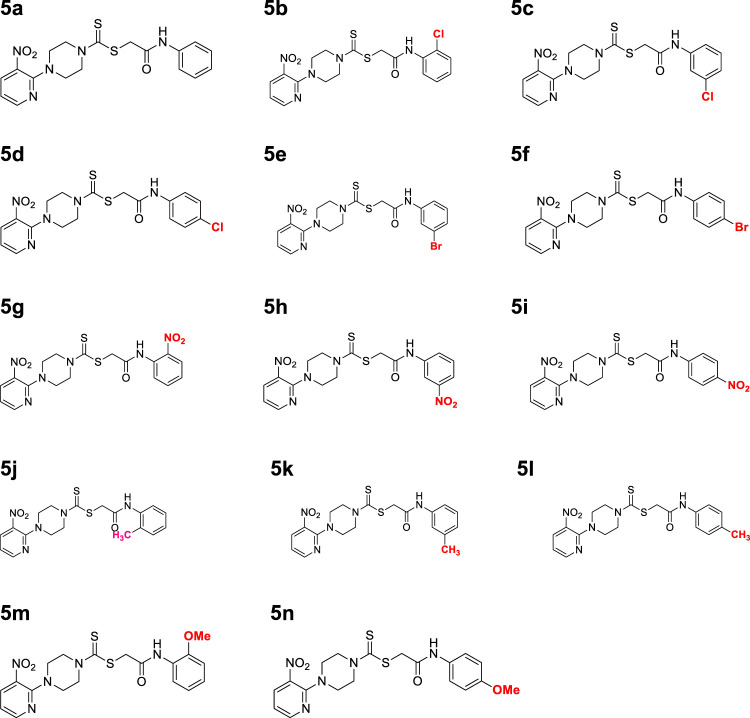
Chemical structures of compounds **5a**–**5n**.

**FIGURE 3:**
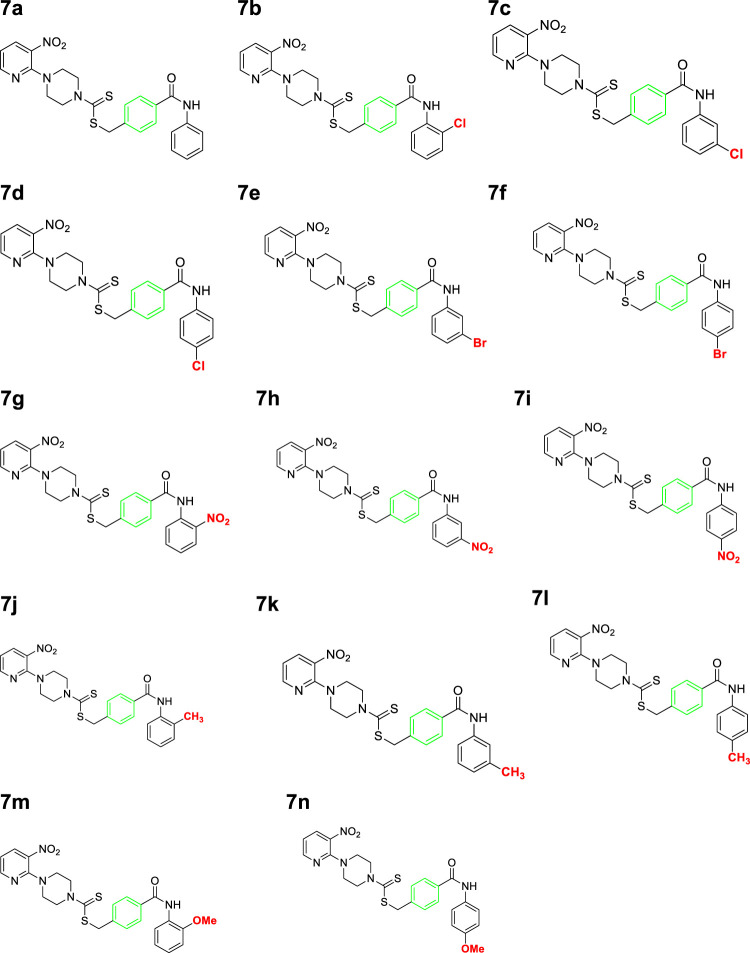
Chemical structures of compounds **7a**–**7n**.

A number of 2-oxo-2-(arylamino)ethyl 4-(3-nitropyridin-2-yl)piperazine-1-carbodithioates **5a**–**5n** and 4-((aryl)carbamoyl)benzyl 4-(3-nitropyridin-2-yl)piperazine-1-carbodithioates **7a**–**7n** were synthesized, as shown in Scheme 1. The treatment of 2-chloro-3-nitropyridine **1** carried out with excess of piperazine **2** in acetonitrile on reflux for 12 h furnished 1-(3-nitropyridin-2-yl)piperazine **3** in 65% yield. The reaction of pyridinylpiperazine **3** with 2-chloro-*N*-arylacetamides **4a**–**4n** in the presence of CS_2_ and NaOAc in methanol under reflux for 8–16 h produced 2-oxo-2-(arylamino)ethyl 4-(3-nitropyridin-2-yl)piperazine-1-carbodithioate **5a**–**5n** (Figure 2) in average in good yield (62%–88%). In a similar manner, the reaction between pyridinylpiperazine **3** and 4-(chloromethyl)-N-arylbenzamide **6a–6n** in the presence of CS_2_ and NaOAc in methanol under reflux for 12–24 h produced 4-((aryl)carbamoyl)benzyl 4-(3-nitropyridin-2-yl)piperazine-1-carbodithioate **7a**–**7n** (Figure 3) in moderate yield (49%–71%). All the synthesized compounds **5a**–**5n** and **7a**–**7n** were extracted from the reaction mixture via precipitation and purified by column chromatography. The structures were elucidated via spectroscopic techniques. The HRMS proved the predicted chemical formula the molecular ion peak of each compound. The ^1^H NMR spectrum of **5a**–**5n** and **7a**–**7n** justifies the presence of methylene protons at a shift value (δ) of 4.25–4.35 ppm and 4.66–4.69 ppm, respectively. Meanwhile, the ^13^C NMR spectra of **5a**–**5n** and **7a**–**7n** revealed the presence of S-C=S in all the compounds at a shift value (δ) of 194.4–195.3 ppm.

### 2.2 *In vitro* inhibition and structure–activity relationship analysis

Using thiourea as a standard (IC_50_ = 23.00 ± 0.03 μM), the compounds that we synthesized were estimated against urease for their inhibitory potential. All the experimental data are given in Table 1. Generally, the compounds **(5a, 5f, 5g, 7e, 7f, 5j**–**5n, 7a, 7c, 7e, 7f, 7i**–**7k**, and **7m)** showed potent activity against the urease enzyme in a range of 5.16–21.34 μM. The *in vitro* analysis showed that compound **5j** was the most effective inhibitor of urease in the series, possessing an IC_50_ value of 5.16 ± 2.68 μM, which is 22-fold more potent than the standard (thiourea; IC_50_ = 23.00 ± 0.03 μM). The compounds **5j, 5k,** and **5l** possess a methyl group at the *ortho, meta*, and *para* positions, respectively; however, **5j**, with an IC_50_ value of 5.16 ± 2.68 μM, revealed stronger inhibitory activity against urease than **5k** (IC_50_ = 18.30 ± 0.17 μM) and **5l** (IC_50_ = 10.61 ± 0.36 μM). A notable decrease was observed in the activity of compounds **5b** (IC_50_ = 41.03 ± 0.23 μM), **5c** (IC_50_ = 37.33 ± 0.19 μM), and **5d** (IC_50_ = 55.69 ± 0.20 μM) when the chloro (-Cl) group was added instead of the methyl (-CH_3_) group in piperazine-1-carbodithioate. The presence of the nitro (-NO_2_) group in compound **5g** with IC_50_ = 16.84 ± 0.12 μM as a substituent at the ortho position exhibited effective inhibitory potential, while the nitro group at the meta and para positions in compounds **5h** with IC_50_ = 26.98 ± 2.15 μM and **5i** with IC_50_ = 45.41 ± 0.23 μM showed poor inhibitory potential compared to the positive control (thiourea). Furthermore, the compounds **5n** (17.77 ± 0.29 μM) with the methoxy group at position 4 and **5a** (IC_50_ = 18.67 ± 0.52 μM) with no substituent revealed almost the same inhibitory activity, while compound **5m** with the methoxy group at position 2 showed effective inhibitory activity with an IC_50_ value of 08.17 ± 0.37 μM. Effective inhibition was shown by **7j** (IC_50_ = 12.26 ± 0.27 μM) and **7k** (IC_50_ = 10.65 ± 0.26 μM) when the tolylcarbamoyl group was attached at the ortho and meta positions, respectively; however, a decrease in activity was observed when the same substituent was attached at the para position in compound **7l** with IC_50_ = 37.54 ± 0.26 μM. When methoxyphenyl was inserted at position 2 on **7m** (IC_50_ = 18.67 ± 0.27 μM), it showed good inhibition; however, when the same group was introduced at position 4 in **7n** (IC_50_ = 24.02 ± 0.27 μM), the inhibition activity was not effective. The addition of the nitrophenyl group at position 2 and position 3 in **7g** (IC_50_ = 56.89 ± 0.31 μM) and **7h** (IC_50_ = 47.94 ± 0.25 μM) showed poor inhibition, while when the nitrophenyl group was introduced at position 4 instead of position 2 or 3 in compound **7i** with IC_50_ of 10.51 ± 0.34 μM, it showed effective inhibitory activity against urease. Compound **7a** without any substitution exhibited IC_50_ of 11.69 ± 0.26 μM and revealed effective inhibitory activity. However, when the chlorophenyl group was attached at position 3 in **7c** (IC_50_ = 10.80 ± 0.52 μM), the same inhibitory potential was observed, while when the same group was added at positions 2 and 4 in compounds **7b** (IC_50_ = 32.53 ± 0.31 μM) and **7d** (IC_50_ = 43.11 ± 0.31 μM), respectively, the inhibitory activity decreased. The same inhibitory potential against urease was shown by **7e** (IC_50_ = 16.50 ± 0.28 μM) and **7f** (IC_50_ = 16.83 ± 0.29 μM) when the bromophenyl group was inserted at position 3 in **7e** and at position 4 in **7f**.

**TABLE 1:** Inhibitory concentration of the synthesized compounds and thiourea against urease was determined by calculating the IC_50_ values.

Compound	R	Urease inhibition: IC_50_ ± SEM (μM)
**5a**	H	18.67 ± 0.52
**5b**	2-Cl	41.03 ± 0.23
**5c**	3-Cl	37.33 ± 0.19
**5d**	4-Cl	55.69 ± 0.20
**5e**	3-Br	32.53 ± 0.20
**5f**	4-Br	21.34 ± 0.7
**5g**	2-NO_2_	16.84 ± 0.12
**5h**	3-NO_2_	26.98 ± 2.15
**5i**	4-NO_2_	45.41 ± 0.23
**5j**	2-Me	5.16 ± 2.68
**5k**	3-Me	18.30 ± 0.17
**5l**	4-Me	10.61 ± 0.36
**5m**	2-OMe	8.17 ± 0.37
**5n**	4-OMe	17.77 ± 0.29
**7a**	H	11.69 ± 0.26
**7b**	2-Cl	32.53 ± 0.31
**7c**	3-Cl	10.80 ± 0.52
**7d**	4-Cl	43.11 ± 0.31
**7e**	3-Br	16.50 ± 0.28
**7f**	4-Br	16.83 ± 0.29
**7g**	2-NO_2_	56.89 ± 0.31
**7h**	3-NO_2_	47.94 ± 0.25
**7i**	4-NO_2_	10.51 ± 0.34
**7j**	2-Me	12.26 ± 0.27
**7k**	3-Me	10.65 ± 0.26
**7l**	4-Me	37.54 ± 0.26
**7m**	2-OMe	18.67 ± 0.27
**7n**	4-OMe	24.02 ± 0.27
**Thiourea** (standard)		22.3 ± 0.03

Bold values represents the Compound numbers.

Comparing both the series and the impact of substituents on the activity of the overall nucleus showed that halogen substitutions are favorable for the **7** series compared to the **5** series. Among halogen substituents, chlorine substitution at the meta position of **7c** was more impactful than that in **5c**. Similarly, the presence of bromine at either the meta or para position of **7e** and **7f** showed similar and good inhibitory results compared to that in **5e** and **5f**.

Nitro-group substitutions at the ortho and meta positions were found to be favorable for increasing the urease inhibitory activities of **5g** and **5h**, while at the para position, the nitro group enhances the inhibitory potential of **7i**. Methyl substitution at the ortho position was favorable for **5j**, while when placed at the meta position, it increased the inhibitory potential of **7k** but not of **5k**. Contrarily, the methyl group at the para position increases the urease inhibition for **5l**. Lastly, methoxy substitution showed better outcomes in **5m** and **5n** than in **7m** (Table 1).

### 2.3 Kinetic studies

**FIGURE 4:**
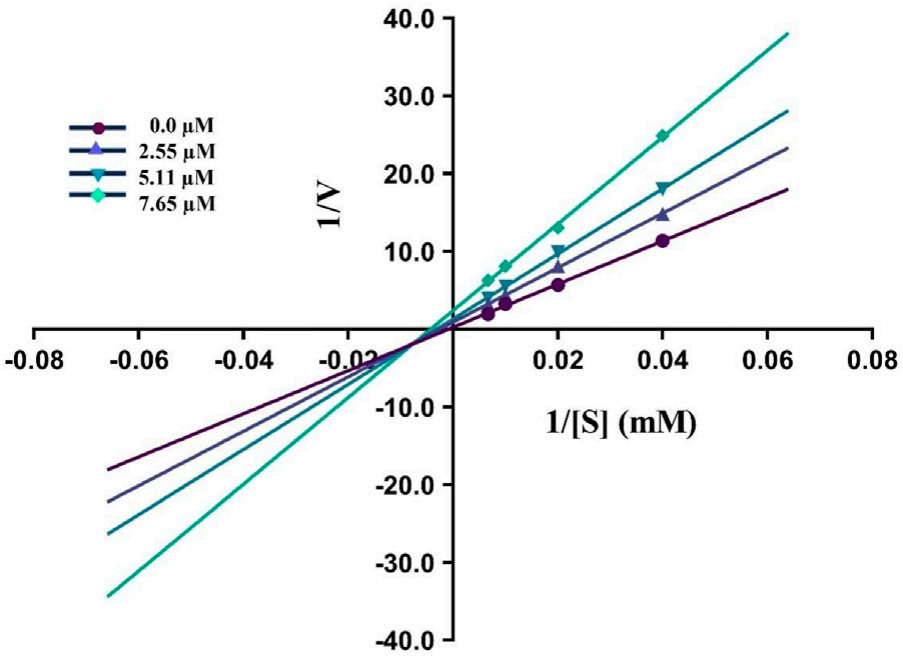
Demonstrated the Inhibition type of compound **5j** against urease.

By using Lineweaver–Burk graphs, we determined the mechanism of action of the leading compound **5j** against urease. We evaluated the effect of the inhibitor on K_*m*_ and V_*max*_ using reciprocal 1/S and 1/V of the product concentration and calculated the inhibition type. The slope K_*m*_/V_*max*_ of each line was schemed against different concentrations of the substrate and inhibitor. In kinetic studies of the potent compound, different concentrations of the compound and substrate were used. Different concentrations of 1 mM **5j** were 0, 2.55, 5.11, and 7.65 μM, and different concentrations of the substrate were 0, 25, 50, 100, and 150 mM. Compound **5j** is shown in Figure 4, which illustrates a mixed type of inhibition against urease.

### 2.4 Molecular docking and intermolecular interactions

**FIGURE 5:**
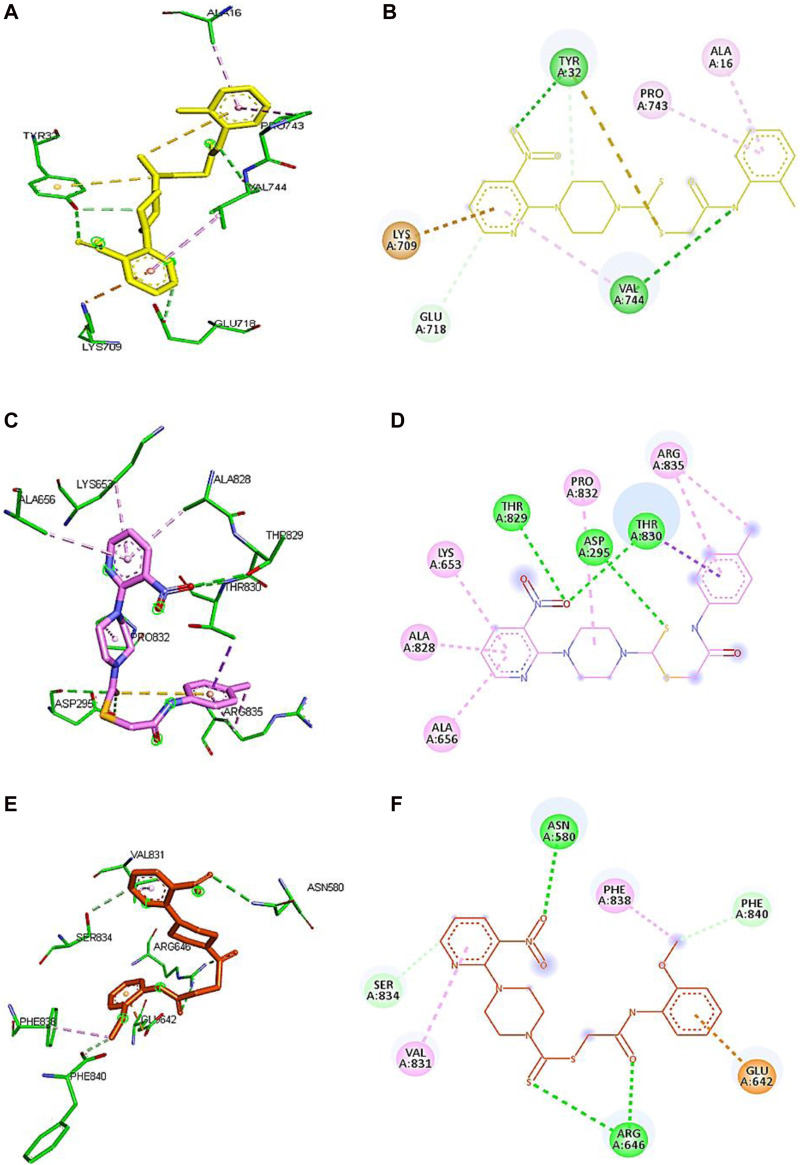
Demonstration of 3D and 2D interactions of compounds **5j**, **5m**, and **5l** with urease. Green represents the amino acid residues, while yellow, purple, and red show the ligand in 3D structure **(A,C,E)**. It explains the correlation between the ligand and residues of the amino acid by different types of interactions; **(B,D,F)** 2D image of the ligand and amino acid residues.

Based on the results of dilutions, the five compounds **5j, 5l, 5m**, **7i**, and **7k** were selected to perform molecular docking against urease (PDB ID: 3LA4). Compounds **5j, 5l, 5m, 7i**, and **7k** exhibited binding affinities of −7.1, −6.6, −6.6, −7.4, and −6.7 kcal/mol, respectively, with the first pose. On the other hand, the binding energy of thiourea was found to be −3.2 kcal/mol when docked against the same binding site of urease. However, for further visualization through Discovery Studio, we selected the first pose of all the compounds and thiourea with the lowest binding affinity. The 2D and 3D interactions for compounds **5j, 5l, 5m, 7i**, and **7k** against urease are shown in Figure 5. Compounds **5j**, **5l**, **and 5m** revealed conventional hydrogen, alkyl, C-H, and π–alkyl bonds with different residues of amino acids, as shown in Table 2.

**TABLE 2:** Binding interactions and type of compounds against urease. Different interactions between amino acid residues and ligand are reported below.

Binding interactions				
Compounds	Ligand atoms	Receptor residues	Interaction type	Distance (Å)
**5j**	Oxygen, piperazine ring	Tyr32	H-bond and π–sulfur	3.21, 5.98
	Nitrogen	Val744	H-bond	2.92
	Aromatic ring	Pro743	π–alkyl	5.1
	Aromatic ring	Ala16	π–alkyl	4.57
	Pyridine ring	Lys709	π–cation	4.08
	Pyridine ring	Glu718	C–H bond	3.59
**5l**	Pyridine ring	Ala656	π–alkyl	4.20
	Pyridine ring	Ala828	π–alkyl	4.33
	Pyridine ring	Lys653	π–alkyl	5.36
	Piperazine ring	Pro832	alkyl	4.88
	Aromatic ring	Arg835	π–alkyl	5.27
	Oxygen	Thr829	H-bond	3.60
	Sulfur	Asp295	H-bond	5.27
	Oxygen	Thr830	H-bond	3.13
**5m**	Pyridine ring	Val831	π–alkyl	5.36
	Pyridine ring	Ser834	C–H bond	3.68
	Oxygen	Asn580	H-bond	3.06
	Methoxy group	Phe838	π–alkyl	4.34
	Sulfur	Arg646	H-bond	3.79
	Methoxy group	Phe840	π–alkyl	4.34
	Aromatic ring	Glu642	π–anion	3.46
**7k**	O35	Arg639	H bond	3.96
	C12	Glu642	C–H bond	3.75
	Sulfur	Phe838	π–sulfur	5.43
	Aromatic ring	Glu418	π–anion	4.24
	N22	Met746	H-bond	3.03
	C32	Tyr417	π–alkyl	5.29
	Aromatic ring	Trp728	π–π T-shaped	5.19
**7i**	O35	Arg639	H-bond	3.96
	C12	Glu642	C–H bond	3.75
	Sulfur	Phe838	π–sulfur	5.43
	Aromatic ring	Glu418	π–anion	4.24
	O24	Met746	H-bond	3,14
	C32	Tyr417	π–anion	5.29
	Aromatic ring	Trp728	π–π T-shaped	5.19
**Thiourea**	H6	Ser421	H-bond	2.09
	H5	Thr715	H-bond	2.04
	H7	Thr715	H-bond	2.61

Bold values represents the Compound numbers.

**FIGURE 6:**
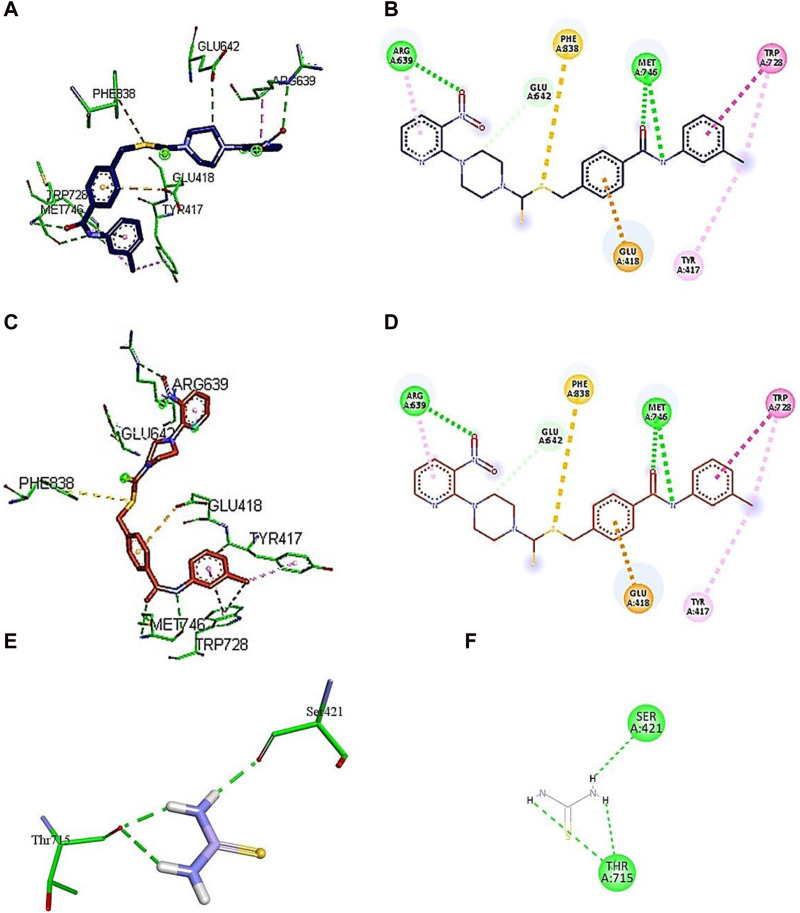
Demonstration of 3D and 2D interactions of compounds **7k** and **7i** and thiourea with urease. Green represents the amino acid residues, while blue, red, and purple show the ligand in 3D structure **(A, C,E)**. It explains the correlation between the ligand and residues of the amino acid by different types of interactions; **(B,D,F)** 2D image of the ligand and amino acid residues.

Compound **5j** interacts with the urease binding site by hydrophilic, hydrophobic, and electrostatic interactions. The pyridine ring of **5j** forms π–cation and carbon–hydrogen bonds with Lys709 (4.08 Å) and Glu718 (3.59 Å) of the urease active site, respectively. The aromatic ring of **5j** exhibits π–alkyl interactions with Pro743 and Ala16 of the active pocket. Lastly, hydrogen bond interactions are also present between Tyr32 (3.21 Å) and oxygen and nitrogen and Val744 (2.92 Å). Similarly, the pyridine ring of **5l** forms π–alkyl interactions with Ala656 (4.20 Å), Ala828 (4.33 Å), and Lys653 (5.36 Å). Apart from the pyridine ring, the aromatic ring of **5l** also develops π–alkyl interactions with Arg835 (5.27 Å) of urease. Moreover, Thr829 (3.60 Å), Asp295 (5.27 Å), and Thr830 (3.13 Å) of urease are found to be involved in hydrogen bond interactions with the oxygen and sulfur atoms of **5l**, as shown in Figure 6. In the case of **5m**, the pyridine ring is involved in the π–alkyl and hydrogen bond interaction formation with Val831 (5.36 Å) and Ser834 (3.68 Å), respectively. Other hydrogen bond interactions are formed by Asn580 (3.06 Å) and Arg646 (3.79 Å) of urease with the oxygen and sulfur of **5m**, respectively. Furthermore, the substituent of **5m**, the methoxy group, also forms π–alkyl interactions with Phe838 (4.34 Å) and Phe840 (4.34 Å) of the active site of urease.

A conventional hydrogen bond was revealed between MET746 oxygen and nitrogen of compound **7k** with a distance of 3.14 and 3.03 Å, respectively, and the same type of interaction was found between ARG639 and the oxygen atom, with a distance of 3.97 Å. The carbon–hydrogen bond was observed between GLU642 and the benzene ring of the compound (3.75 Å). The π–anion interactions were observed between the sulfur atom and PHE838 (5.43 Å). The π–sulfur interactions were shown between the benzene ring of the compound and GLU418 with a distance of 4.24 Å. The π–π T-shaped interactions were revealed between the benzene ring and TRP728 (5.09 Å). Moreover, the π–alkyl interactions were observed between TRP728 (5.19 Å) and the carbon atom, TYR417 (5.29 Å) and the carbon atom, and ARG639 (3.96 Å) and the benzene ring of the compound. A conventional hydrogen bond was observed between MET746 oxygen and nitrogen of compound **7i** with a distance of 3.14 and 3.03 Å, respectively, and the same type of interaction was found between ARG639 and the oxygen atom with a distance of 3.97 Å. The carbon–hydrogen bond was observed between GLU642 and the benzene ring of the compound (3.75 Å). The π–anion bond was observed between the sulfur atom and PHE838 (5.43 Å). The π–sulfur interactions were observed between the benzene ring of the compound and GLU418 with a distance of 4.24 Å. The π–π T-shaped interactions were revealed the between benzene ring and TRP728 (5.09 Å). Moreover, the π–alkyl interactions were observed between TRP728 (5.19 Å) and the carbon atom, TYR417 (5.29 Å) and the carbon atom, and ARG639 (3.96 Å) and the benzene ring of the compound, as shown in Figure 6. The interactions of thiourea were also analyzed, which showed that it only develops conventional hydrogen bond interactions with Ser421 (2.09 Å) and Thr715 (2.04 and 2.61 Å).

In light of the above discussion and literature review, it has been observed that interactions with Ala16, Tyr32, Asp235, Val744, Thr830, Val831, Pro832, Ser834, Arg835, and Phe840 residues in the urease binding site are crucial. It is because these are involved in the inhibition of urease by interacting with numerous active compounds (Khan et al., 2019; Hina et al., 2023; Akash et al., 2024).

### 2.5 Molecular dynamics simulation

**FIGURE 7:**
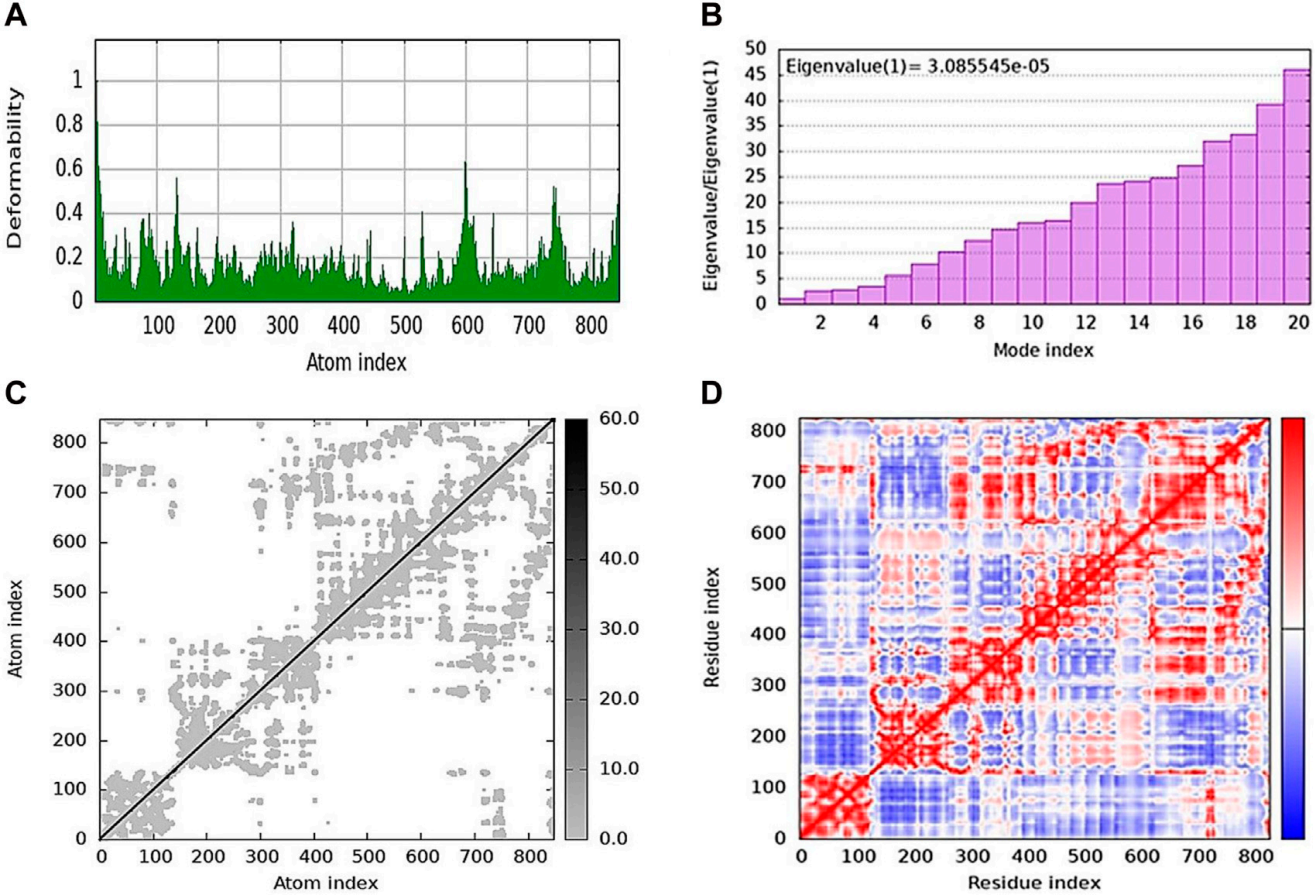
Molecular dynamics simulation of **5j** obtained from the iMOD server. **(A)** Deformability plot indicating that most residues are stable. **(B)** Eigenvalue is low, predicting the requirement of low energy to deform the protein. **(C)** Elastic network model showing the stiffness of the protein chain with darker gray. **(D)** Covariance matrix analysis showing that most of the atoms in the protein structure have correlated (red color) and anti-correlated (blue) motions.

iMODs was used to estimate the protein–ligand docking to determine the deformability in the main chain and the deformed nature of the residues. Some higher peaks for compound **5j** and eigenvalues state the stiffness of the model shown in Figure 7A. Its eigenvalue was low, explaining that low energy is required to deform the structure. The graphs shown in Figure 7B represent the eigenvalues of **5j**, i.e., 3.085545e-05. The elastic network model is shown in Figure 7C, where each dot represents one spring and pair of atoms. It further explains the stiffness and spring stiffness of compound **5j** to the protein. In addition, Figure 7D represents the covariance matrix, in which the motion of atoms can be seen as correlated and anti-correlated because the red and blue regions are more obvious than the white regions.

### 2.6 Computational ADMET analysis

SwissADME was applied to assess the drug-likeness characteristics of compounds **5j, 5l, 5m, 7k**, and **7i** with respect to urease. The pharmacokinetic properties of **5j, 5l, 5m, 7k**, and **7i** were examined by interpreting the results based on the compound structure. In this case, the evaluation indicated that all these compounds could serve as a lead since they met all the specified parameters. Compounds **5j, 5l**, and **5m** showed molecular weights of 431.53, 292.78, and 447.53 g/mol, respectively, their topological polar surface areas (TPSAs) were 151.68, 69.70, and 160.91, respectively, and the consensus log P _**o/w**_ was lower than 5. They further showed synthetic accessibility of 3.43, 2.86, and 3.44, as shown in Table 3. The ADMET analysis of compounds **7k** and **7i** showed that they have a molecular weight of 507.63 and 538.60 g/mol, respectively. It further showed that that the TPSAs were 151.68 and 197.50 Å^2^, respectively, and the consensus log P _**o/w**_ was lower than 5, as shown in Table 3.

**TABLE 3:** ADMET analysis for determining the drug likeliness and lead likeliness properties of compounds **5j, 5l, 5m, 7k**, and **7i** against urease.

Compound	5j	5l	5m	7k	7i
Formula	C_19_H_21_N_5_O_3_S_2_	C_14_H_13_ CN_2_OS	C_19_H_21_N_5_O_4_S_2_	C_25_H_25_N_5_O_3_S_2_	C_24_H_22_N_6_O_5_S_2_
Molecular weight (g/mol)	431.53	292.78	447.53	507.63	538.60
Molar refractivity (m^3^mol^-1^)	128.31	80.27	129.83	151.68	157.03
TPSA (Å^2^)	151.68	69.70	160.91	151.68	197.50
Consensus log P _o/w_	2.12	3.73	1.78	3.43	2.58
Class	Moderately soluble	Moderately soluble	Moderately soluble	Moderately soluble	Moderately soluble
GI absorption	Low	High	Low	Low	Low
BBB permeant	No	Yes	No	No	No
P-gp substrate	No	No	No	No	No
CYP1A2 inhibitor	No	Yes	Yes	Yes	Yes
CYP2C19 inhibitor	Yes	Yes	Yes	Yes	Yes
CYP2C9 inhibitor	No	Yes	Yes	Yes	Yes
CYP2D6 inhibitor	Yes	No	Yes	Yes	No
CYP3A4 inhibitor	Yes	No	Yes	Yes	Yes
Log Kp (skin permeation) (cm/s)	−6.65	−5.17	−7.02	−6.10	−6.67
Lipinski	Yes; 0 violation	Yes; 0 violation	Yes; 1 violation	Yes; 1 violation	No, 2 violations
Lead likeness	No	No; 1 violation	No; 2 violations	No; 3 violations	No, 3 violations
Bioavailability score	0.55	0.55	0.55	0.55	0.17
PAINS	0 alert	0 alert	0 alert	0 alert	0 alert
Synthetic accessibility	3.43	2.86	3.44	3.73	3.70

## 3 Materials and methods

### 3.1 General

All the chemicals, reagents, and solvents were purchased from Alfa Aesar (Kandel, Germany) and utilized without any further purification. The ^1^H NMR (500 MHz) and ^13^C NMR (125 MHz) spectra were recorded in dimethyl sulfoxide (DMSO) using a Bruker DPX spectrophotometer (Bruker; Zürich, Switzerland). The chemical shifts were recorded in ppm reference to tetramethylsilane. Thin-layer chromatography (CHCl_3_/MeOH) was used in combination with a Spectroline E-Series UV lamp to monitor the progress of chemical reactions (Alfa Aesar, Kandel, Germany). Melting points were recorded on a Gallenkamp instrument (Fisons; Uckfield, United Kingdom). Compounds **2**, **5a**–**5o**, and **7a**–**k** were produced according to Scheme 1.

### 3.2 Procedure for 1-(3-nitropyridin-2-yl)piperazine (**3**)

A homogenous solution of piperazine (**2**) **(**40.5 g, 472 mmol) was prepared in acetonitrile (100 ml) in a round-bottom flask (solution A). On the other hand, 15 g (94.30 mmol) of 2-chloro-3-nitropyridine (**1**) was dissolved in 50 ml of acetonitrile in a beaker (solution B). Solution B was added dropwise to solution A under constant stirring. The reaction mixture was refluxed for 12 h, and the reaction progress was monitored using TLC. On completion of the reaction, the reaction mixture was cooled to room temperature, and 100 ml of ice-cold distilled water was added to it. From the mixture, 1-(3-nitropyridin-2-yl)piperazine (**3**) was extracted with chloroform and further purified by column chromatography with CHCl_3_/MeOH. On evaporating the solvent, yellow crystals of (**3**) were produced with 65% yield.

#### 3.2.1 1-(3-Nitropyridin-2-yl)piperazine (**3**)


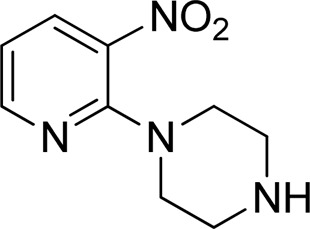


Yield, 65%; brownish–yellow solid; mp: 77°C–79°C. ^1^H NMR (*δ*): 8.29–8.30 (m, 1H, Ar-H), 8.15 (dd, 1H, *J =* 7.95 Hz, 1.8 Hz, Ar-H), 6.82–6.84 (m, 1H, Ar-H), 4.2 (t, 4H, *J =* 5 Hz, piperazinyl), 3.23–3.25 (m, 4H, piperazinyl), and 2.50–2.51 (m, 1H, NH). ^13^C NMR (*δ*): 152.6679 (NCH), 152.5184 (N=C-N), 136.4210 (C(CH)_2_), 132.7990 (CH(C)C), 114.3262 (CHCH), 48.7025 (NCH_2_CH_2_), and 45.0161(NHCH_2_CH_2_). Elemental analysis of C_9_H_12_N_4_O_2_: calculated: C, 51.92; H, 5.81; and N, 26.9; found: C, 51.97; H, 5.77; and N, 26.88%.

### 3.3 Procedure for the synthesis of 2-oxo-2-(arylamino)ethyl 4-(3-nitropyridin-2-yl)piperazine-1-carbodithioates **5a**–**5n**

A mixture of 1-(3-nitropyridin-2-yl) piperazine (**3**) (0.15 mmol) and NaOAc (0.30 mmol) was prepared in 15 ml of acetonitrile. The mixture was stirred for 15 min, and CS_2_ (0.30 mmol) was added dropwise under constant stirring for 30 min at room temperature. On the other hand, a solution of 2-chloro-*N*-arylacetamides **4a**–**4o** (0.15 mmol) was prepared in 5 ml of acetonitrile and added to the above mixture. The resulting mixture was refluxed for 8–16 h under stirring and monitored using TLC. Finally, the addition of ice-cold water resulted in yellow–orange precipitates of 2-oxo-2-(arylamino)ethyl 4-(3-nitropyridin-2-yl) piperazine-1-carbodithioate derivatives **5a**–**5o**. The ppts were collected and purified by column chromatography.

#### 3.3.1 2-Oxo-2-(phenylamino)ethyl 4-(3-nitropyridin-2-yl)piperazine-1-carbodithioate (**5a**)


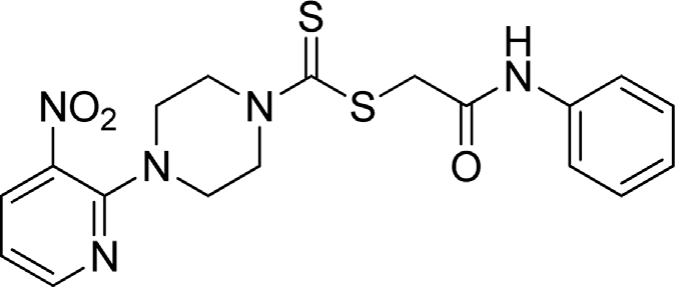


Yield, 75%; yellow solid; mp: 119°C–120°C. ^1^H NMR (DMSO-*d*_*6*_ 500 MHz) δ (ppm): 10.27 (s, 1H, NH), 8.45–8.46 (m, 1H, Ar-H), 8.30 (dd, 1H, *J =* 6.65 Hz, 1.42 Hz, Ar-H), 7.57 (d, 2H, *J =* 7.25 Hz, Ar-H), 7.31 (t, 2H, *J =* 6.45 Hz, Ar-H), 7.05 (t, 1H, *J =* 6.85 Hz, Ar-H), 6.96 (q, 1H, *J =* 6.70 Hz, Ar-H), 4.32 (Br s, 2H, piperazinyl), 4.26 (s, 2H, methylene), 4.14 (Br s, 2H, piperazinyl), and 3.6 (Br s, 4H, piperazinyl). Elemental analysis of C_18_H_19_N_5_O_3_S_2_: calculated: C, 51.78; H, 4.59; and N, 16.77; found: C, 51.75; H, 4.63; and N, 16.75%.

#### 3.3.2 2-((2-Chlorophenyl)amino)-2-oxoethyl 4-(3-nitropyridin-2-yl)piperazine-1-carbodithioate (**5b**)


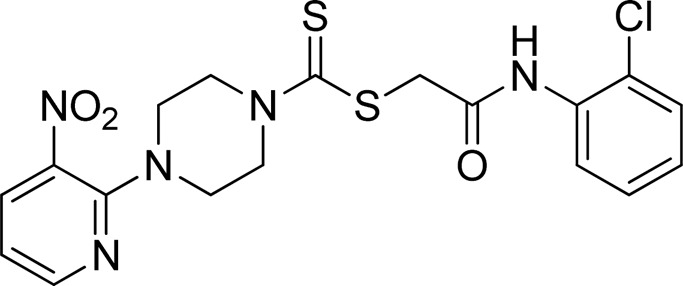


Yield, 68%; yellow solid; mp: 114°C–116°C. ^1^H NMR (DMSO-*d*_*6*_ 500 MHz) δ (ppm): 9.74 (s, 1H, NH), 8.45–8.46 (m, 1H, Ar-H), 8.30 (dd, 1H, *J =* 6.7 Hz, 1.35 Hz, Ar-H), 7.76 (d, 1H, *J =* 6.75 Hz, Ar-H), 7.49 (dd, 1H, *J =* 6.7 Hz, 1.20 Hz, Ar-H), 7.33 (t, 1H, *J =* 6.45 Hz, Ar-H), 7.18 (t, 1H, *J =* 7.75, Ar-H), 6.96 (q, 1H, *J =* 7.70 Hz, Ar-H), 4.35 (s, 2H, methylene), 4.33 (Br s, 2H, piperazinyl), 4.14 (Br s, 2H, piperazinyl), and 3.6 (Br s, 4H, piperazinyl). ^13^C NMR (DMSO-*d*_*6*_ 125 MHz) δ (ppm): 194.9 (N(C=S)S), 166.4 (C=O), 152.6 (Ar-C), 152.0 (Ar-C), 136.3 (Ar-C), 135.1 (Ar-C), 132.6 (Ar-C), 129.9 (Ar-C), 127.9 (Ar-C), 126.7 (Ar-C), 126.3 (Ar-C), 125.8 (Ar-C), 114.4 (Ar-C), 50.8 (piperazine, NCH_2_), 49.0 (piperazine, NCH_2_), 46.4 (piperazine, 2 x NCH_2_), and 40.8 (SCH_2_(CO)). Elemental analysis of C_18_H_18_ClN_5_O_3_S_2_: calculated: C, 47.84; H, 4.01; and N, 15.50; found: C, 47.88; H, 4.04; and N, 15.47%.

#### 3.3.3 2-((3-Chlorophenyl)amino)-2-oxoethyl 4-(3-nitropyridin-2-yl)piperazine-1-carbodithioate (**5c**)


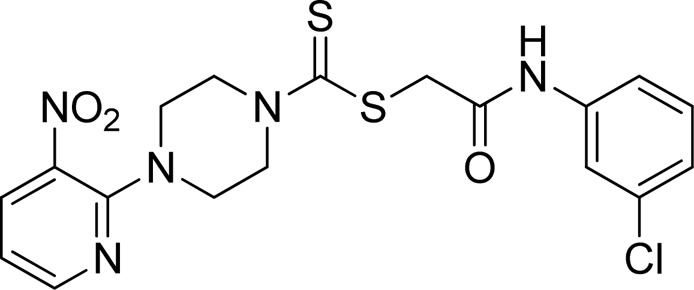


Yield, 65%; yellow solid; mp: 121°C–122°C. ^1^H NMR (DMSO-*d*_*6*_ 500 MHz) δ (ppm): 10.48 (s, 1H, NH), 8.44–8.46 (m, 1H, Ar-H), 8.30 (dd, 1H, *J =* 6.7 Hz, 1.45 Hz, Ar-H), 7.78 (s, 1H, Ar-H), 7.44 (d, 1H, *J =* 6.90 Hz, Ar-H), 7.34 (t, 1H, *J =* 6.70 Hz, Ar-H), 7.11 (d, 1H, *J =* 6.70 Hz, Ar-H), 6.96 (q, 1H, *J =* 6.7 Hz, Ar-H), 4.31 (Br s, 2H, piperazinyl), 4.29 (Br s, 2H, methylene), 4.13 (Br s, 2H, piperazinyl), and 3.6 (Br s, 4H, piperazinyl). Elemental analysis of C_18_H_18_ClN_5_O_3_S_2_: calculated: C, 47.84; H, 4.01; and N, 15.50; found: C, 47.80; H, 4.05; and N, 15.55%.

#### 3.3.4 2-((4-Chlorophenyl)amino)-2-oxoethyl 4-(3-nitropyridin-2-yl)piperazine-1-carbodithioate (**5d**)


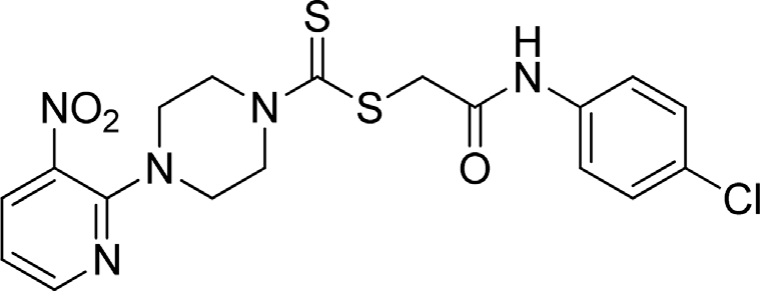


Yield, 72%; yellow solid; mp: 143°C–144°C. ^1^H NMR (DMSO-*d*_*6*_ 500 MHz) δ (ppm): 10.41 (s, 1H, NH), 8.45–8.46 (m, 1H, Ar-H), 8.30 (dd, 1H, *J =* 6.7 Hz, 1.40 Hz, Ar-H), 7.61 (d, 2H, *J =* 7.4 Hz, Ar-H), 7.36 (d, 2H, *J =* 7.4, Ar-H), 6.96 (q, 1H, *J =* 6.70 Hz, Ar-H), 4.31 (Br s, 2H, piperazinyl), 4.28 (s, 2H, methylene), 4.13 (Br s, 2H, piperazinyl), and 3.6 (Br s, 4H, piperazinyl). Elemental analysis of C_18_H_18_ClN_5_O_3_S_2_: calculated: C, 47.84; H, 4.01; and N, 15.50; found: C, 47.89; H, 3.98; and N, 15.54%.

#### 3.3.5 2-((3-Bromophenyl)amino)-2-oxoethyl 4-(3-nitropyridin-2-yl)piperazine-1-carbodithioate (**5e**)


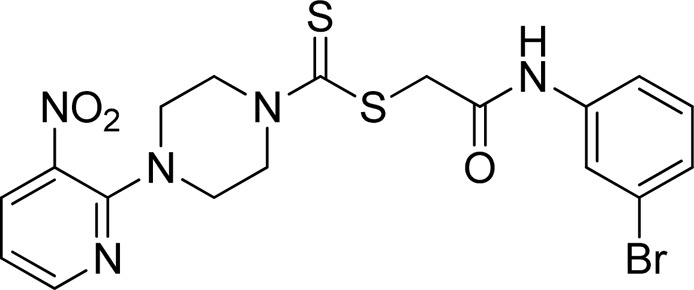


Yield, 72%; yellow solid; mp: 110°C–112°C. ^1^H NMR (DMSO-*d*_*6*_ 500 MHz) δ (ppm): 10.46 (s, 1H, NH), 8.45–8.46 (m, 1H, Ar-H), 8.30 (dd, 1H, *J =* 6.7 Hz, 1.40 Hz, Ar-H), 7.93 (s, 1H, Ar-H), 7.48 (d, 1H, *J =* 6.65 Hz, Ar-H), 7.23–7.29 (m, 2H, Ar-H), 6.96 (q, 1H, *J =* 6.7 Hz, Ar-H), 4.31 (Br s, 2H, piperazinyl), 4.26 (s, 2H, methylene), 4.13 (Br s, 2H, piperazinyl), and 3.6 (Br s, 4H, piperazinyl). Elemental analysis of C_18_H_18_BrN_5_O_3_S_2_: calculated: C, 43.55; H, 3.65; and N, 14.11; found: C, 43.51; H, 3.70; and N, 14.13%.

#### 3.3.6 2-((4-Bromophenyl)amino)-2-oxoethyl 4-(3-nitropyridin-2-yl)piperazine-1-carbodithioate (**5f**)


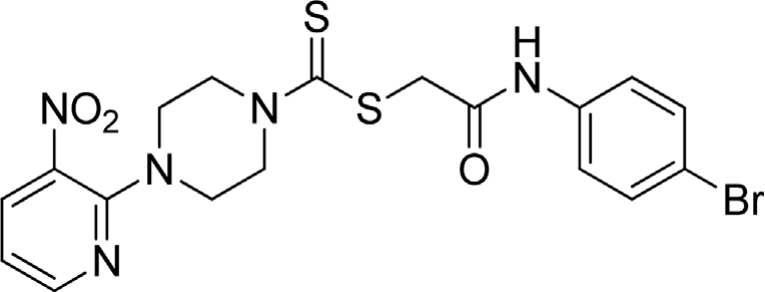


Yield, 76%; yellow solid; mp: 136°C–138°C. ^1^H NMR (DMSO-*d*_*6*_ 500 MHz) δ (ppm): 10.48 (s, 1H, NH), 8.45–8.46 (m, 1H, Ar-H), 8.33 (dd, 1H, *J =* 6.65 Hz, 1.40 Hz, Ar-H), 7.95 (s, 1H, Ar-H), 7.48 (d, 1H, *J =* 6.70 Hz, Ar-H), 7.28–7.33 (m, 2H, Ar-H), 6.98 (q, 1H, *J =* 6.65 Hz, Ar-H), 4.33 (Br s, 2H, piperazinyl), 4.29 (s, 2H, methylene), 4.15 (Br s, 2H, piperazinyl), and 3.62 (Br s, 4H, piperazinyl). Elemental analysis of C_18_H_18_BrN_5_O_3_S_2_: calculated: C, 43.55; H, 3.65; and N, 14.11; found: C, 43.51; H, 3.68; and N, 14.13%.

#### 3.3.7 2-((2-Nitrophenyl)amino)-2-oxoethyl 4-(3-nitropyridin-2-yl)piperazine-1-carbodithioate (**5g**)


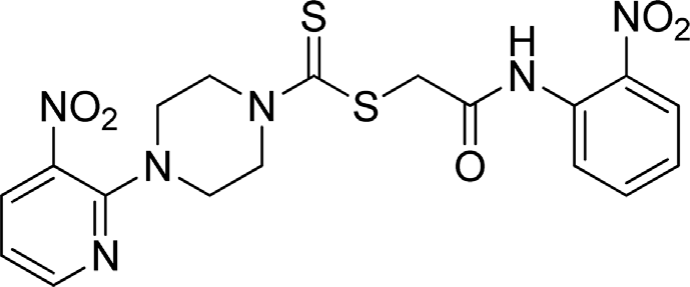


Yield, 82%; yellow solid; mp: 149°C–151°C. ^1^H NMR (DMSO-*d*_*6*_ 500 MHz) δ (ppm): 10.65 (s, 1H, NH), 8.44–8.46 (m, 1H, Ar-H), 8.32 (dd, 1H, *J =* 6.75 Hz, 1.35 Hz, Ar-H), 8.03 (d, 1H, *J =* 6.70 Hz, Ar-H), 7.85 (d, 1H, *J =* 6.95 Hz, Ar-H), 7.78 (t, 1H, *J =* 6.85 Hz, Ar-H), 7.35 (t, 1H, *J =* 6.75, Ar-H), 6.95 (d, 1H, *J =* 6.65 Hz, Ar-H), 4.34 (Br s, 2H, methylene), 4.26 (Br s, 2H, piperazinyl), 4.17 (Br s, 2H, piperazinyl), and 3.59 (Br s, 4H, piperazinyl). ^13^C NMR (DMSO-*d*_*6*_ 125 MHz) δ (ppm): 194.4 (N(C=S)S), 166.5 (C=O), 152.6 (Ar-C), 152.0 (Ar-C), 141.4 (Ar-C), 136.3 (Ar-C), 134.9 (Ar-C), 132.7 (Ar-C), 131.9 (Ar-C), 125.6 (2 × Ar-C), 124.9 (Ar-C), 114.4 (Ar-C), 50.9 (piperazine, NCH_2_), 49.1 (piperazine, NCH_2_), 46.4 (piperazine, 2 × NCH_2_), and 40.8 (SCH_2_(CO)). Elemental analysis of C_18_H_18_N_6_O_5_S_2_: calculated: C, 46.74; H, 3.92; and N, 18.17; found; C, 46.77; H, 3.89; and N, 18.14%.

#### 3.3.8 2-((3-Nitrophenyl)amino)-2-oxoethyl 4-(3-nitropyridin-2-yl)piperazine-1-carbodithioate (**5h**)


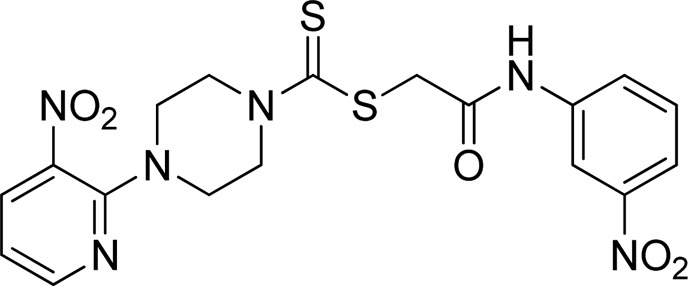


Yield, 85%; yellow solid; mp: 174°C–175°C. ^1^H NMR (DMSO-*d*_*6*_ 500 MHz) δ (ppm): 10.63 (s, 1H, NH), 8.45–8.47 (m, 1H, Ar-H), 8.31 (dd, 1H, *J =* 6.7 Hz, 1.40 Hz, Ar-H), 8.01 (dd, 1H, *J =* 6.85 Hz, 1.25 Hz, Ar-H), 7.88 (d, 1H, *J =* 6.90 Hz, Ar-H), 7.73 (t, 1H, *J =* 6.75 Hz, Ar-H), 7.37 (t, 1H, *J =* 6.90, Ar-H), 6.96 (d, 1H, *J =* 6.7 Hz, Ar-H), 4.32 (Br s, 2H, methylene), 4.29 (Br s, 2H, piperazinyl), 4.15 (Br s, 2H, piperazinyl), and 3.61 (Br s, 4H, piperazinyl). Elemental analysis of C_18_H_18_N_6_O_5_S_2_: calculated: C, 46.74; H, 3.92; and N, 18.17; found: C, 46.79; H, 3.97; and N, 18.21%.

#### 3.3.9 2-((4-Nitrophenyl)amino)-2-oxoethyl 4-(3-nitropyridin-2-yl)piperazine-1-carbodithioate (**5i**)


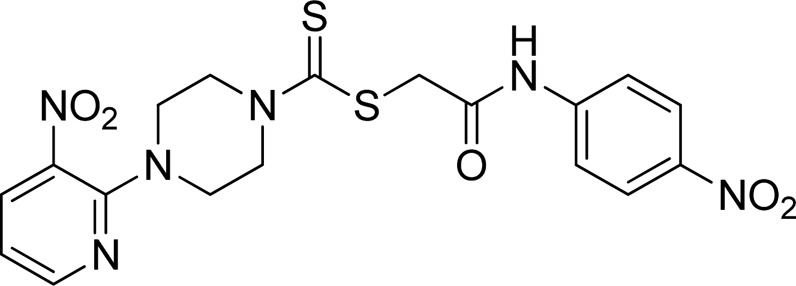


Yield, 88%; yellow solid; mp: 168°C–169°C. ^1^H NMR (500 MHz) δ (ppm): 10.92 (s, 1H, NH), 8.46–8.47 (m, 1H, Ar-H), 8.31 (dd, 1H, *J =* 6.7 Hz, 1.40 Hz, Ar-H), 8.23 (d, 2H, *J =* 7.7 Hz, Ar-H), 7.83 (d, 2H, *J =* 7.7 Hz, Ar-H), 6.96 (q, 1H, *J =* 6.7 Hz, Ar-H), 4.36 (s, 2H, methylene), 4.31 (Br s, 2H, piperazinyl), 4.14 (Br s, 2H, piperazinyl), and 3.6 (Br s, 4H, piperazinyl). Elemental analysis of C_18_H_18_N_6_O_5_S_2_: calculated: C, 46.74; H, 3.92; and N, 18.17; found: C, 46.78; H, 3.94; and N, 18.12%.

#### 3.3.10 2-Oxo-2-(o-tolylamino)ethyl 4-(3-nitropyridin-2-yl)piperazine-1-carbodithioate (**5j**)


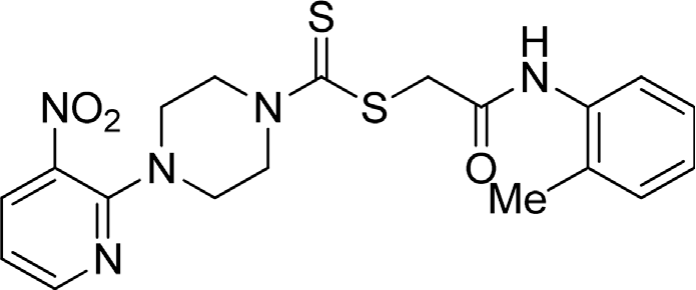


Yield, 71%; yellow solid; mp: 141°C–143°C. ^1^H NMR (DMSO-*d*_*6*_ 500 MHz) δ (ppm): 9.56 (s, 1H, NH), 8.46–8.47 (m, 1H, Ar-H), 8.31 (dd, 1H, *J =* 6.7 Hz, 1.45 Hz, Ar-H), 7.37 (d, 1H, *J =* 6.6 Hz, Ar-H), 7.20 (d, 1H, *J =* 6.2 Hz, Ar-H), 7.16 (t, 1H, *J =* 6.85 Ar-H), 7.09 (t, 1H, *J =* 6.2 Hz, Ar-H), 6.96 (q, 1H, *J =* 6.7 Ar-H), 4.33 (Br s, 2H, piperazinyl), 4.30 (s, 2H, methylene), 4.14 (Br s, 2H, piperazinyl), 3.6 (Br s, 4H, piperazinyl), and 2.22 (Br s, 3H, methyl). Elemental analysis of C_19_H_21_N_5_O_3_S_2_: calculated: C, 52.88; H, 4.91; and N, 16.23; found: C, 52.84; H, 4.93; and N, 16.26%.

#### 3.3.11 2-Oxo-2-(m-tolylamino)ethyl 4-(3-nitropyridin-2-yl)piperazine-1-carbodithioate (**5k**)


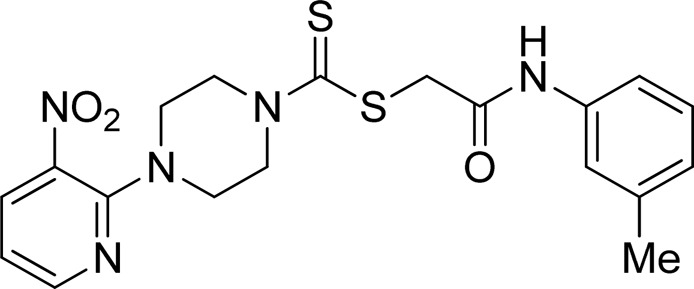


Yield, 75%; yellow solid; mp: 101°C–103°C. ^1^H NMR (DMSO-*d*_*6*_ 500 MHz) δ (ppm): 10.19 (s, 1H, NH), 8.45–8.46 (m, 1H, Ar-H), 8.30 (dd, 1H, *J =* 6.7 Hz, 1.45 Hz, Ar-H), 7.42 (s, 1H, Ar-H), 7.37 (d, 1H, *J =* 6.90 Ar-H), 7.18 (t, 1H, *J =* 6.5 Hz, Ar-H), 6.96 (q, 1H, *J =* 6.7 Hz, Ar-H), 6.87 (d, 1H, *J =* 6.95 Hz, Ar-H), 4.32 (Br s, 2H, piperazinyl), 4.28 (s, 2H, methylene), 4.14 (Br s, 2H, piperazinyl), 3.6 (Br s, 4H, piperazinyl), and 2.27 (Br s, 3H, methyl). ^13^C NMR (DMSO-*d*_*6*_ 125 MHz) δ (ppm): 195.3 (N(C=S)S), 165.6 (C=O), 152.5 (Ar-C), 152.0 (Ar-C), 139.3 (Ar-C), 138.4 (Ar-C), 136.3 (Ar-C), 132.7 (Ar-C), 129.0 (Ar-C), 124.5 (Ar-C), 120.1 (Ar-C), 116.8 (Ar-C), 114.4 (Ar-C), 50.6 (piperazine, NCH_2_), 48.8 (piperazine, NCH_2_), 46.8 (piperazine, 2 × NCH_2_), 41.6 (SCH_2_(CO)), and 21.6 (CH_3_). Elemental analysis of C_19_H_21_N_5_O_3_S_2_: calculated: C, 52.88; H, 4.91; N, 16.23; found: C, 52.93; H, 4.95; and N, 16.19%.

#### 3.3.12 2-Oxo-2-(p-tolylamino)ethyl 4-(3-nitropyridin-2-yl)piperazine-1-carbodithioate (**5l**)


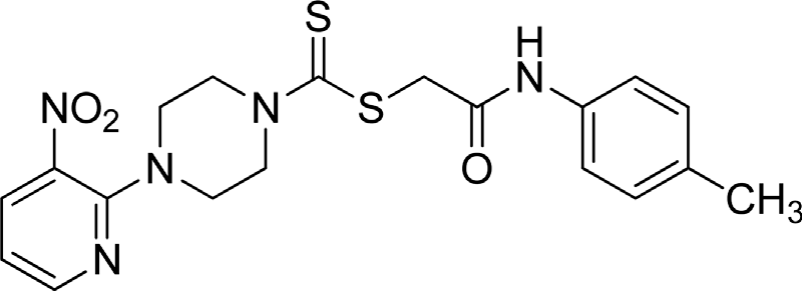


Yield, 69%; yellow solid; mp: 145°C–146°C. ^1^H NMR (DMSO-*d*_*6*_ 500 MHz) δ (ppm): 10.17 (s, 1H, NH), 8.45–8.46 (m, 1H, Ar-H), 8.30 (dd, 1H, *J =* 6.7 Hz, 1.1 Hz, Ar-H), 7.46 (d, 2H, *J =* 7.00 Hz Ar-H), 7.11 (d, 2H, *J =* 7.35 Hz, Ar-H), 6.96 (q, 1H, *J =* 6.70 Hz, Ar-H), 4.31 (Br s, m, 2H, piperazinyl), 4.26 (s, 2H, methylene), 4.12 (Br s, m, 2H, piperazinyl), 3.6 (Br s, 4H, piperazinyl), and 2.24 (Br s, 3H, methyl). Elemental analysis of C_19_H_21_N_5_O_3_S_2_: calculated: C, 52.88; H, 4.91; and N, 16.23; found: C, 52.92; H, 4.86; and N, 16.26%.

#### 3.3.13 2-((2-Methoxyphenyl)amino)-2-oxoethyl 4-(3-nitropyridin-2-yl)piperazine-1-carbodithioate (**5m**)


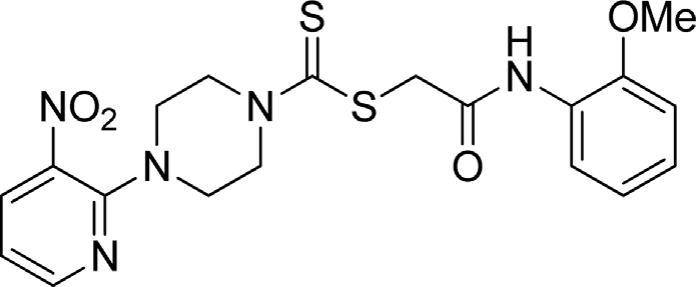


Yield, 62%; yellow solid; mp: 137°C–138°C. ^1^H NMR (DMSO-*d*_*6*_ 500 MHz) δ (ppm): 9.40 (s, 1H, NH), 8.45–8.46 (m, 1H, Ar-H), 8.30 (dd, 1H, *J =* 6.7 Hz, 1.40 Hz, Ar-H), 7.98 (d, 1H, *J =* 6.6 Hz, Ar-H), 7.03–7.08 (m, 2H, Ar-H), 6.96 (q, 1H, *J =* 6.7 Hz, Ar-H), 6.90 (t, 1H, *J =* 6.70 Ar-H), 4.33 (s, 2H, methylene), 4.29 (Br s, 2H, piperazinyl, 4.14 (Br s, 2H, piperazinyl), 3.38 (s, 3H, methyl), and 3.6 (Br s, 4H, piperazinyl). ^13^C NMR (DMSO-*d*_*6*_ 125 MHz) δ (ppm): 195.0 (N(C=S)S), 165.9 (C=O), 152.6 (Ar-C), 152.0 (Ar-C), 149.5 (Ar-C), 136.38 (Ar-C), 132.7 (Ar-C), 127.4 (Ar-C), 124.8 (Ar-C), 121.3 (Ar-C), 120.8 (Ar-C), 114.4 (Ar-C), 111.6 (Ar-C), 56.2 (OCH_3_), 50.9 (piperazine, NCH_2_), 49.0 (piperazine, NCH_2_), 46.4 (piperazine, 2 × NCH_2_), and 41.1 (SCH_2_(CO)). Elemental analysis of C_19_H_21_N_5_O_4_S_2_: calculated: C, 50.99; H, 4.73; and N, 15.65; found: C, 51.02; H, 4.69; and N, 15.68%.

#### 3.3.14 2-((4-Methoxyphenyl)amino)-2-oxoethyl 4-(3-nitropyridin-2-yl)piperazine-1-carbodithioate (**5n**)


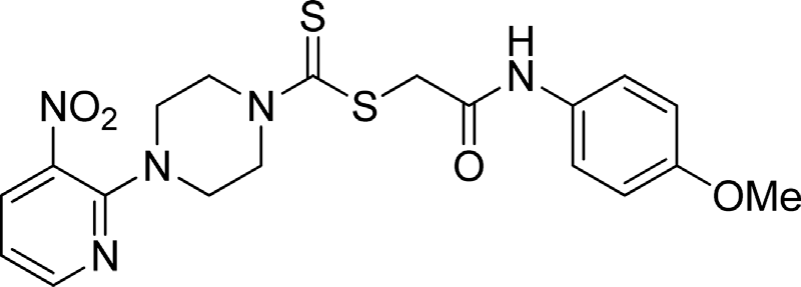


Yield, 66%; yellow solid; mp: 151°C–153°C. ^1^H NMR (DMSO-*d*_*6*_ 500 MHz) δ (ppm): 10.12 (s, 1H, NH), 8.45–8.46 (m, 1H, Ar-H), 8.30 (dd, 1H, *J =* 6.7 Hz, 1.40 Hz, Ar-H), 7.48 (d, 2H, *J =* 7.55 Hz, Ar-H), 6.96 (q, 1H, *J =* 6.70 Hz Ar-H), 6.88 (d, 2H, *J =* 7.5 Hz, Ar-H), 4.32 (Br s, 2H, piperazinyl), 4.25 (s, 2H, methylene), 4.13 (Br s, 2H, piperazinyl), 3.71 (s, 3H, methyl), and 3.60 (Br s, 4H, piperazinyl). Elemental analysis of C_19_H_21_N_5_O_4_S_2_: calculated: C, 50.99; H, 4.73; and N, 15.65; found: C, 51.03; H, 4.69; and N, 15.67%.

### 3.4 Procedure for the synthesis of 4-((aryl)carbamoyl)benzyl 4-(3-nitropyridin-2-yl)piperazine-1-carbodithioates **7a**–**7n**

A mixture of 1-(3-nitropyridin-2-yl)piperazine (**3**) (0.15 mmol), NaOAc (0.30 mmol), and CS_2_ (0.30 mmol) was prepared in 15 ml of acetonitrile in a round-bottom flask (solution A). On the other hand, a solution of 4-(chloromethyl)-*N*-arylbenzamide (**6a**–**6n)** (0.15 mmol) was prepared in 5 ml of acetonitrile (solution B). Solution B was added to solution A, and the resulting mixture was refluxed for 12–24 h with constant stirring. The reaction was monitored with TLC. Finally, the addition of water resulted in the precipitates of 4-((aryl)carbamoyl)benzyl 4-(3-nitropyridin-2-yl)piperazine-1-carbodithioate derivatives **7a**–**7n**. The ppts were collected and purified by column chromatography.

#### 3.4.1 4-(Phenylcarbamoyl)benzyl 4-(3-nitropyridin-2-yl)piperazine-1-carbodithioate (**7a**)


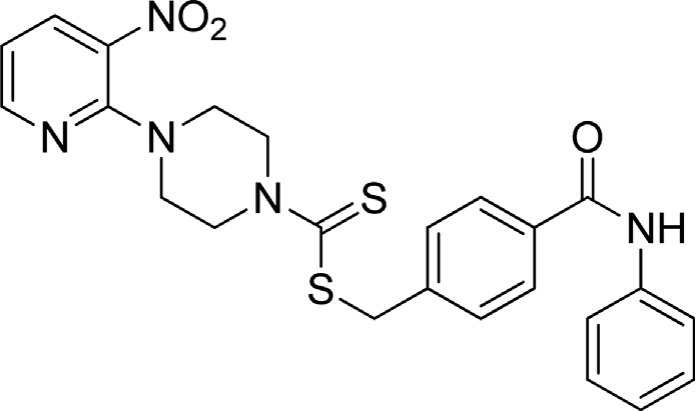


Yield, 63%; yellow solid; mp: 142°C–143°C. ^1^H NMR (DMSO-*d*_*6*_ 500 MHz) δ (ppm): 10.08 (s, 1H, NH), 8.44–8.45 (m, 1H, Ar-H), 8.30 (dd, 1H, *J =* 6.65 Hz, 1.4 Hz, Ar-H), 7.89 (d, 2H, *J =* 6.75 Hz, Ar-H), 7.76 (d, 2H, *J =* 7.35 Hz, Ar-H), 7.55 (d, 2H, *J =* 6.67 Hz, Ar-H), 7.36 (t, 2H, *J =* 6.5 Hz, Ar-H), 7.10 (t, 1H, *J =* 7.05, Ar-H), 6.95 (q, 1H, *J =* 6.70 Hz, Ar-H), 4.66 (s, 2H, methylene), 4.34 (Br s, 2H, piperazinyl), 4.07 (Br s, 2H, piperazinyl), and 3.57 (Br s, 4H, piperazinyl). Elemental analysis of C_24_H_23_N_5_O_3_S_2_: calculated: C, 57.93; H, 5.47; and N, 14.07; found: C, 57.89; H, 5.45; and N, 14.11%.

#### 3.4.2 4-((2-Chlorophenyl)carbamoyl)benzyl 4-(3-nitropyridin-2-yl)piperazine-1-carbodithioate (**7b**)


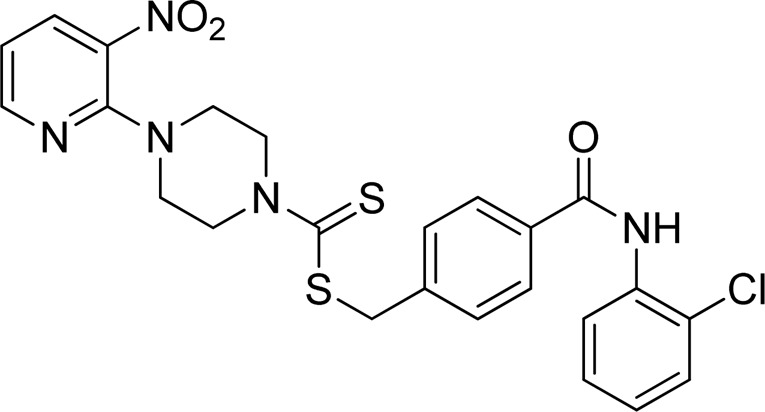


Yield, 61%; yellow solid; mp: 109°C–110°C. ^1^H NMR (DMSO-*d*_*6*_ 500 MHz) δ (ppm): 10.02 (s, 1H, NH), 8.44–8.45 (m, 1H, Ar-H), 8.29–8.31 (dd, 1H, *J =* 6.7 Hz, 1.35 Hz, Ar-H), 7.94 (d, 2H, *J =* 7.00 Hz, Ar-H), 7.60 (dd, 1H, *J =* 6.60 Hz, 1.30 Hz, Ar-H), 7.55 (t, 3H, *J =* 6.90 Hz, Ar-H), 7.38 (t, 1H, *J =* 6.60 Hz, Ar-H), 7.29 (t, 1H, *J =* 6.50 Hz, Ar-H), 6.95 (d, 1H, *J =* 6.70 Hz, Ar-H), 4.68 (s, 2H, methylene), 4.34 (Br s, 2H, piperazinyl), 4.09 (Br s, 2H, piperazinyl), and 3.58 (Br s, 4H, piperazinyl). ^13^C NMR (DMSO-*d*_*6*_ 125 MHz) δ (ppm): 195.2 (N(C=S)S), 165.6 (C=O), 152.5 (Ar-C), 152.0 (Ar-C), 141.19 (Ar-C), 136.3 (Ar-C), 133.3 (Ar-C), 132.7 (Ar-C), 130.0 (Ar-C), 129.8 (2 × Ar-C), 129.7 (Ar-C), 128.8 (2 × Ar-C), 128.2 (Ar-C), 127.9 (2 × Ar-C), 114.4 (Ar-C), 50.8 (piperazine, NCH_2_), 49.0 (piperazine, NCH_2_), 46.4 (piperazine, 2 × NCH_2_), and 40.8 (SCH_2_). Elemental analysis of C_24_H_22_ClN_5_O_3_S_2_: calculated: C, 54.59; H, 4.20; and N, 13.26; found: C, 54.55; H, 4.24; and N, 13.30%.

#### 3.4.3 4-((3-Chlorophenyl)carbamoyl)benzyl 4-(3-nitropyridin-2-yl)piperazine-1-carbodithioate (**7c**)


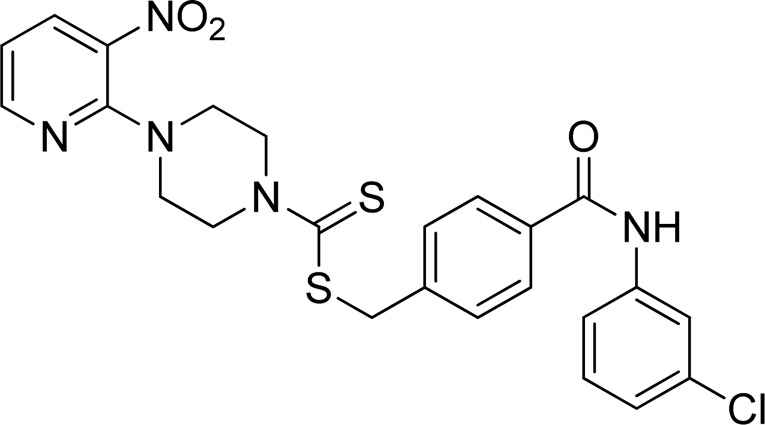


Yield, 56%; yellow solid; mp: 140°C–141°C. ^1^H NMR (DMSO-*d*_*6*_ 500 MHz) δ (ppm): 10.38 (s, 1H, NH), 8.44–8.45 (m, 1H, Ar-H), 8.29 (dd, 1H, *J =* 6.5 Hz, 1.4 Hz, Ar-H), 7.97 (s, 1H, Ar-H), 7.90 (d, 2H, *J =* 6.85 Hz, Ar-H), 7.70–72 (m, 1H, Ar-H), 7.56–7.57 (m, 2H, Ar-H), 7.38 (t, 1H, *J =* 6.5 Hz, Ar-H), 7.15 (dd, 1H, *J =* 6.7 Hz, 1.05 Hz, Ar-H), 6.95 (q, 1H, *J =* 6.65 Hz, Ar-H), 4.68 (s, 2H, methylene), 4.35 (Br s, 2H, piperazinyl), 4.09 (Br s, 2H, piperazinyl), and 3.59 (Br s, 4H, piperazinyl). Elemental analysis of C_24_H_22_ClN_5_O_3_S_2_: calculated: C, 54.59; H, 4.20; and N, 13.26; found: C, 54.64; H, 4.23; and N, 13.27%.

#### 3.4.4 4-((4-Chlorophenyl)carbamoyl)benzyl 4-(3-nitropyridin-2-yl)piperazine-1-carbodithioate (**7d**)


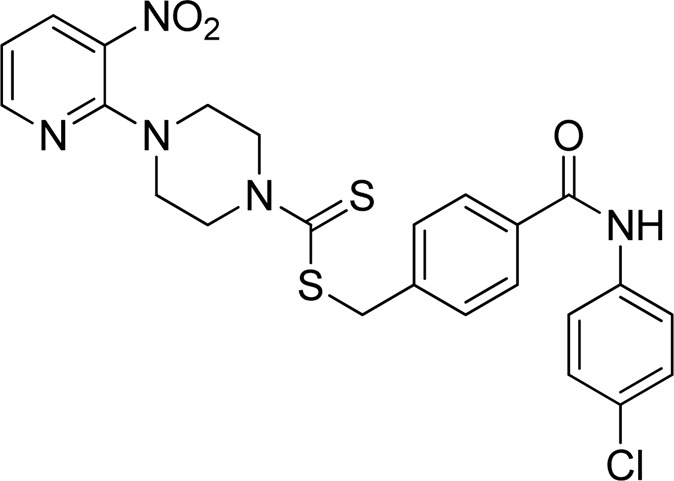


Yield, 53%; yellow solid; mp: 147°C–148°C. ^1^H NMR (DMSO-*d*_*6*_ 500 MHz) δ (ppm): 10.34 (s, 1H, NH), 8.44–8.45 (m, 1H, Ar-H), 8.29 (dd, 1H, *J =* 6.75 Hz, 1.4 Hz, Ar-H), 7.89 (d, 2H, *J =* 6.85 Hz, Ar-H), 7.80 (d, 2H, *J =* 7.35 Hz, Ar-H), 7.56 (d, 2H, *J =* 6.8 Hz, Ar-H), 7.40 (d, 2H, *J =* 7.35 Hz, Ar-H), 6.95 (q, 1H, *J =* 6.7 Hz, Ar-H), 4.67 (s, 2H, methylene), 4.34 (Br s, 2H, piperazinyl), 4.08 (Br s, 2H, piperazinyl), and 3.58 (Br s, 4H, piperazinyl). ^13^C NMR (DMSO-*d*_*6*_ 125 MHz) δ (ppm): 195.1 (N(C=S)S), 165.8 (C=O), 152.5 (Ar-C), 152.0 (Ar-C), 141.0 (Ar-C), 138.5 (Ar-C), 136.3 (Ar-C), 134.0 (Ar-C), 132.6 (Ar-C), 129.6 (2 × Ar-C), 128.9 (2 × Ar-C), 128.2 (2 × Ar-C), 127.7 (Ar-C), 122.3 (2 × Ar-C), 114.4 (Ar-C), 50.7 (piperazine, NCH_2_), 48.7 (piperazine, NCH_2_), 46.4 (piperazine, 2 × NCH_2_), and 40.3 (SCH_2_). Elemental analysis of C_24_H_22_ClN_5_O_3_S_2_: calculated: C, 54.59; H, 4.20; and N, 13.26; found: C, 54.63; H, 4.17; and N, 13.31%.

#### 3.4.5 4-((3-Bromophenyl)carbamoyl)benzyl 4-(3-nitropyridin-2-yl)piperazine-1-carbodithioate (**7e**)


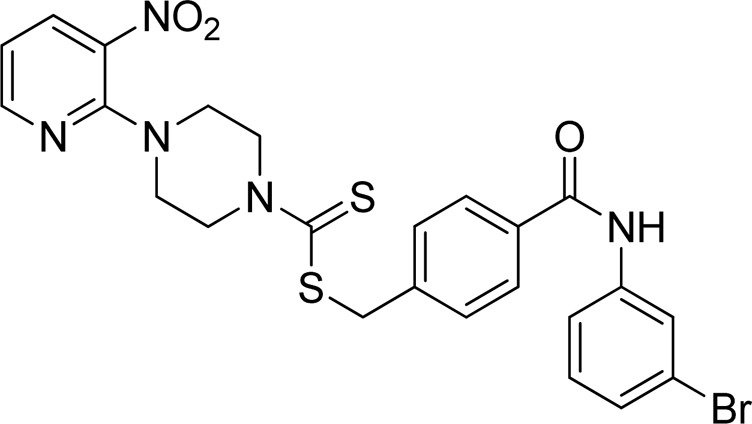


Yield, 55%; yellow solid; mp: 141°C–142°C. ^1^H NMR (DMSO-*d*_*6*_ 500 MHz) δ (ppm): 10.36 (s, 1H, NH), 8.43–8.44 (m, 1H, Ar-H), 8.28 (d, 1H, *J =* 6.65 Hz, Ar-H), 8.08 (s, 1H, Ar-H), 7.88 (d, 2H, *J =* 6.60 Hz, Ar-H), 7.71 (d, 1H, *J =* 6.65 Hz, Ar-H), 7.55 (d, 2H, *J =* 6.60 Hz, Ar-H), 7.27–32 (m, 2H, Ar-H), 6.95 (q, 1H, *J =* 6.75 Hz, Ar-H), 4.66 (s, 2H, methylene), 4.34 (Br s, 2H, piperazinyl), 4.07 (Br s, 2H, piperazinyl), and 3.57 (Br s, 4H, piperazinyl). Elemental analysis of C_24_H_22_BrN_5_O_3_S_2_: calculated: C, 50.35; H, 3.87; and N, 12.23; found: C, 50.39; H, 3.86; and N, 12.28%.

#### 3.4.6 4-((4-Bromophenyl)carbamoyl)benzyl 4-(3-nitropyridin-2-yl)piperazine-1-carbodithioate (**7f**)


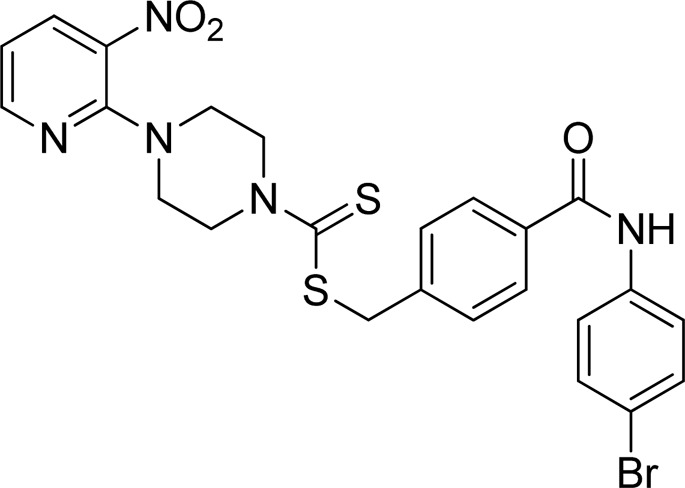


Yield, 59%; yellow solid; mp: 162°C–164°C. ^1^H NMR (DMSO-*d*_*6*_ 500 MHz) δ (ppm): 10.36 (s, 1H, NH), 8.44–8.45 (m, 1H, Ar-H), 8.30 (dd, 1H, *J =* 6.7 Hz, 1.4 Hz, Ar-H), 7.89 (d, 2H, *J =* 6.90 Hz, Ar-H), 7.76 (d, 2H, *J =* 7.4 Hz, Ar-H), 7.52–7.56 (m, 4H, Ar-H), 6.95 (q, 1H, *J =* 6.8 Hz, Ar-H), 4.67 (s, 2H, methylene), 4.34 (Br s, 2H, piperazinyl), 4.08 (Br s, 2H, piperazinyl), and 3.58 (Br s, 4H, piperazinyl). Elemental analysis of C_24_H_22_BrN_5_O_3_S_2_: calculated: C, 50.35; H, 3.87; and N, 12.23; found: C, 50.38; H, 3.91; and N, 12.25%.

#### 3.4.7 4-((2-Nitrophenyl)carbamoyl)benzyl 4-(3-nitropyridin-2-yl)piperazine-1-carbodithioate (**7g**)


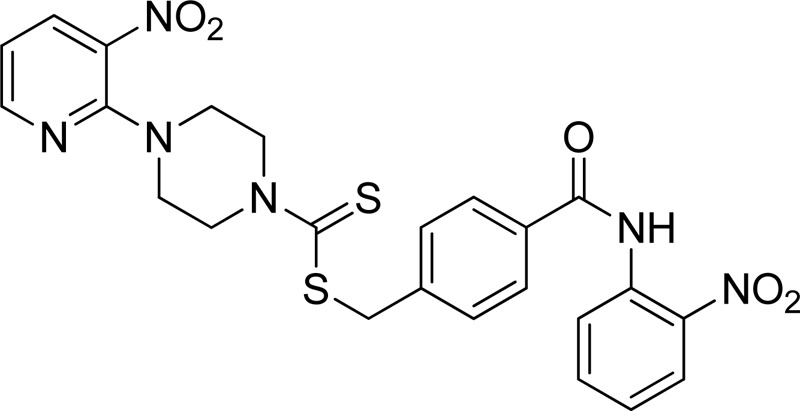


Yield 71%; yellow solid; mp: 133°C–134°C. ^1^H NMR (DMSO-*d*_*6*_ 500 MHz) δ (ppm): 10.78 (s, 1H, NH), 8.44–8.45 (m, 1H, Ar-H), 8.31 (dd, 1H, *J =* 6.65 Hz, 1.35 Hz, Ar-H), 8.28 (d, 2H, *J =* 7.85 Ar-H), 8.07 (d, 2H, *J =* 6.70 Hz, Ar-H), 7.93 (d, 2H, *J =* 6.95 Hz, Ar-H), 7.59 (d, 2H, *J =* 6.8 Hz, Ar-H), 6.98 (q, 1H, *J =* 6.65 Hz, Ar-H), 4.67 (s, 2H, methylene), 4.35 (Br s, 2H, piperazinyl), 4.09 (Br s, 2H, piperazinyl), and 3.59 (Br s, 4H, piperazinyl). Elemental analysis of C_24_H_22_N_6_O_5_S_2_: calculated: C, 53.52; H, 4.12; and N, 15.60; found: C, 53.55; H, 4.15; and N, 15.55%.

#### 3.4.8 4-((3-Nitrophenyl)carbamoyl)benzyl 4-(3-nitropyridin-2-yl)piperazine-1-carbodithioate (**7h**)


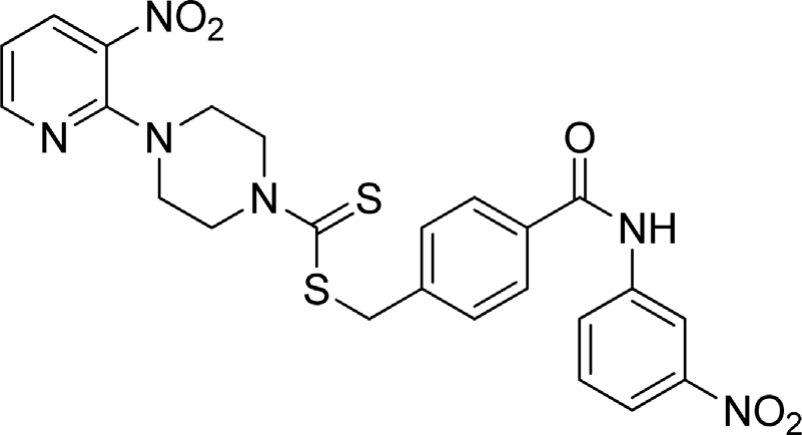


Yield, 49%; yellow solid; mp: 165°C–166°C. ^1^H NMR (DMSO-*d*_*6*_ 500 MHz) δ (ppm): 10.66 (s, 1H, NH), 8.80 (s, 1H, Ar-H), 8.44–8.45 (m, 1H, Ar-H), 8.29–8.31 (dd, 1H, *J =* 6.7 Hz, 1.40 Hz, Ar-H), 8.18 (d, 1H, *J =* 6.90 Hz, Ar-H), 7.94–7.97 (m, 3H, Ar-H), 7.65 (t, 1H, *J =* 6.80 Hz, Ar-H), 7.59 (d, 2H, *J =* 6.75 Hz, Ar-H), 6.95 (q, 1H, *J =* 6.70 Hz, Ar-H), 4.68 (s, 2H, methylene), 4.35 (Br s, 2H, piperazinyl), 4.09 (Br s, 2H, piperazinyl), and 3.59 (Br s, 4H, piperazinyl). Elemental analysis of C_24_H_22_N_6_O_5_S_2_: calculated: C, 53.52; H, 4.12; and N, 15.60; found: C, 53.57; H, 4.16; and N, 15.56%.

#### 3.4.9 4-((4-Nitrophenyl)carbamoyl)benzyl 4-(3-nitropyridin-2-yl)piperazine-1-carbodithioate (**7i**)


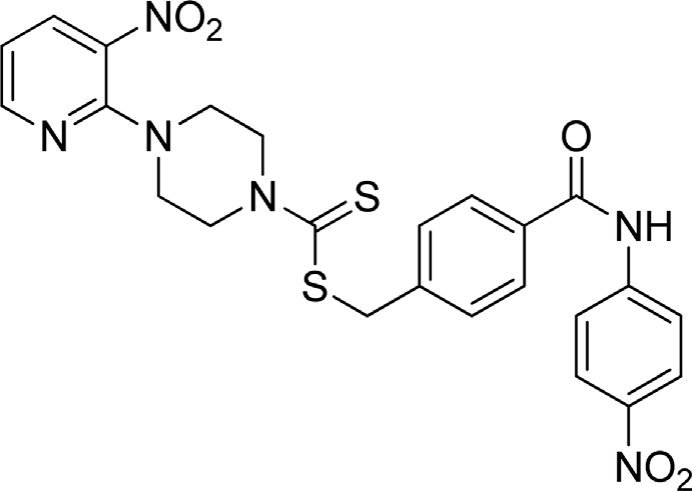


Yield, 63%; yellow solid; mp: 191°C–192°C. ^1^H NMR (DMSO-*d*_*6*_ 500 MHz) δ (ppm): 10.77 (s, 1H, NH), 8.44–8.45 (m, 1H, Ar-H), 8.29 (dd, 1H, *J =* 6.70 Hz, 1.40 Hz, Ar-H), 8.25 (d, 2H, *J =* 7.70 Ar-H), 8.04 (d, 2H, *J =* 6.85 Hz, Ar-H), 7.92 (d, 2H, *J =* 7 Hz, Ar-H), 7.58 (d, 2H, *J =* 6.70 Hz, Ar-H), 6.95 (q, 1H, *J =* 6.70 Hz, Ar-H), 4.68 (s, 2H, methylene), 4.34 (Br s, 2H, piperazinyl), 4.08 (Br s, 2H, piperazinyl), and 3.58 (Br s, 4H, piperazinyl). Elemental analysis of C_24_H_22_N_6_O_5_S_2_: calculated: C, 53.52; H, 4.12; and N, 15.60; found: C, 53.55; H, 4.15; and N, 15.55%.

#### 3.4.10 4-(o-Tolylcarbamoyl)benzyl 4-(3-nitropyridin-2-yl)piperazine-1-carbodithioate (**7j**)


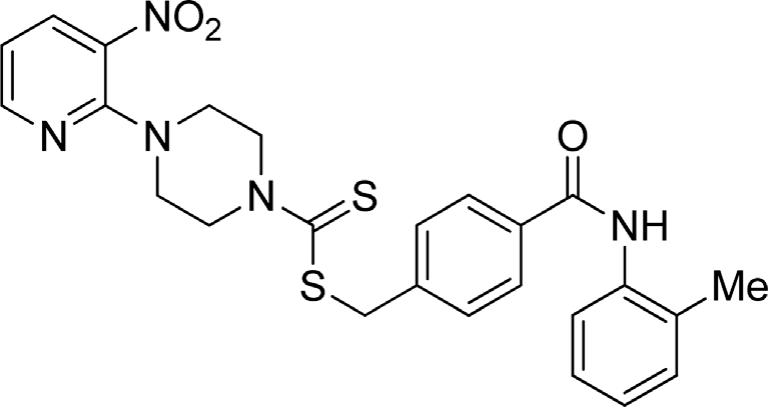


Yield, 55%; yellow solid; mp: 113°C–115°C. ^1^H NMR (DMSO-*d*_*6*_ 500 MHz) δ (ppm): 10.09 (s, 1H, NH), 8.44–8.45 (m, 1H, Ar-H), 8.30 (dd, 1H, *J =* 6.75 Hz, 1.40 Hz, Ar-H), 7.92 (d, 2H, *J =* 6.85 Hz, Ar-H), 7.55 (d, 2H, *J =* 6.90 Hz, Ar-H), 7.33 (d, 1H, *J =* 6.5 Hz, Ar-H), 7.26 (d, 1H, *J =* 6.35 Hz, Ar-H), 7.21 (t, 1H, *J =* 6.2 Hz, Ar-H), 7.16 (t, 1H, *J =* 6.2 Hz, Ar-H), 6.95 (q, 1H, *J =* 6.70 Hz, Ar-H), 4.67 (s, 2H, methylene), 4.34 (Br s, 2H, piperazinyl), 4.08 (Br s, 2H, piperazinyl), 3.58 (Br s, 4H, piperazinyl), and 2.29 (s, 3H, methyl). ^13^C NMR (DMSO-*d*_*6*_ 125 MHz) δ (ppm): 195.1 (N(C=S)S), 165.6 (C=O), 152.6 (Ar-C), 152.0 (Ar-C), 140.7 (Ar-C), 136.8 (Ar-C), 136.3 (Ar-C), 134.1 (Ar-C), 133.9 (Ar-C), 132.6 (Ar-C), 130.7 (Ar-C), 129.6 (2 × Ar-C), 128.2 (2 × Ar-C), 127.4 (Ar-C), 126.4 (2 × Ar-C), 114.4 (Ar-C), 50.7, 49 (piperazine, 2 × NCH_2_), 46.4 (piperazine, 2 × NCH_2_), 40.4 (SCH_2_), and 18.3 (CH_3_). Elemental analysis of C_25_H_25_N_5_O_3_S_2_: calculated: C, 59.15; H, 4.96; and N, 13.80; found: C, 59.19; H, 4.98; and N, 13.77%.

#### 3.4.11 4-(m-Tolylcarbamoyl)benzyl 4-(3-nitropyridin-2-yl)piperazine-1-carbodithioate (**7k**)


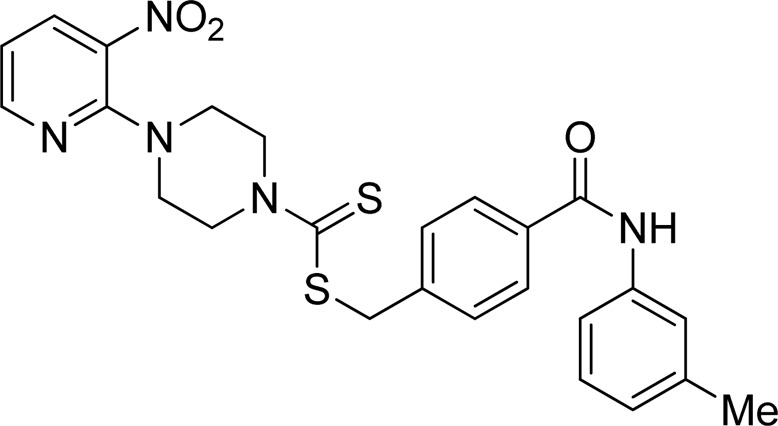


Yield, 57%; yellow solid; mp: 103°C–104°C. ^1^H NMR (DMSO-*d*_*6*_ 500 MHz) δ (ppm): 10.13 (s, 1H, NH), 8.45–8.46 (m, 1H, Ar-H), 8.30 (dd, 1H, *J =* 6.65 Hz, 1.40 Hz, Ar-H), 7.88 (d, 2H, *J =* 6.85 Hz, Ar-H), 7.60 (s, 1H, Ar-H), 7.55 (t, 3H, *J =* 6.90 Hz, Ar-H), 7.22 (t, 1H, *J =* 6.5 Hz, Ar-H), 6.96 (q, 1H, *J =* 6.40 Hz, Ar-H), 6.93 (d, 1H, *J =* 6.30 Hz, Ar-H), 4.67 (Br s, 2H, methylene), 4.35 (Br s, 2H, piperazinyl), 4.08 (Br s, 2H, piperazinyl), 3.58 (Br s, 4H, piperazinyl), and 2.3 (s, 3H, methyl). Elemental analysis of C_25_H_25_N_5_O_3_S_2_: calculated: C, 59.15; H, 4.96; and N, 13.80; found: C, 59.13; H, 4.99; and N, 13.85%.

#### 3.4.12 4-(P-tolylcarbamoyl)benzyl 4-(3-nitropyridin-2-yl)piperazine-1-carbodithioate (**7l**)


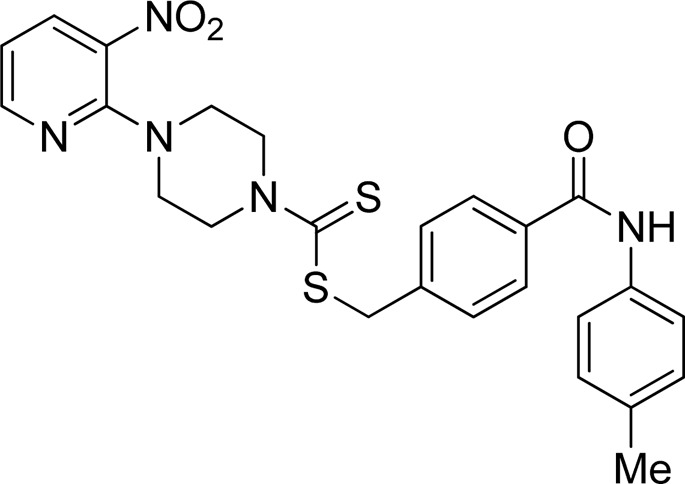


Yield, 51%; yellow solid; mp: 158°C–160°C. ^1^H NMR (DMSO-*d*_*6*_ 500 MHz) δ (ppm): 10.13 (s, 1H, NH), 8.44–8.45 (m, 1H, Ar-H), 8.30 (dd, 1H, *J =* 6.75 Hz, 1.40 Hz, Ar-H), 7.88 (d, 2H, *J =* 7.00 Ar-H), 7.63 (d, 2H, *J =* 7.00 Hz, Ar-H), 7.54 (d, 2H, *J =* 6.95 Hz, Ar-H), 7.15 (d, 2H, *J =* 7.05 Hz, Ar-H), 6.95 (q, 1H, *J =* 6.70, Ar-H), 4.69 (s, 2H, methylene), 4.34 (Br s, 2H, piperazinyl), 4.08 (Br s, 2H, piperazinyl), 3.58 (Br s, 4H, piperazinyl), and 2.28 (s, 3H, methyl). Elemental analysis of C_25_H_25_N_5_O_3_S_2_: calculated: C, 59.15; H, 4.96; and N, 13.80; found: C, 59.20; H, 4.98; and N, 13.75%.

#### 3.4.13 4-((2-Methoxyphenyl)carbamoyl)benzyl 4-(3-nitropyridin-2-yl)piperazine-1-carbodithioate (**7m**)


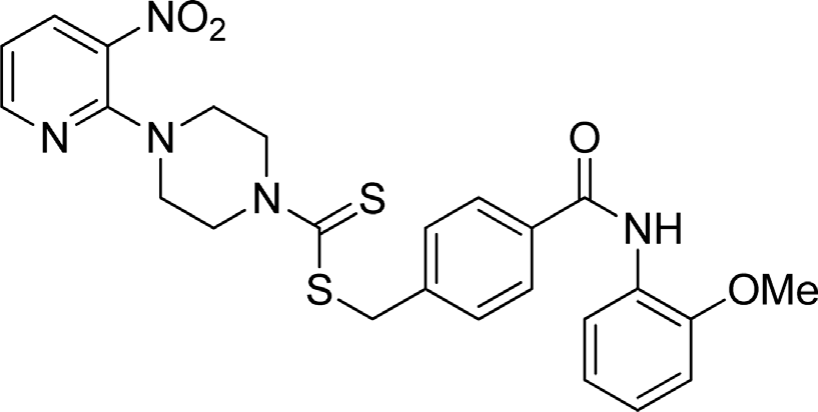


Yield, 67%; yellow solid; mp: 166°C–167°C. ^1^H NMR (DMSO-*d*_*6*_ 500 MHz) δ (ppm): 9.37 (s, 1H, NH), 8.44–8.45 (m, 1H, Ar-H), 8.29–8.30 (dd, 1H, *J =* 6.40 Hz, 1.45 Hz, Ar-H), 7.90 (d, 2H, *J =* 6.85 Hz, Ar-H), 7.79 (dd, 1H, *J =* 6.60 Hz, 1.40 Hz, Ar-H), 7.54 (d, 2H, *J =* 6.9 Hz, Ar-H), 7.16–7.19 (m, 1H, Ar-H), 7.09 (d, 1H, *J =* 7.00 Hz, Ar-H), 6.94–6.98 (m, 2H, Ar-H), 4.67 (s, 2H, methylene), 4.35 (Br s, 2H, piperazinyl), 4.08 (Br s, 2H, piperazinyl), 3.83 (s, 3H, methyl), and 3.58 (Br s, 4H, piperazinyl). Elemental analysis of C_25_H_25_N_5_O_4_S_2_: calculated: C, 57.34; H, 4.81; and N, 13.30; found: C, 57.3; H, 4.84; and N, 13.32%.

#### 3.4.14 4-((4-Methoxyphenyl)carbamoyl)benzyl 4-(3-nitropyridin-2-yl)piperazine-1-carbodithioate (**7n**)


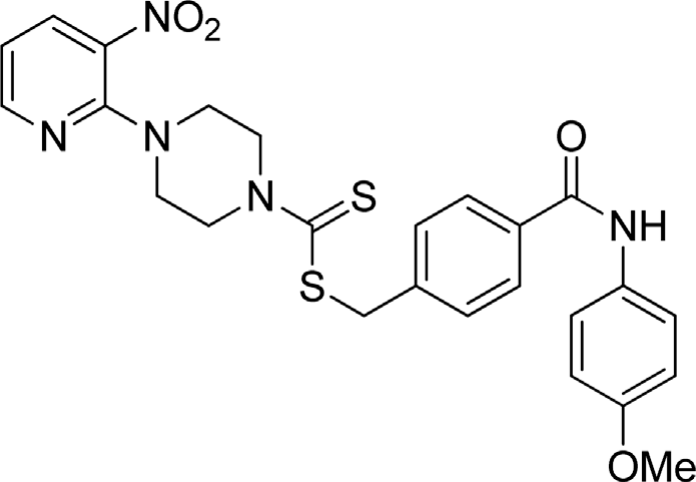


Yield, 51%; yellow solid; mp: 187°C–189°C. ^1^H NMR (DMSO-*d*_*6*_ 500 MHz) δ (ppm): 10.08 (s, 1H, NH), 8.44–8.45 (m, 1H, Ar-H), 8.30 (dd, 1H, *J =* 6.7 Hz, 1.35 Hz, Ar-H), 7.88 (d, 2H, *J =* 6.8 Hz, Ar-H), 7.65 (d, 2H, *J =* 7.5 Hz, Ar-H), 7.54 (d, 2H, *J =* 7.00 Hz, Ar-H), 6.95 (q, 1H, *J =* 6.75 Hz, Ar-H), 6.91 (d, 2H, *J =* 7.5 Hz, Ar-H), 4.66 (s, 2H, methylene), 4.34 (Br s, 2H, piperazinyl), 4.08 (Br s, 2H, piperazinyl), 3.74 (s, 3H, methyl), and 3.58 (Br s, 4H, piperazinyl). ^13^C NMR (DMSO-*d*_*6*_ 125 MHz) δ (ppm): 195.1 (N(C=S)S), 165.4 (C=O), 156.0 (Ar-C), 152.6 (Ar-C), 152.0 (Ar-C), 140.5 (Ar-C), 136.3 (Ar-C), 134.4 (Ar-C), 132.2 (2 × Ar-C), 129.5 (2 × Ar-C), 128.1 (2 × Ar-C), 122.4 (2 × Ar-C), 114.4 (Ar-C), 114.2 (Ar-C), 55.6 (OCH_3_), 50.6 (piperazine, NCH_2_), 49.8 (piperazine, NCH_2_), 46.4 (piperazine, 2 × NCH_2_), and 40.4 (SCH_2_). Elemental analysis of C_25_H_25_N_5_O_4_S_2_: calculated: C, 57.34; H, 4.81; and N, 13.30; found: C, 57.38; H, 4.76; and N, 13.34%.

### 3.5 Urease inhibition activity assay

The inhibitory potential of the compounds was assessed using a slightly modified indophenol method (Hina et al., 2023). In 96-well plates, a mixture consisting of 50 µL urease, 30 µL buffer solution (phosphate buffer, sodium salicylate, sodium nitroprusside, and EDTA, pH 8.0), 10 µL urea substrate (100 mM), and 10 µL of the test compound (1 mM) was subjected to pre-incubation for 10 min at room temperature. Subsequently, an alkali reagent (70 µL) was promptly introduced into each well. Then, 30 min later, the absorbance at 630 nm for all samples was measured using a BioTek ELx800 instrument from BioTek Instruments, Inc., United States. The data were collected for all experiments performed in triplicate, and the percentage of inhibitory activities was assessed utilizing the following formula:

$$\text{Percentage of inhibition}=100\;–\;\left(\text{Absorbance of compound}/\text{Absorbance of control}\right)\;\times \;100.$$

#### 3.5.1 Kinetic study

Experiments using Michaelis–Menten kinetics were conducted to identify the type of enzyme inhibition exhibited by urease. Detailed kinetic analyses were undertaken using the most potent compound **(5j)** to investigate its potential mechanism of action in inhibiting the respective enzyme. The type of inhibitory action was determined by using four different concentrations of the substrate (0, 2.5, 5, 10, and 15 mM) in the absence and presence of different concentrations of inhibitor **5j** (0, 2.55, 5.11, and 7.65 μM).

### 3.6 Protocol for *in silico* studies

#### 3.6.1 Structure selection and preparation

The structure of the ligands was sketched using ChemDraw Ultra 12.0 from the ChemOffice suite, ensuring that they were given accurate 2D orientations. Subsequently, the structure of each compound was scrutinized for any bond-order connection errors. To explore the notable interactions between the inhibitors and the enzyme urease, we utilized docking procedures. We obtained the crystallographic structure of Jack bean urease (3LA4) from the RCSB Protein Data Bank library. Moreover, these structures were prepared for further analysis in docking studies (Cunha et al., 2021). Before conducting docking studies, the compounds and the urease enzyme were prepared in the following manner: to prevent the binding pockets from collapsing during the energy minimization calculations, a slight force was applied to reinforce the backbone atoms. As a result, the ligands and water molecules were eliminated, and polar hydrogens were added to the crystallographic structure (Labute, 2007).

#### 3.6.2 Molecular docking analysis and simulations

Docking studies of inhibitory compound **5j** were performed against the urease enzyme. In order to optimize the effectiveness of the docking results, the urease structure was prepared by eliminating any unwanted ligands and water molecules. The docking analysis for receptor–ligand interactions utilized a grid box with dimensions of 80 × 72 × 66 Å along the *x*-, *y*-, and *z*-axes, respectively. This grid had a grid point spacing of 0.375 Å and was centered at coordinates 7.836 Å in the *x*-axis, 10.509 Å in the *y*-axis, and 22.951 Å in the *z*-axis. The finest binding conformational pose of the protein–ligand docked complexes was obtained by utilizing a default exhaustiveness value of 8. AutoDock Vina software was used to conduct the docking analysis and generate binding affinities. To visualize the residues of amino acids interacting at the active site of the protein, molecular visualization was performed using Edu-PyMOL.

Molecular dynamics simulation (MDS) analysis was used to investigate the stability of the interaction between the protein and the ligand. Furthermore, MD simulation studies were used to scrutinize the structural transitions within the macromolecules, elucidating the functional significance of the resulting complex. In this simulation, atomic movements over time were recorded in accordance with Newton’s fundamental motion equation, providing insights into how the ligand binds within the biological environment (Hassan et al., 2022).

#### 3.6.3 ADMET analysis

The absorption–distribution–metabolism–excretion–toxicity (ADMET) analysis of the potent inhibitor **5j** was performed via admetSAR (http://lmmd.ecust.edu.cn/admetsar2), ProTox-II (https://tox-new.charite.de/protox_II/), and eMolTox (https://xundrug.cn/moltox). All these tools are freely accessible and not only determine the physicochemical properties of a compound but also interpret whether a compound has the ability to be a drug or not. In addition, ProTox-II also predicts the LD_50_ value and toxicity class of the query SMILE, and eMolTox evaluates the toxic substructures existing in the input compound (Cheng et al., 2012; Banerjee et al., 2018; Ji et al., 2018).

## 4 Conclusion

The inhibitory activity against urease was evaluated for the compounds (**5a**–**5n** and **7a**–**7n**). All compounds exhibited notably stronger inhibitory potential than the positive control (thiourea). Notably, **5j** emerged as the primary inhibitor, demonstrating remarkable efficacy with an IC_50_ value of 5.16 ± 2.68 µM. The docking analysis revealed diverse interactions between **5j** and the active site amino acids. The *in silico* ADMET profile exhibited diverse drug-like characteristics of **5j.** The lead inhibitor revealed notable docking scores and effective binding free energies, showing a strong binding interaction.

## Data Availability

The original contributions presented in the study are included in the article/Supplementary Material; further inquiries can be directed to the corresponding authors.
