# Supplementary material for: Pyridylpiperazine-based carbodithioates as urease inhibitors: synthesis and biological evaluation
Source: Front Chem. 2024 Aug 6;12:1423385. doi: 10.3389/fchem.2024.1423385 (PMC11333205; doi:10.3389/fchem.2024.1423385)
Supplement: Supplementary file 1 [file DataSheet1.pdf]

Pyridylpiperazine based carbodithioates as Urease Inhibitors: Synthesis and biological evaluation

Muhammad Akash[1], Nehal Rana[2], Sana Aslam[3], Matloob Ahmad[1,*], Aneeza Asghar[2], Sadia Sultan[4,5], Afifa Liaqat[2], Sami A. Al-Hussain[6], Sumera Zaib[2,*], Magdi E. A. Zaki[6,*]

[2]Department of Basic and Applied Chemistry, Faculty of Science and Technology, University of Central Punjab, Lahore 54590, Pakistan

*** Correspondence:**
*** Correspondence:**
Sana Aslam, dr.sana@gcwuf.edu.pk.

Matloob Ahmad, Matloob.Ahmad@gcuf.edu.pk

Sumera Zaib, sumera.zaib@ucp.edu.pk

Magdi E. A. Zaki, mezaki@imamu.edu.sa

Supplementary Material

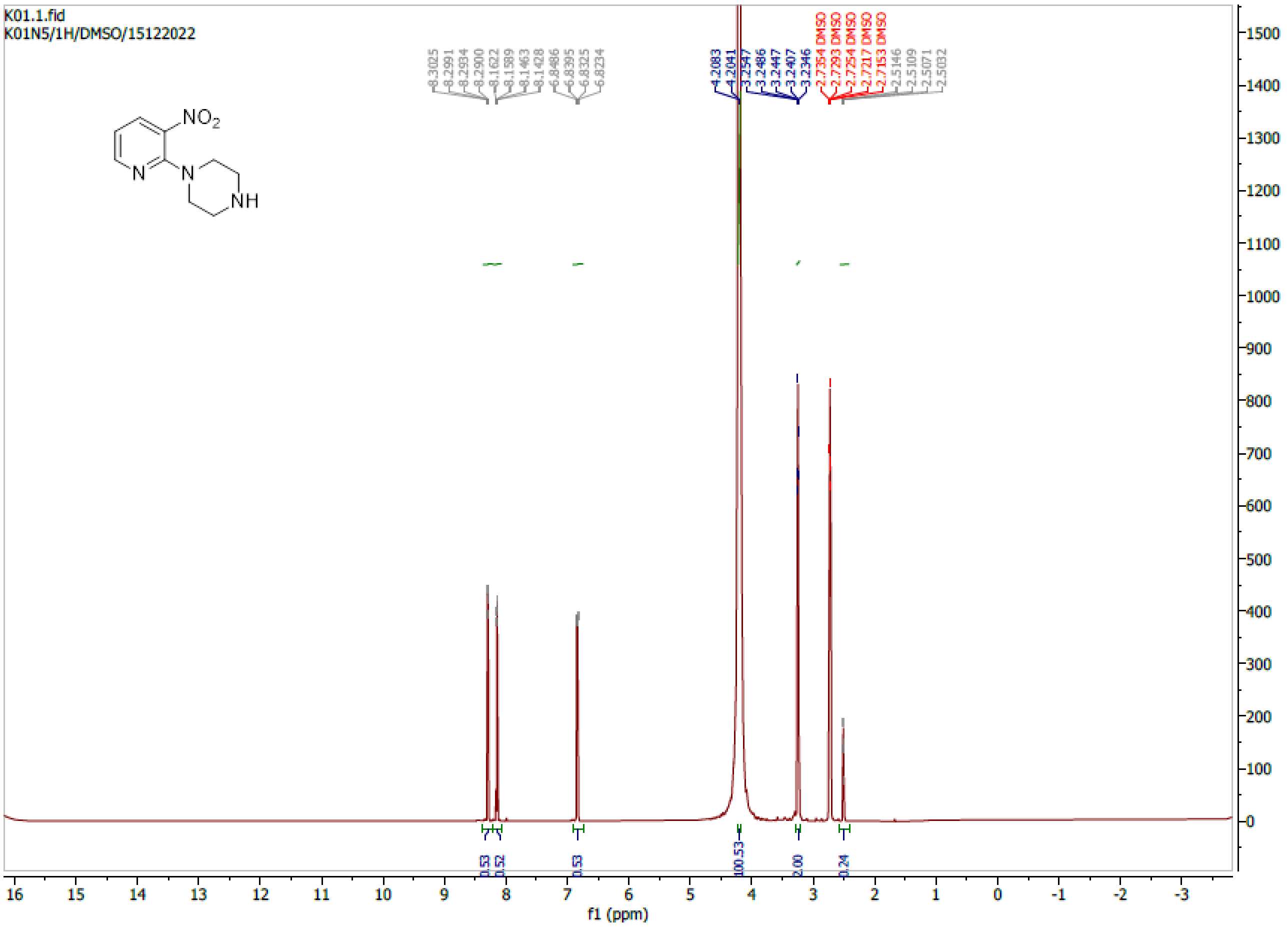

Figure S1: ^{1}HNMR of 1-(3-nitropyridin-2-yl)piperazine **(3)**

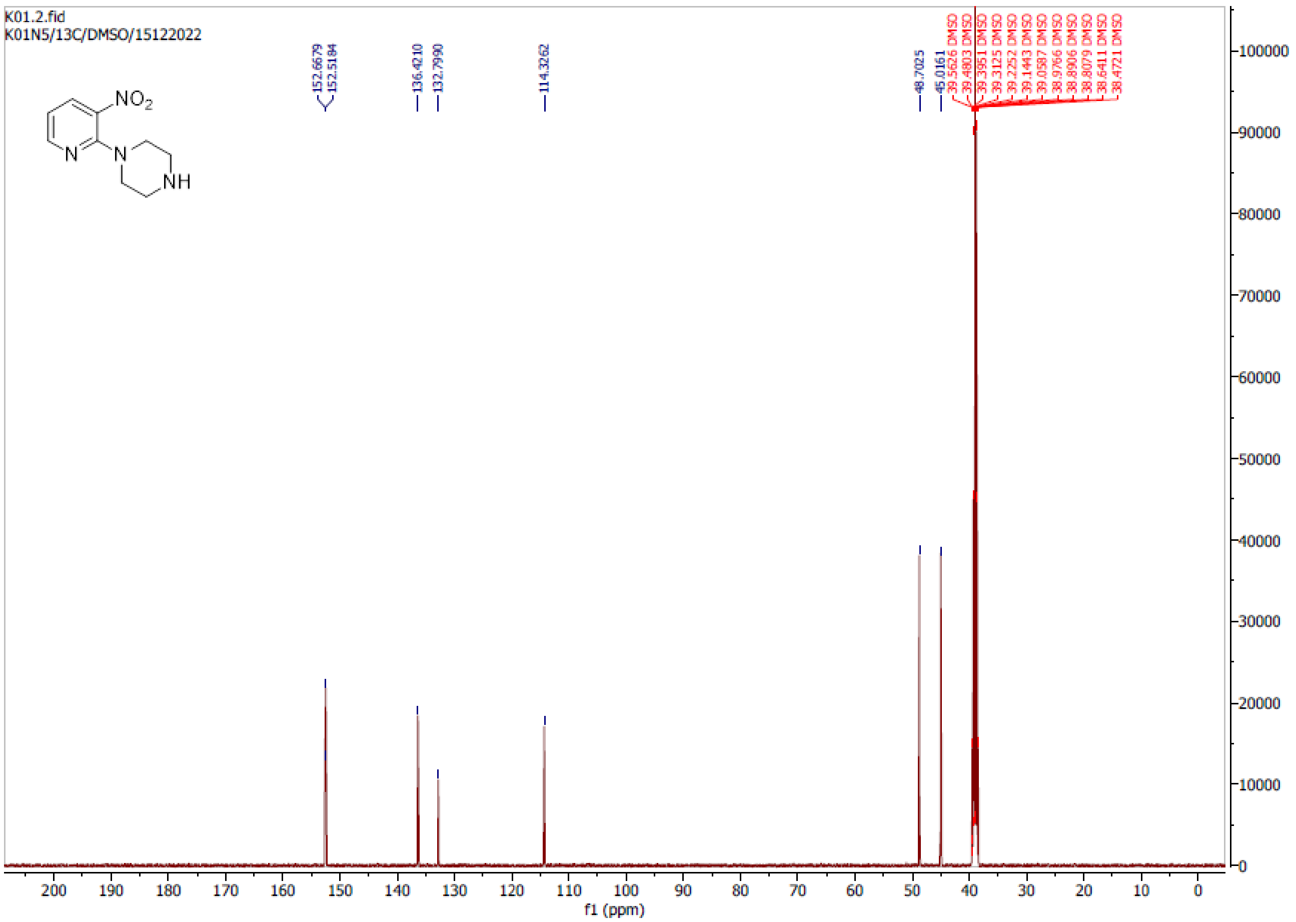

Figure S2: ^{13}CNMR of 1-(3-nitropyridin-2-yl)piperazine **(3)**

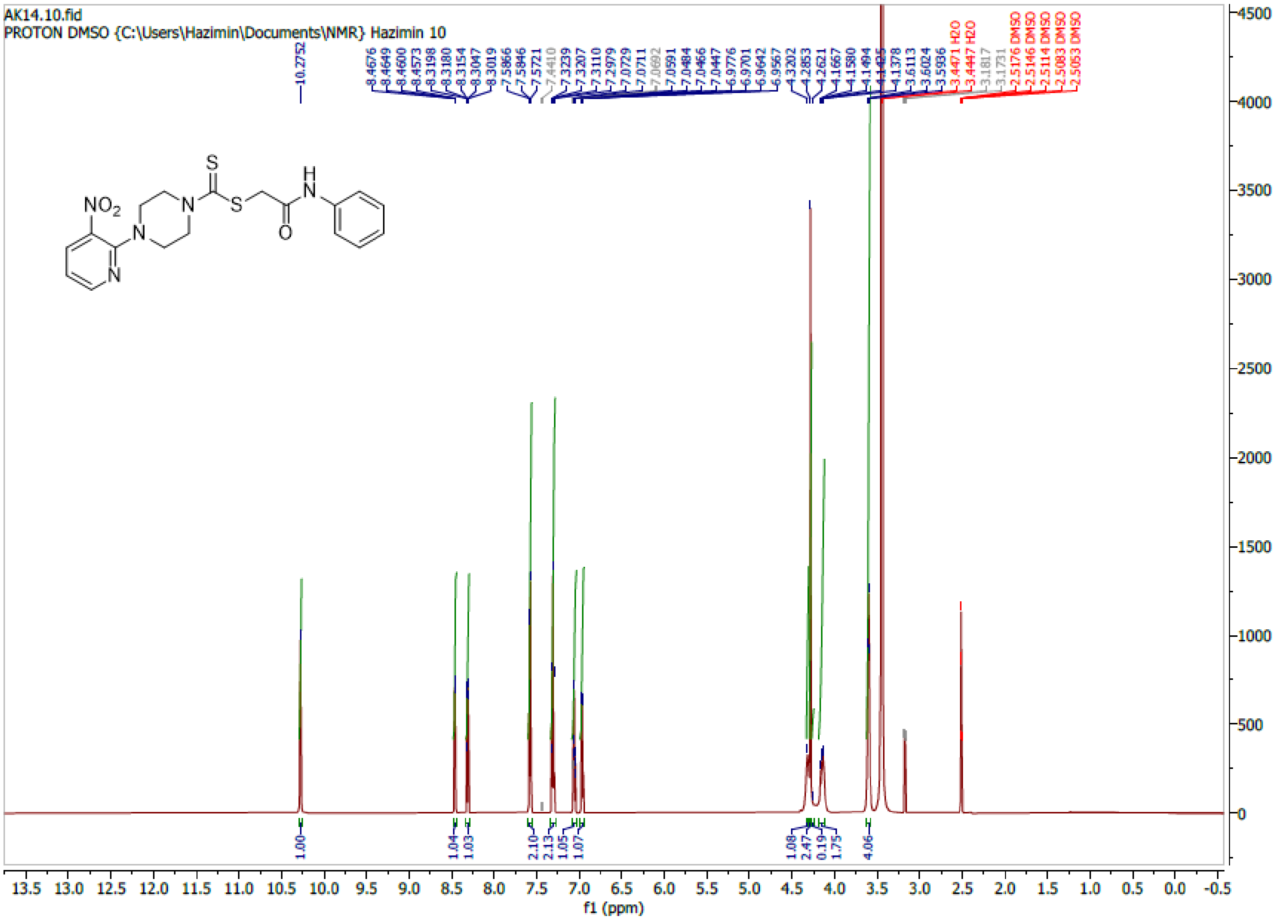
AK14.10.fid
PROTON DMSO {C:\Users\Hazimin\Documents\NMR} Hazimin 10
f1 (ppm)

Figure S3: ^{1}HNMR of 2-oxo-2-(phenylamino)ethyl 4-(3-nitropyridin-2-yl)piperazine-1-carbodithioate **(5a)**

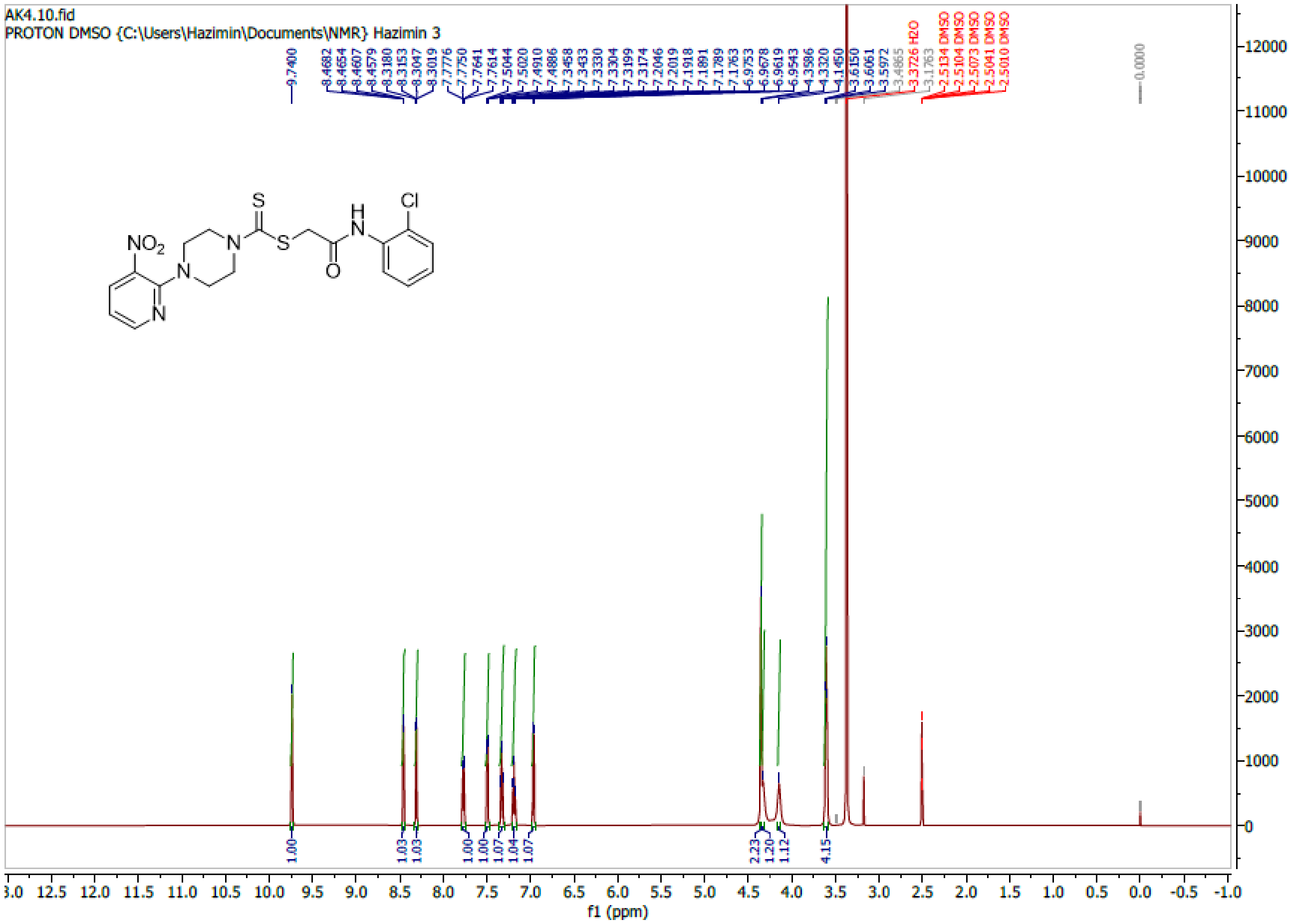

Figure S4: ^{1}HNMR of 2-((2-chlorophenyl)amino)-2-oxoethyl 4-(3-nitropyridin-2-yl)piperazine-1-carbodithioate **(5b)**

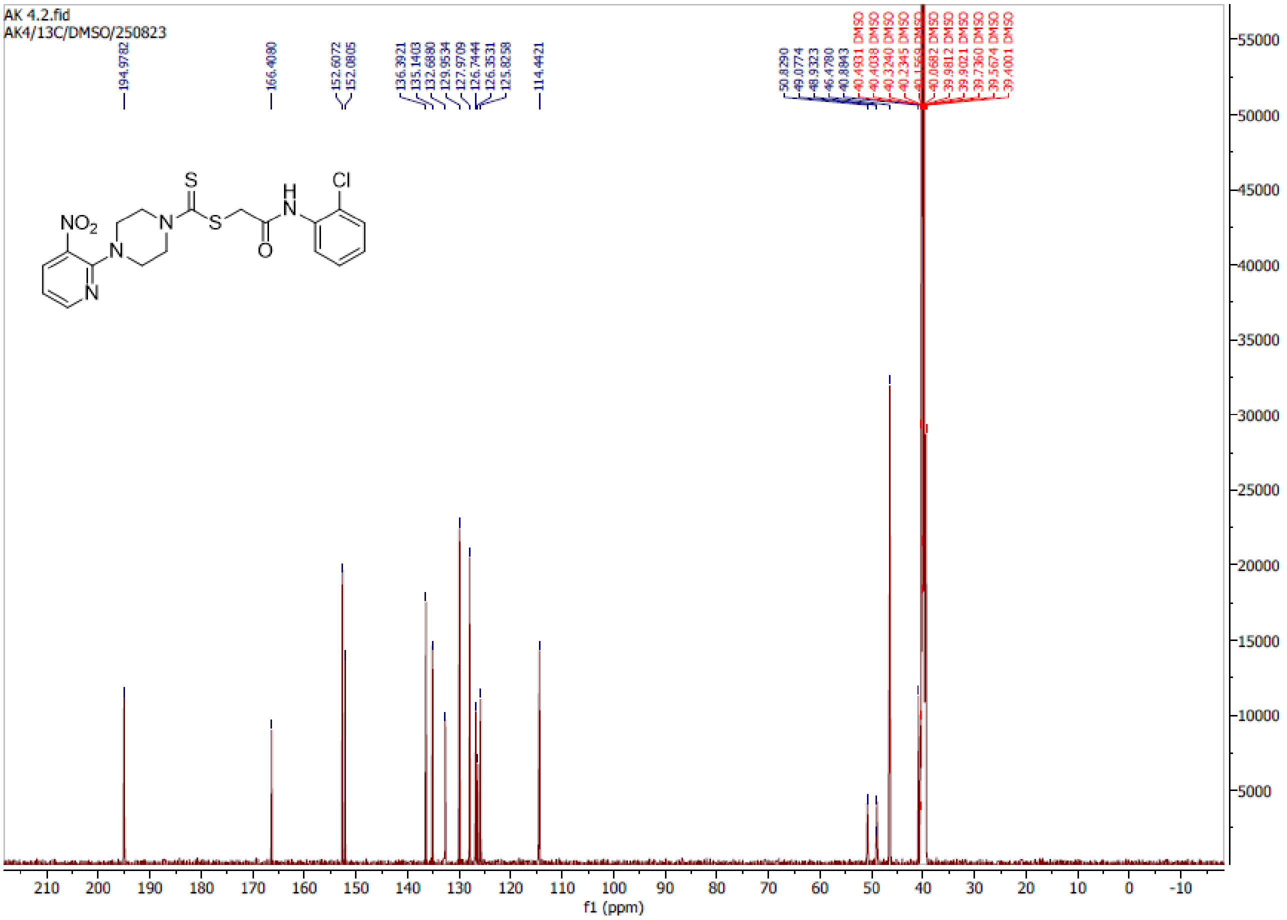

Figure S5: ^{13}CNMR of 2-((2-chlorophenyl)amino)-2-oxoethyl 4-(3-nitropyridin-2-yl)piperazine-1-carbodithioate **(5b)**

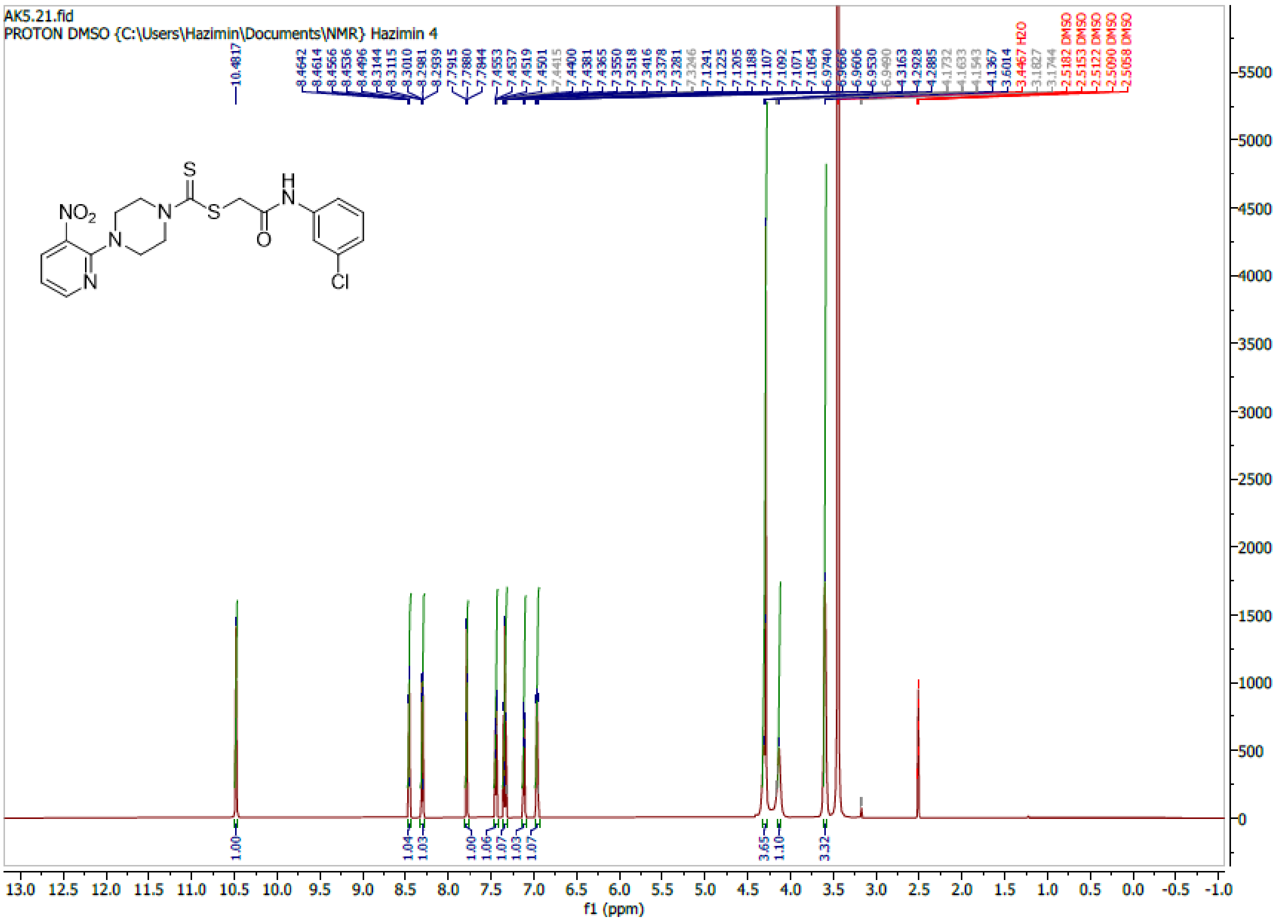

Figure S6: ^{1}HNMR of 2-((3-chlorophenyl)amino)-2-oxoethyl 4-(3-nitropyridin-2-yl)piperazine-1-carbodithioate **(5c)**

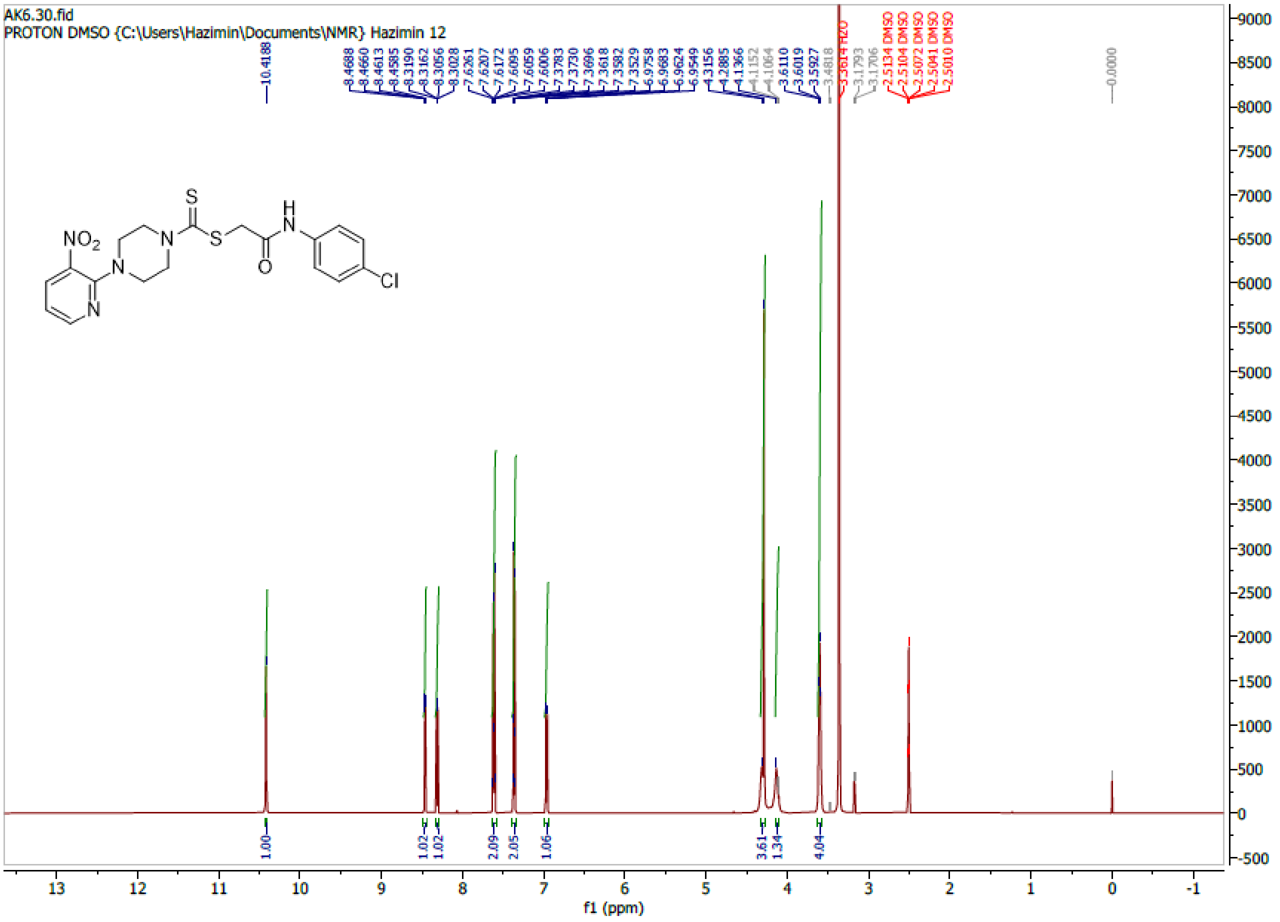

Figure S7: ^{1}HNMR of 2-((4-chlorophenyl)amino)-2-oxoethyl 4-(3-nitropyridin-2-yl)piperazine-1-carbodithioate **(5d)**

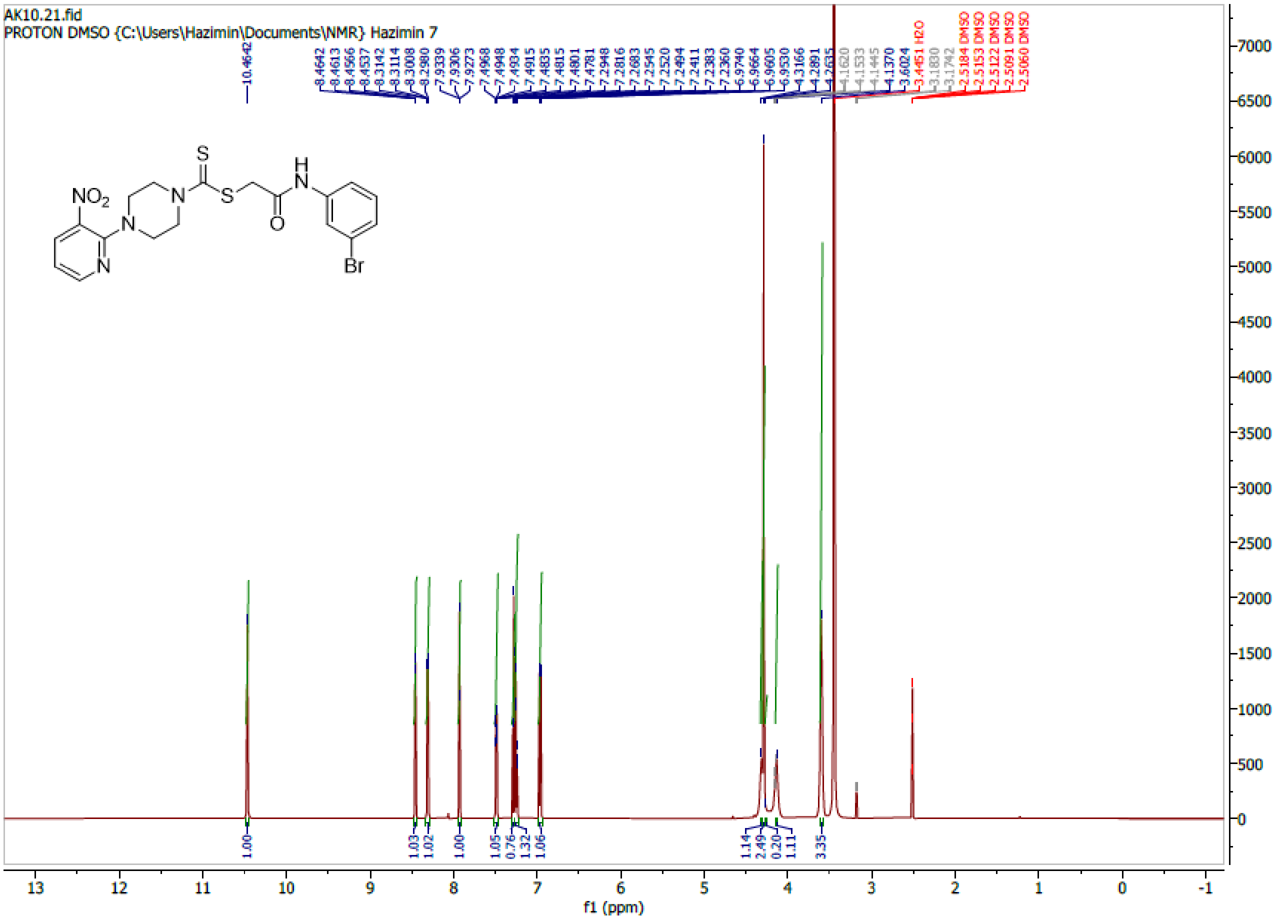

Figure S8: ^{1}HNMR of 2-((3-bromophenyl)amino)-2-oxoethyl 4-(3-nitropyridin-2-yl)piperazine-1-carbodithioate **(5e)**

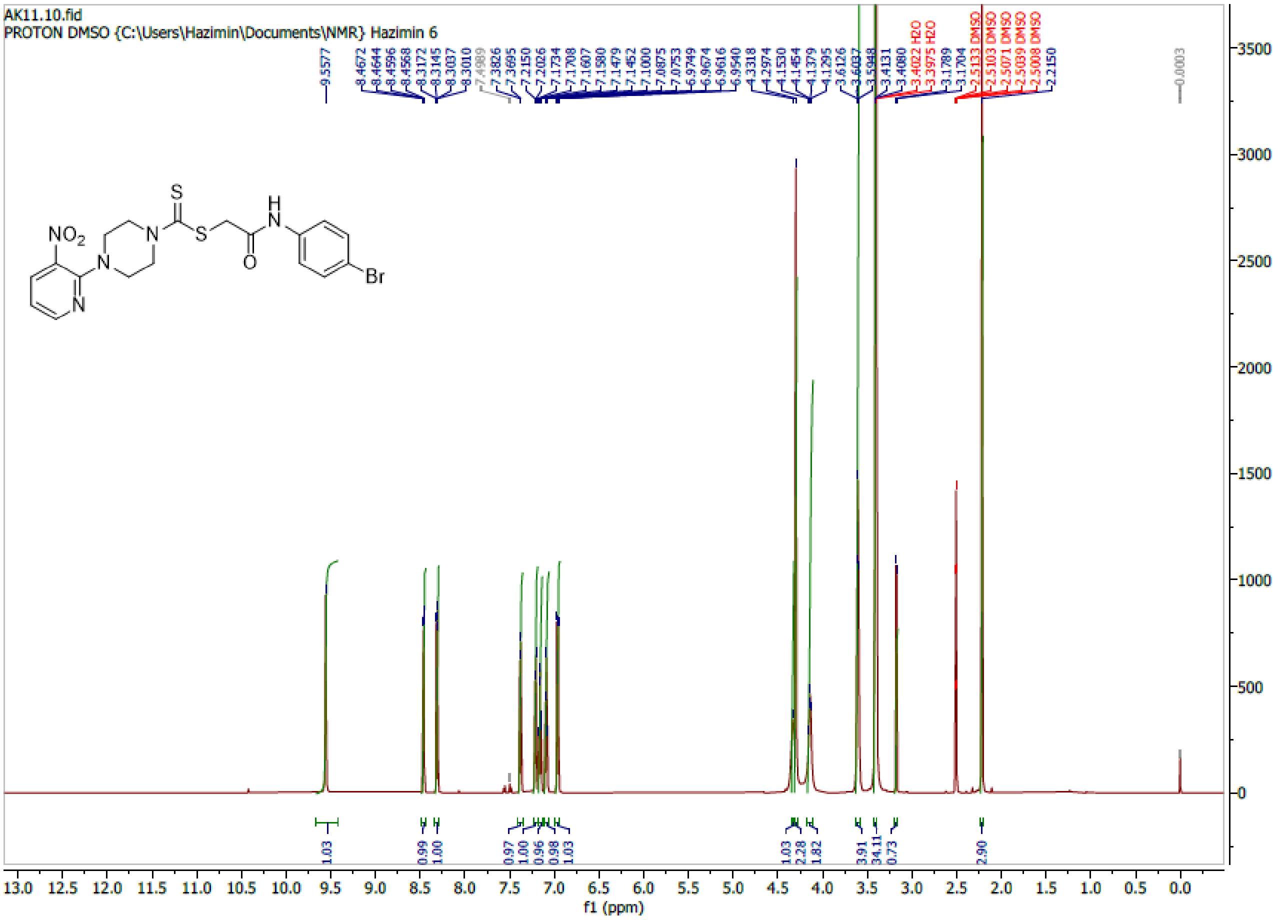

AK11.10.fid
PROTON DMSO {C:\Users\Hazimin\Documents\NMR} Hazimin 6
f1 (ppm)

Figure S9: ^{1}HNMR of 2-((4-bromophenyl)amino)-2-oxoethyl 4-(3-nitropyridin-2-yl)piperazine-1-carbodithioate **(5f)**

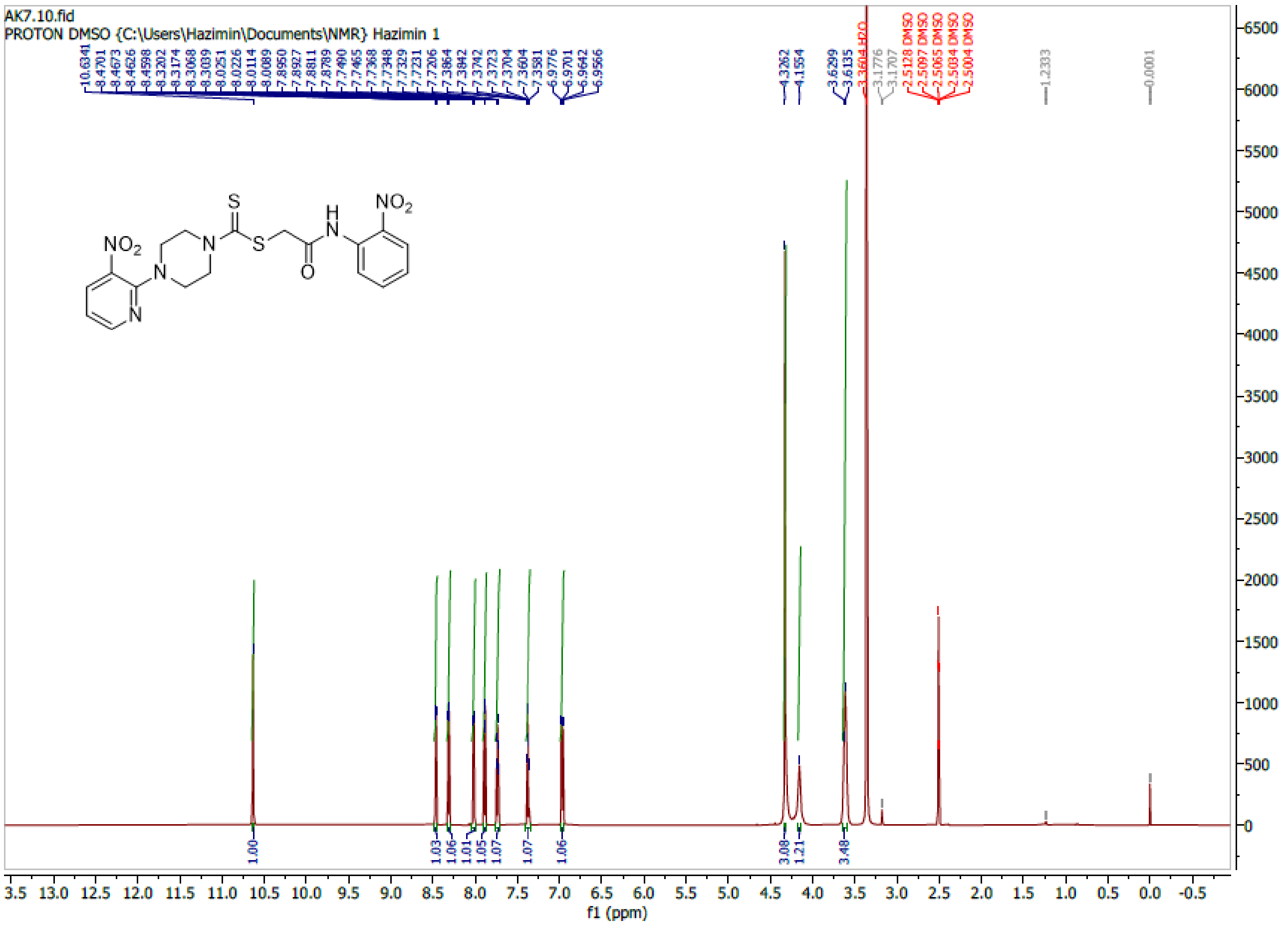

Figure S10: ^{1}HNMR of 2-((2-nitrophenyl)amino)-2-oxoethyl 4-(3-nitropyridin-2-yl)piperazine-1-carbodithioate **(5g)**

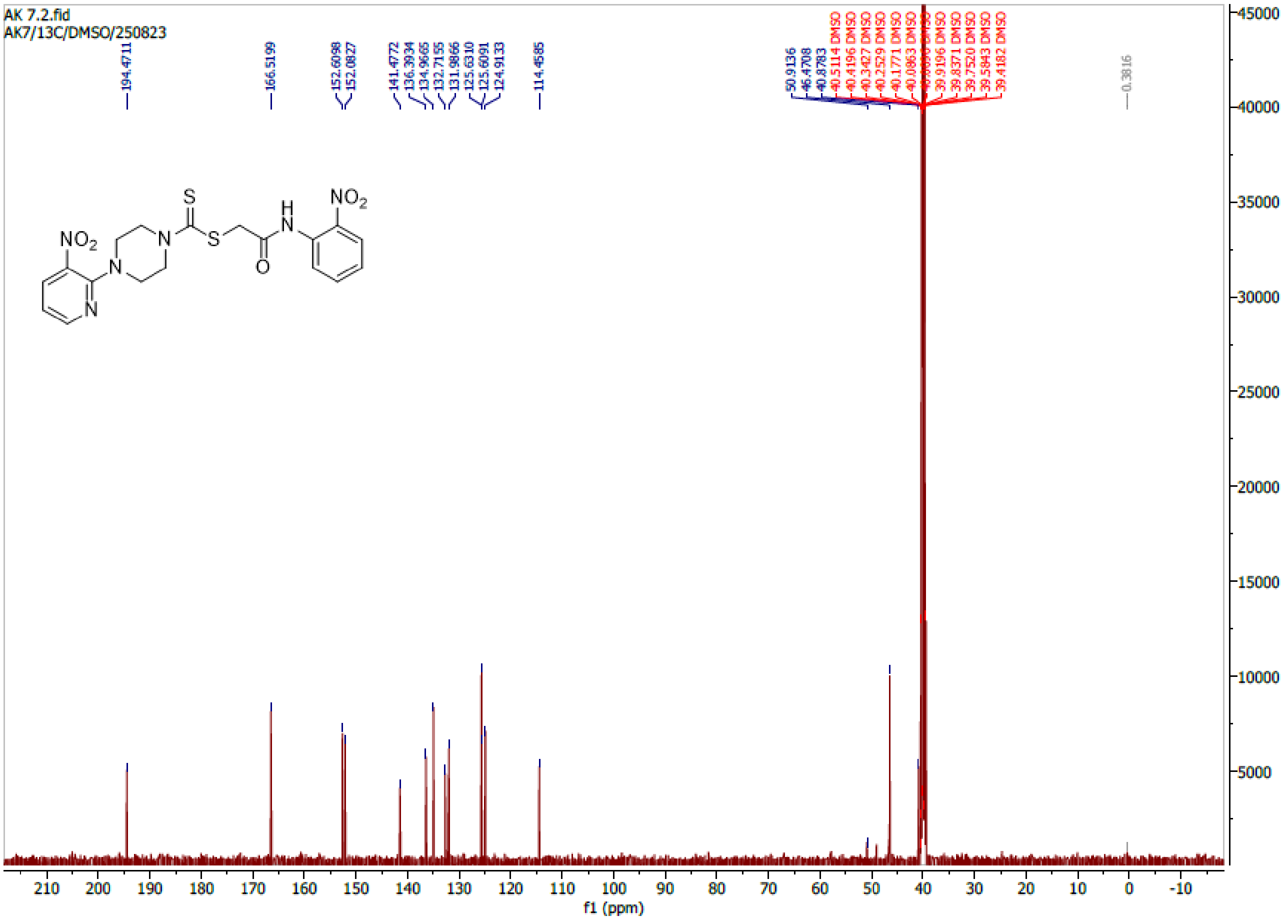

Figure S11: ^{13}CNMR of 2-((2-nitrophenyl)amino)-2-oxoethyl 4-(3-nitropyridin-2-yl)piperazine-1-carbodithioate **(5g)**

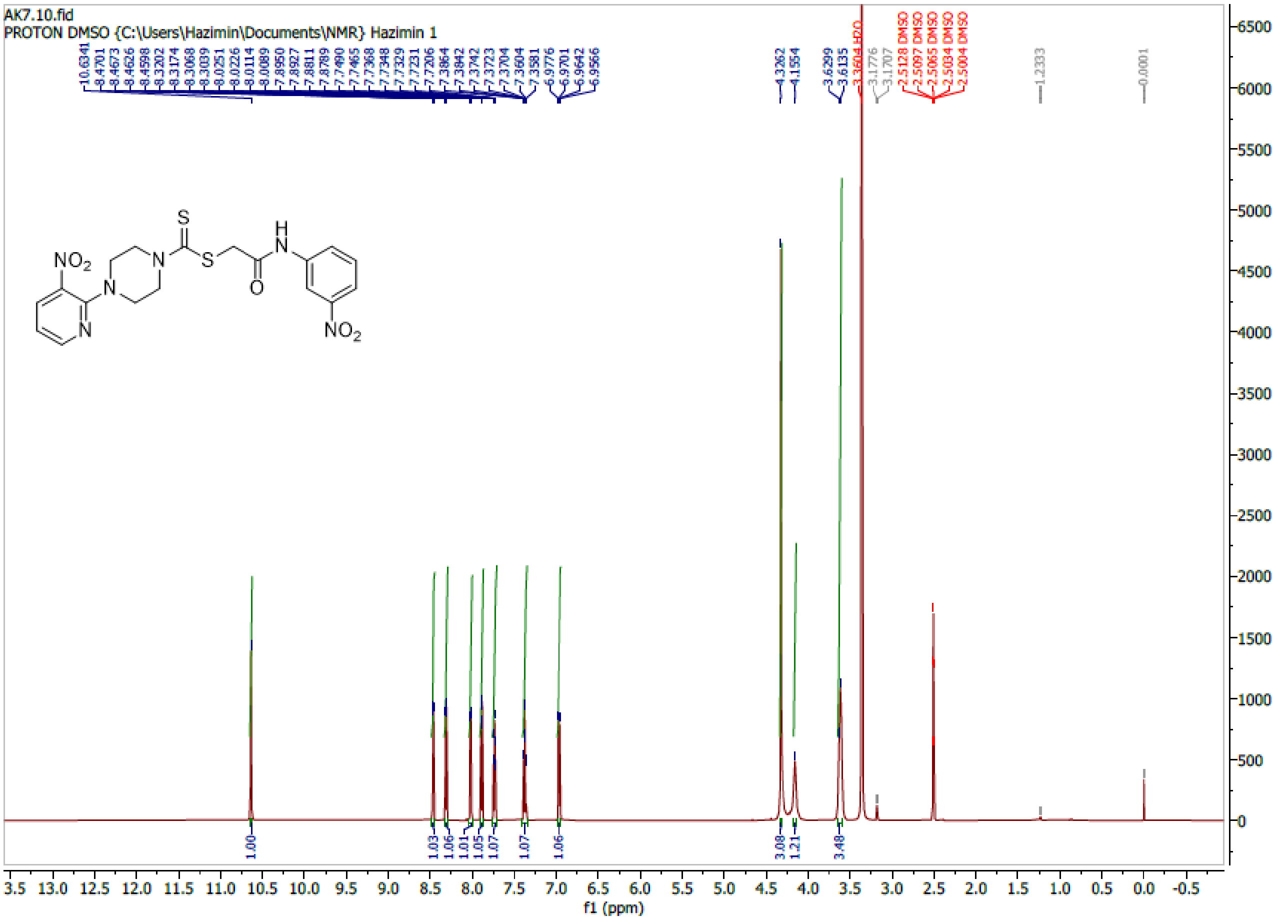

Figure S12: ^{1}HNMR of 2-((3-nitrophenyl)amino)-2-oxoethyl 4-(3-nitropyridin-2-yl)piperazine-1-carbodithioate **(5h)**

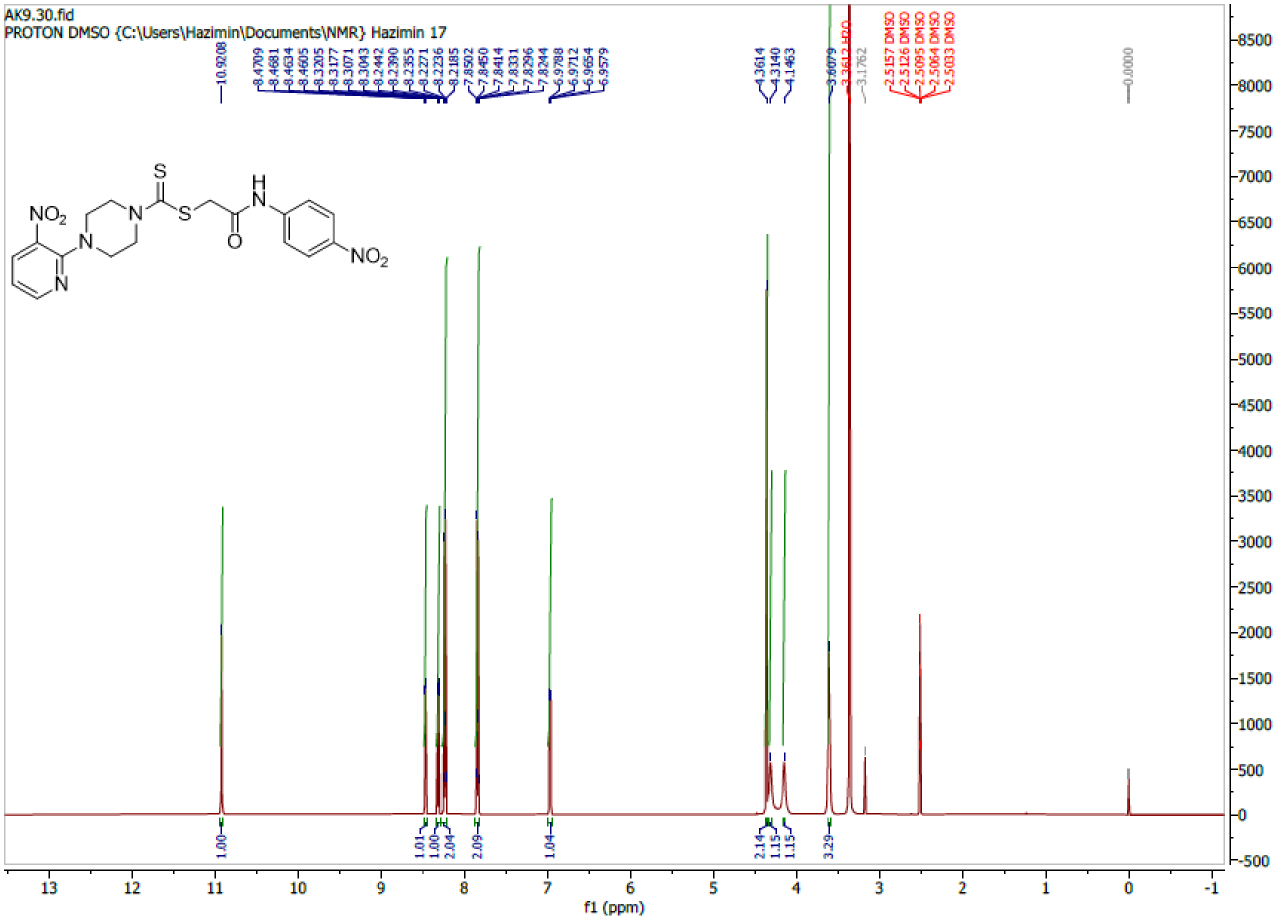

Figure S13: ^{1}HNMR of 2-((4-nitrophenyl)amino)-2-oxoethyl 4-(3-nitropyridin-2-yl)piperazine-1-carbodithioate **(5i)**

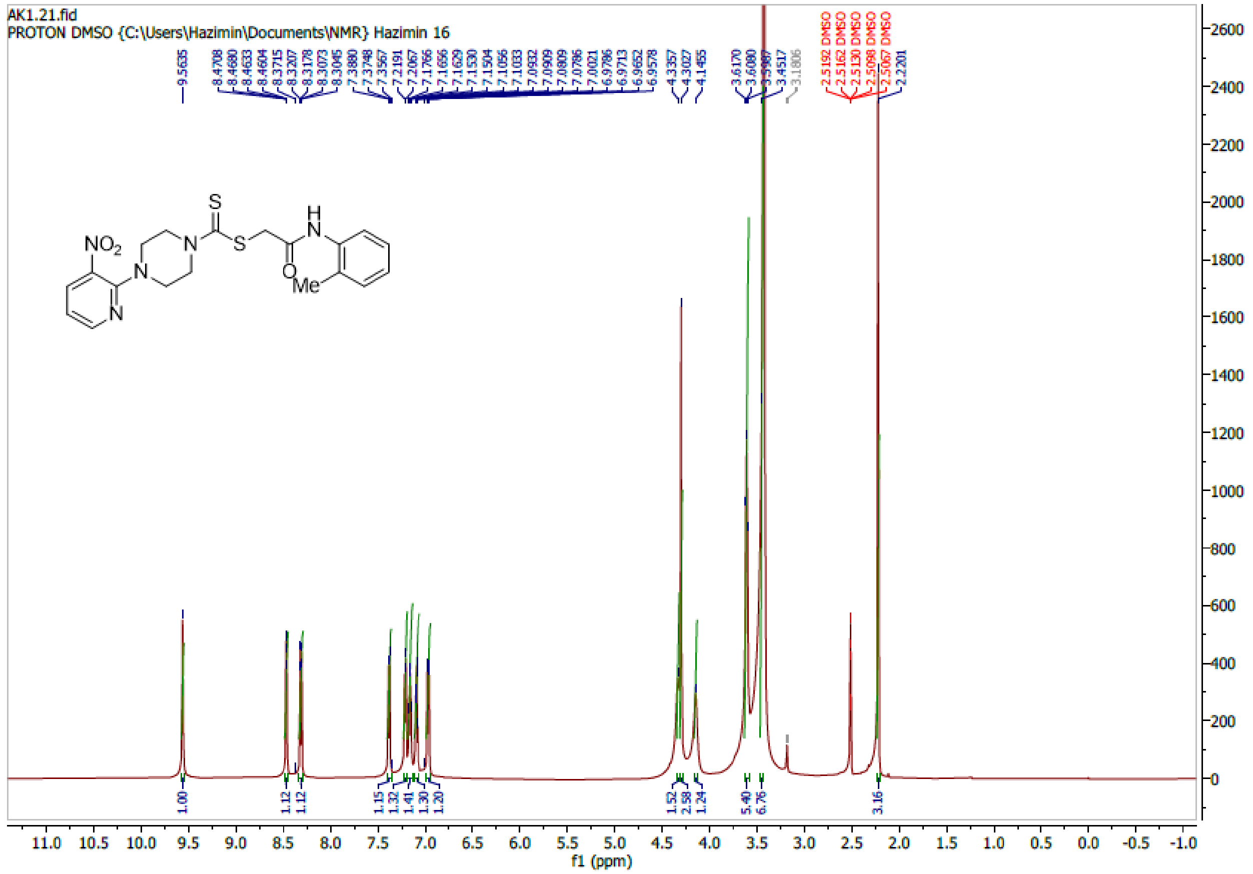

Figure S14: ^{1}HNMR of 2-oxo-2-(o-tolylamino)ethyl 4-(3-nitropyridin-2-yl)piperazine-1-carbodithioate **(5j)**

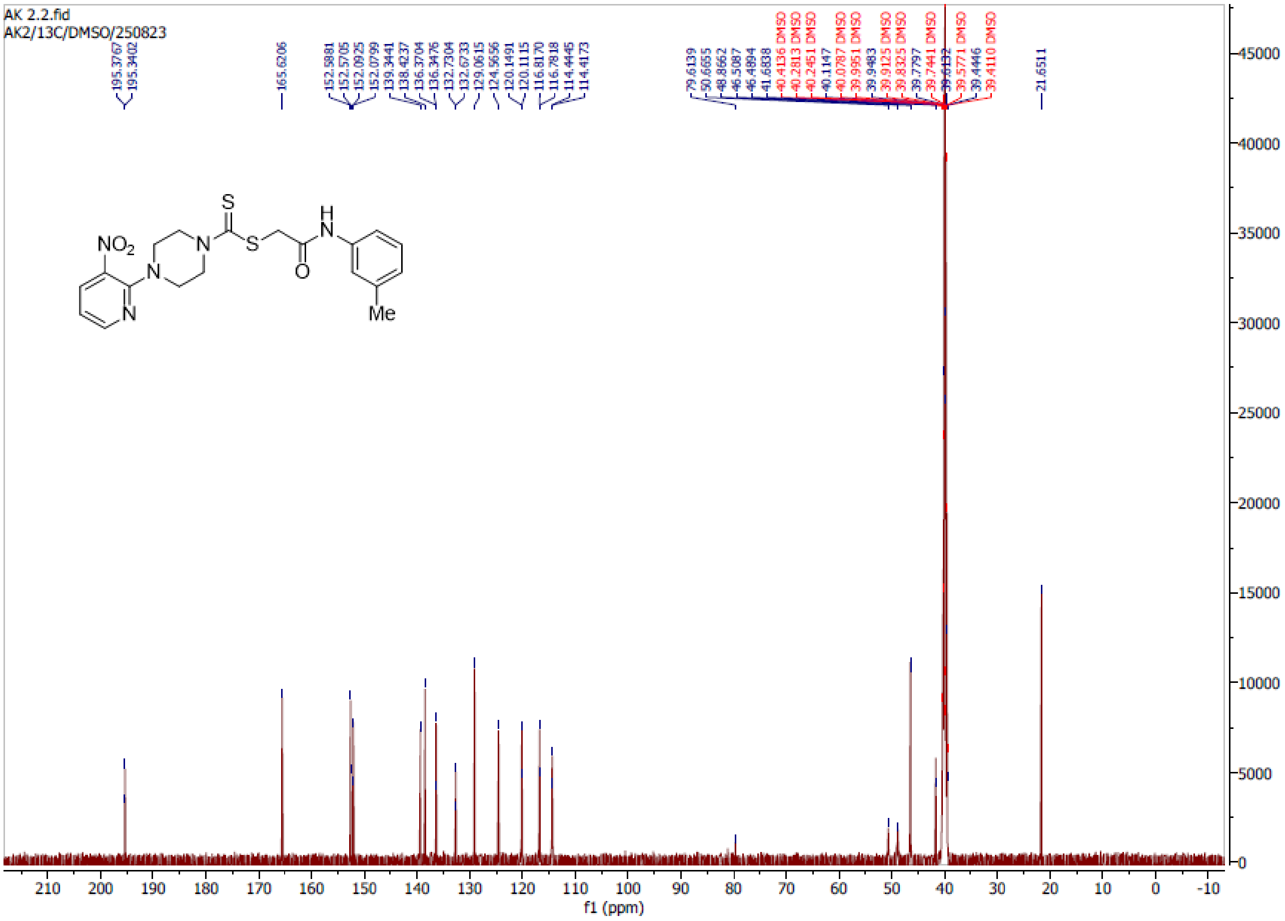

Figure S15: ^{13}CNMR of 2-oxo-2-(m-tolylamino)ethyl 4-(3-nitropyridin-2-yl)piperazine-1-carbodithioate **(5k)**

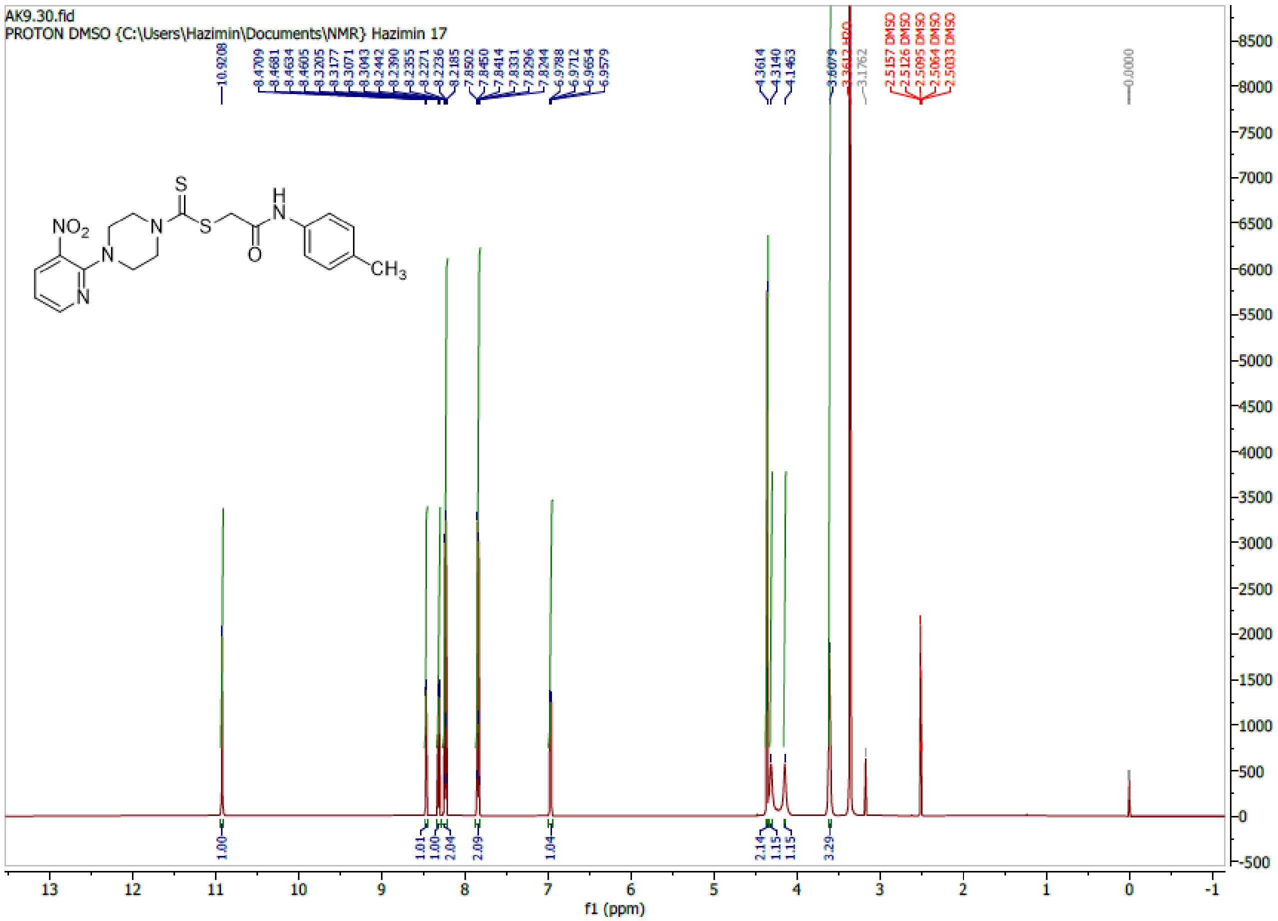

Figure S16: ^{1}HNMR of 2-oxo-2-(p-tolylamino)ethyl 4-(3-nitropyridin-2-yl)piperazine-1-carbodithioate **(5l)**

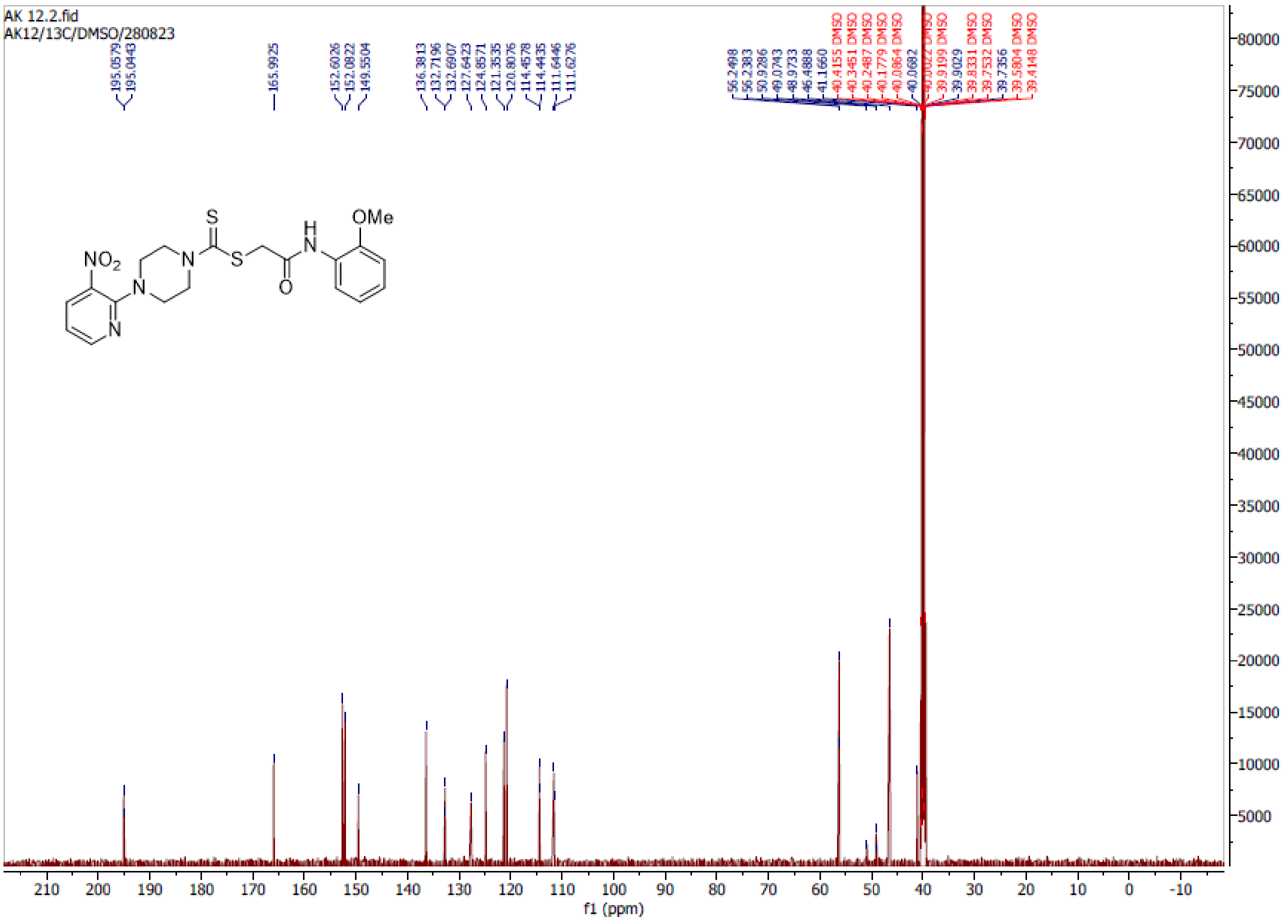

Figure S17: ^{13}CNMR of 2-((2-methoxyphenyl)amino)-2-oxoethyl 4-(3-nitropyridin-2-yl)piperazine-1-carbodithioate **(5m)**

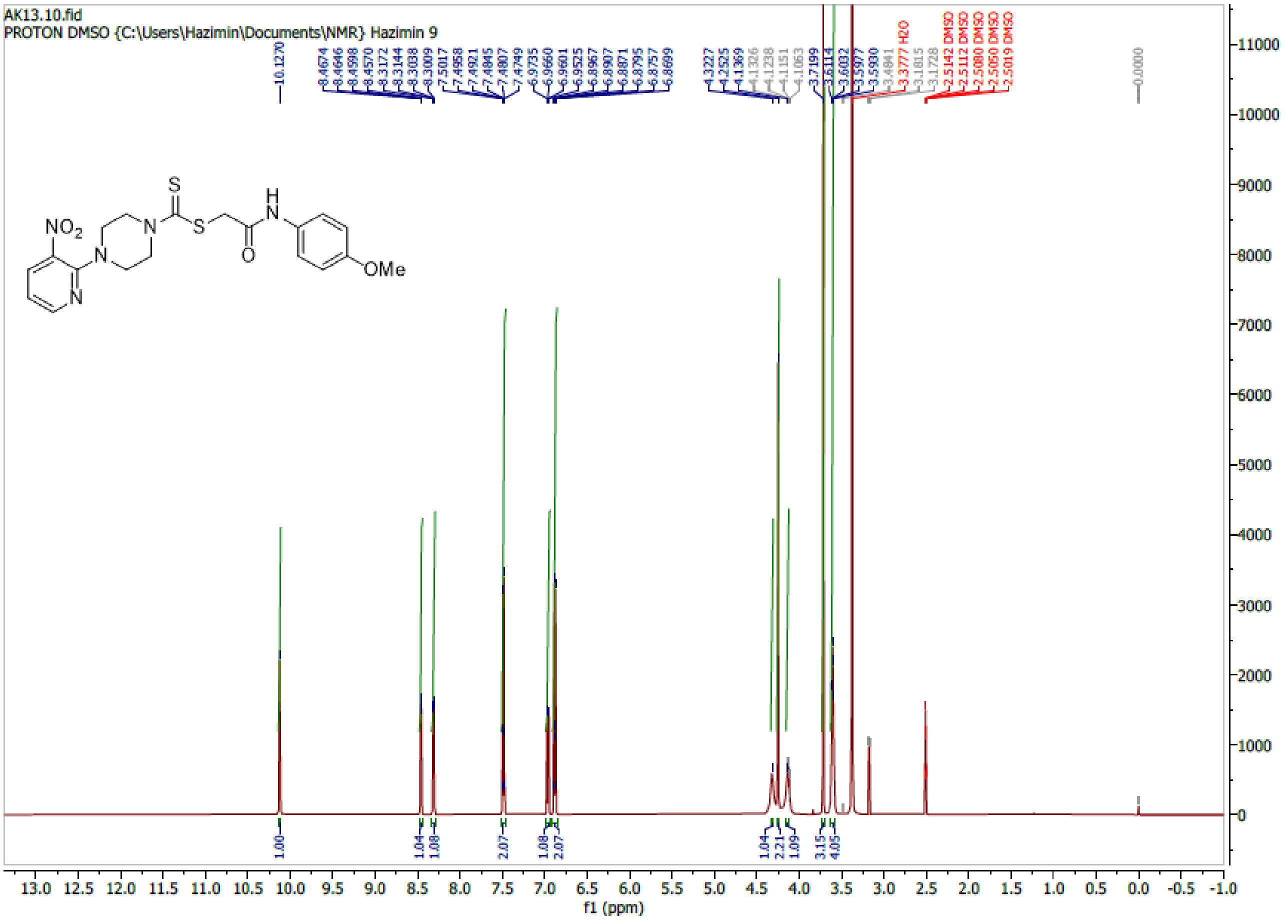

Figure S18: ^{1}HNMR of 2-((4-methoxyphenyl)amino)-2-oxoethyl 4-(3-nitropyridin-2-yl)piperazine-1-carbodithioate **(5n)**

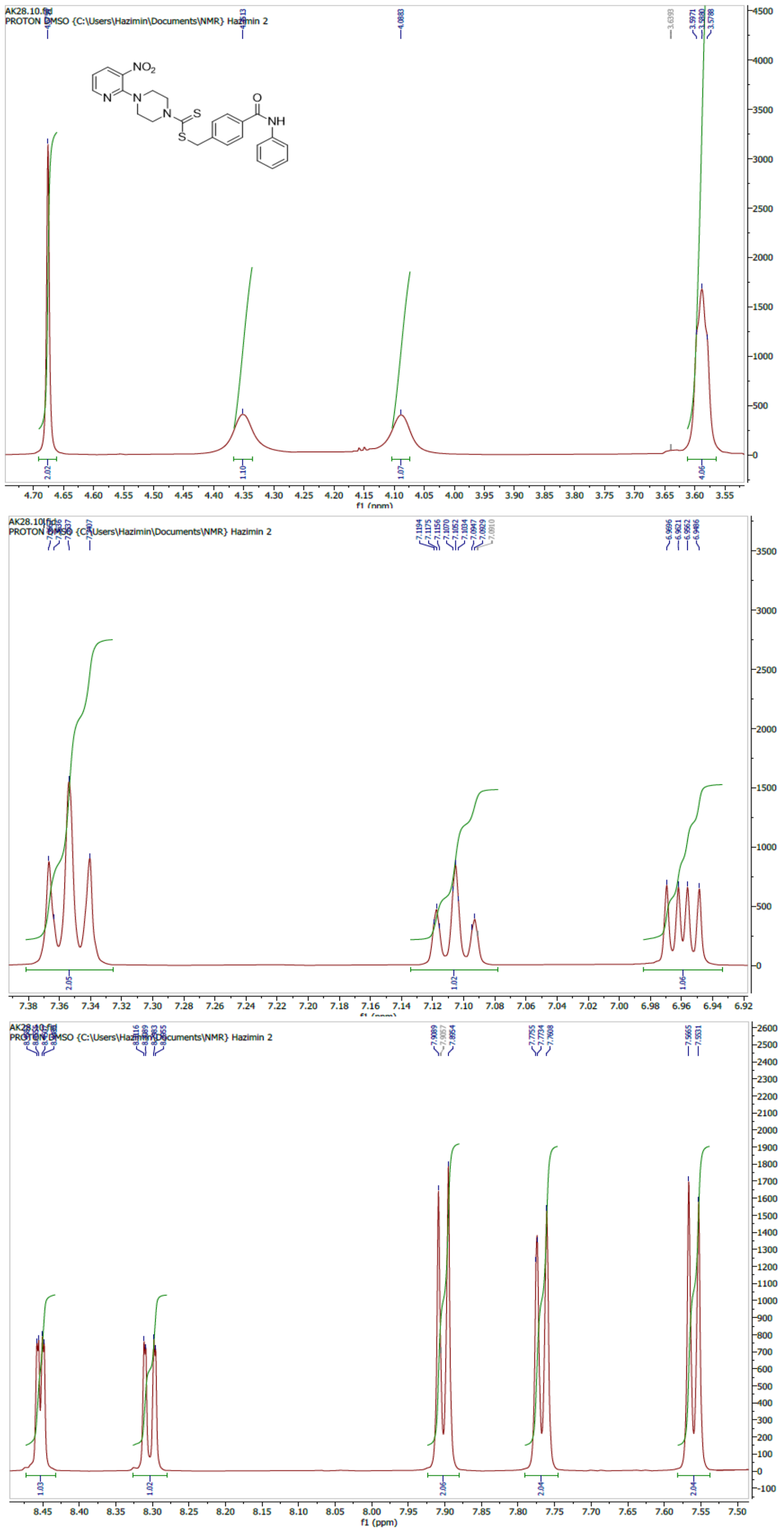

Figure S19: ^{1}HNMR of 4-(phenylcarbamoyl)benzyl 4-(3-nitropyridin-2-yl)piperazine-1-carbodithioate **(7a)**

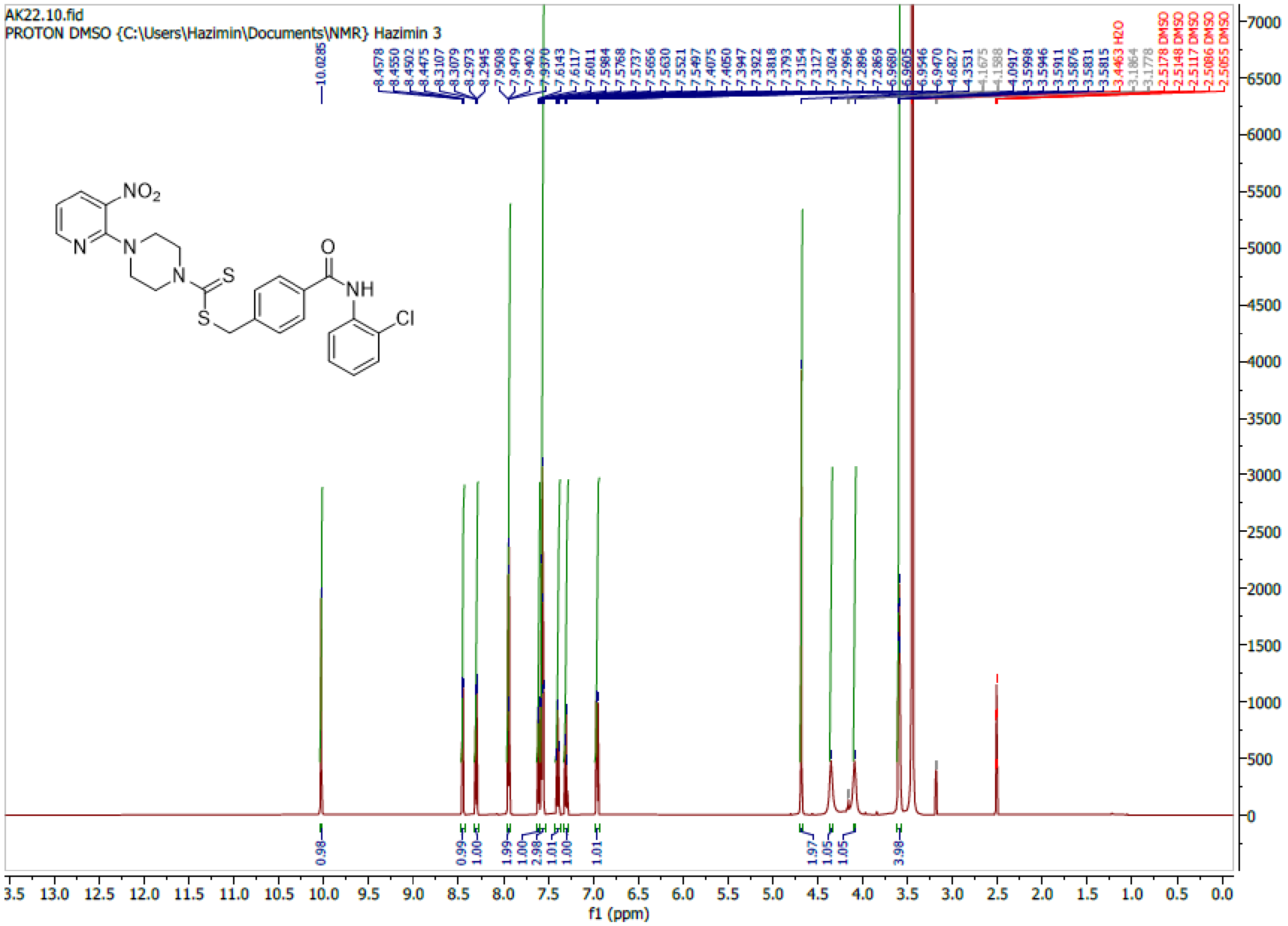

Figure S20: ^{1}HNMR of 4-((2-chlorophenyl)carbamoyl)benzyl 4-(3-nitropyridin-2-yl)piperazine-1-carbodithioate **(7b)**

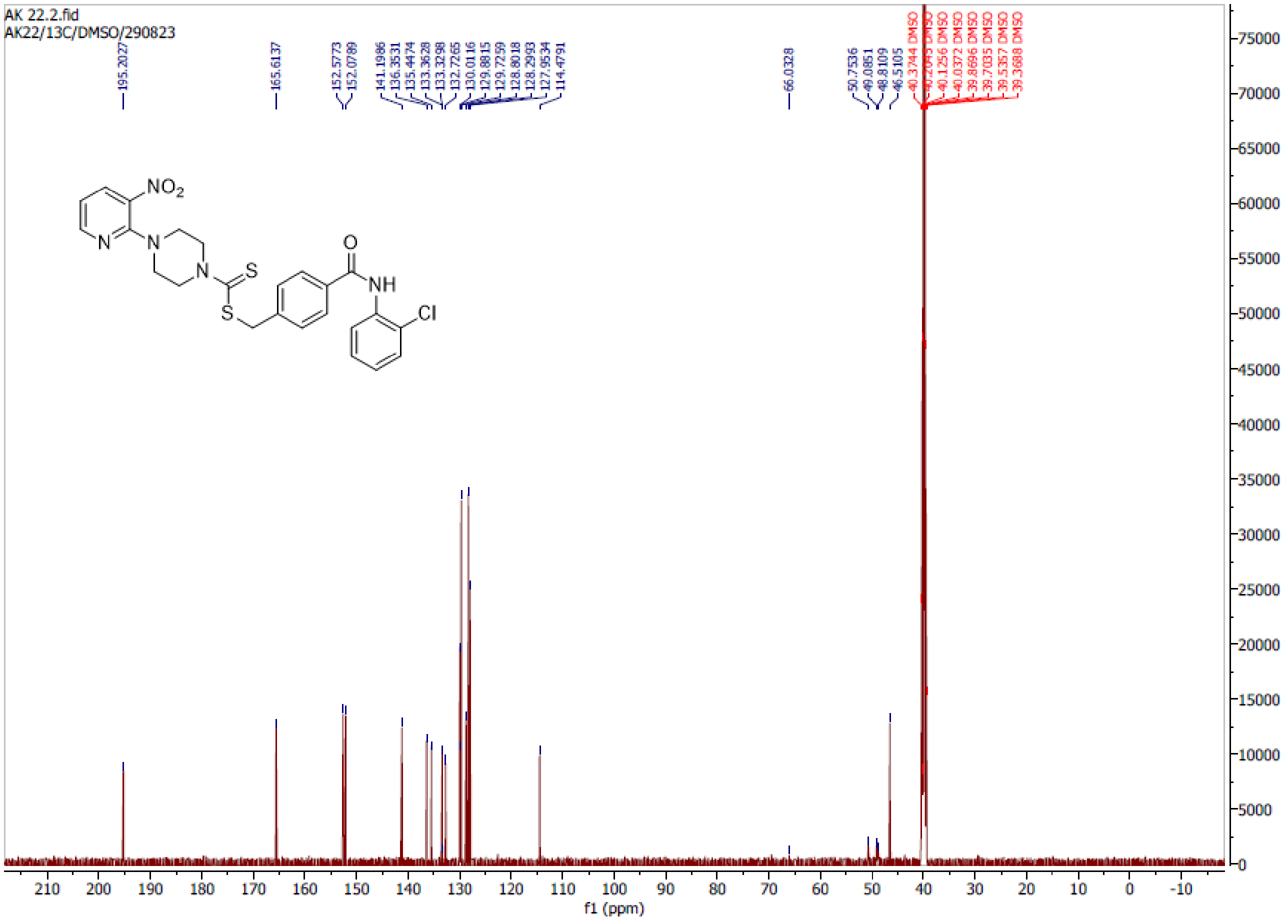

Figure S21: ^{13}CNMR of 4-((2-chlorophenyl)carbamoyl)benzyl 4-(3-nitropyridin-2-yl)piperazine-1-carbodithioate **(7b)**

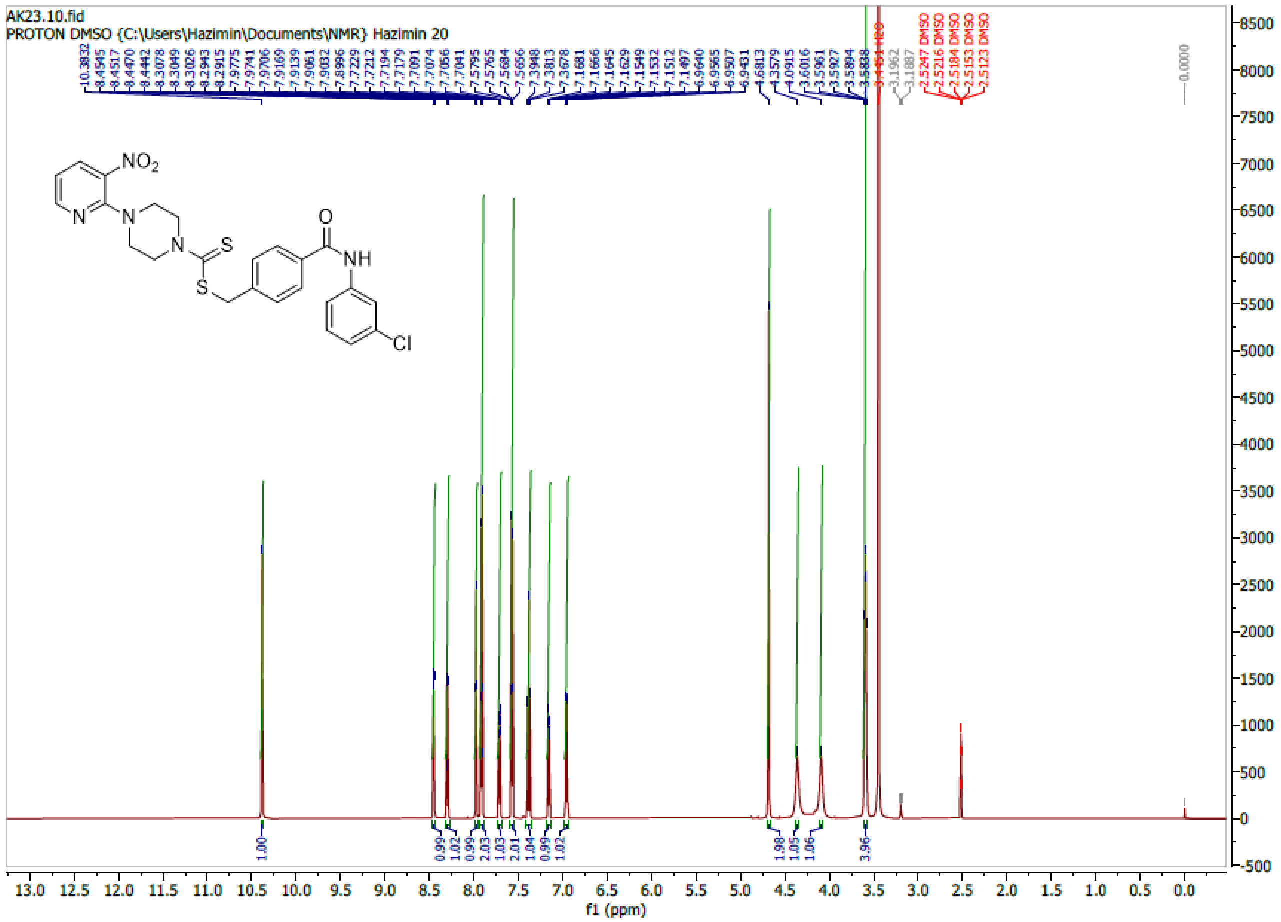

Figure S22: ^{1}HNMR of 4-((3-chlorophenyl)carbamoyl)benzyl 4-(3-nitropyridin-2-yl)piperazine-1-carbodithioate **(7c)**

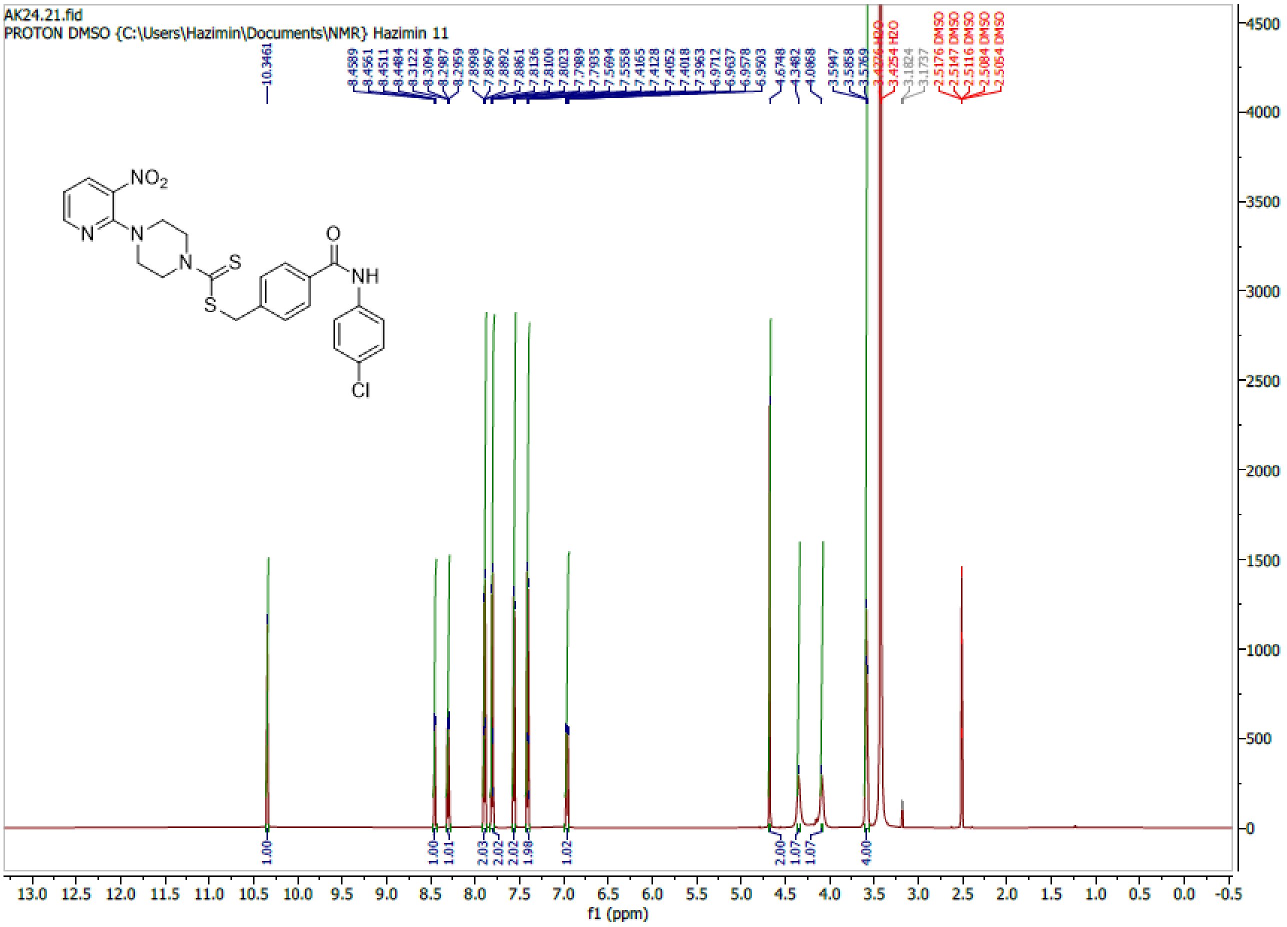

Figure S23: ^{1}HNMR of 4-((4-chlorophenyl)carbamoyl)benzyl 4-(3-nitropyridin-2-yl)piperazine-1-carbodithioate **(7d)**

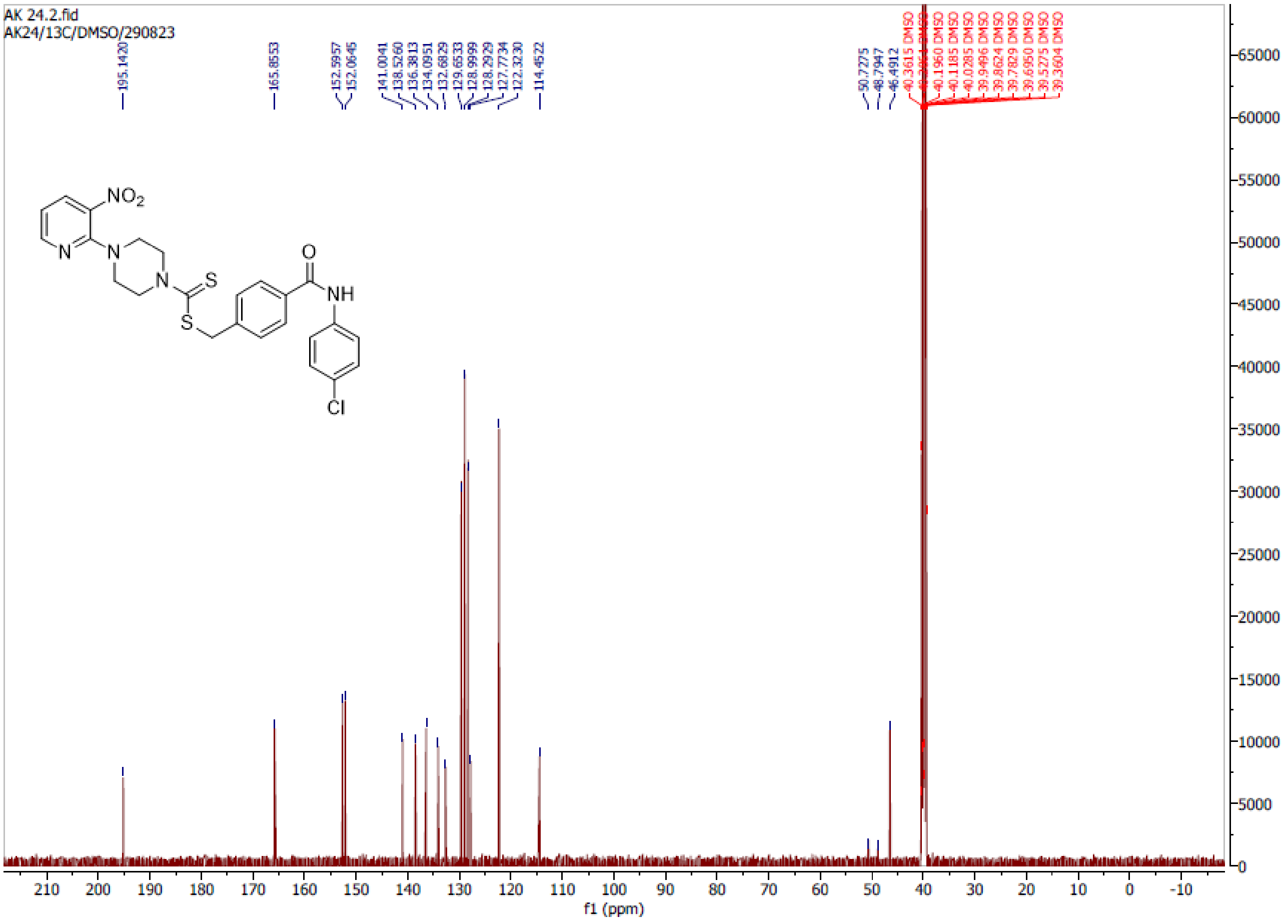

Figure S24: ^{13}CNMR of 4-((4-chlorophenyl)carbamoyl)benzyl 4-(3-nitropyridin-2-yl)piperazine-1-carbodithioate **(7d)**

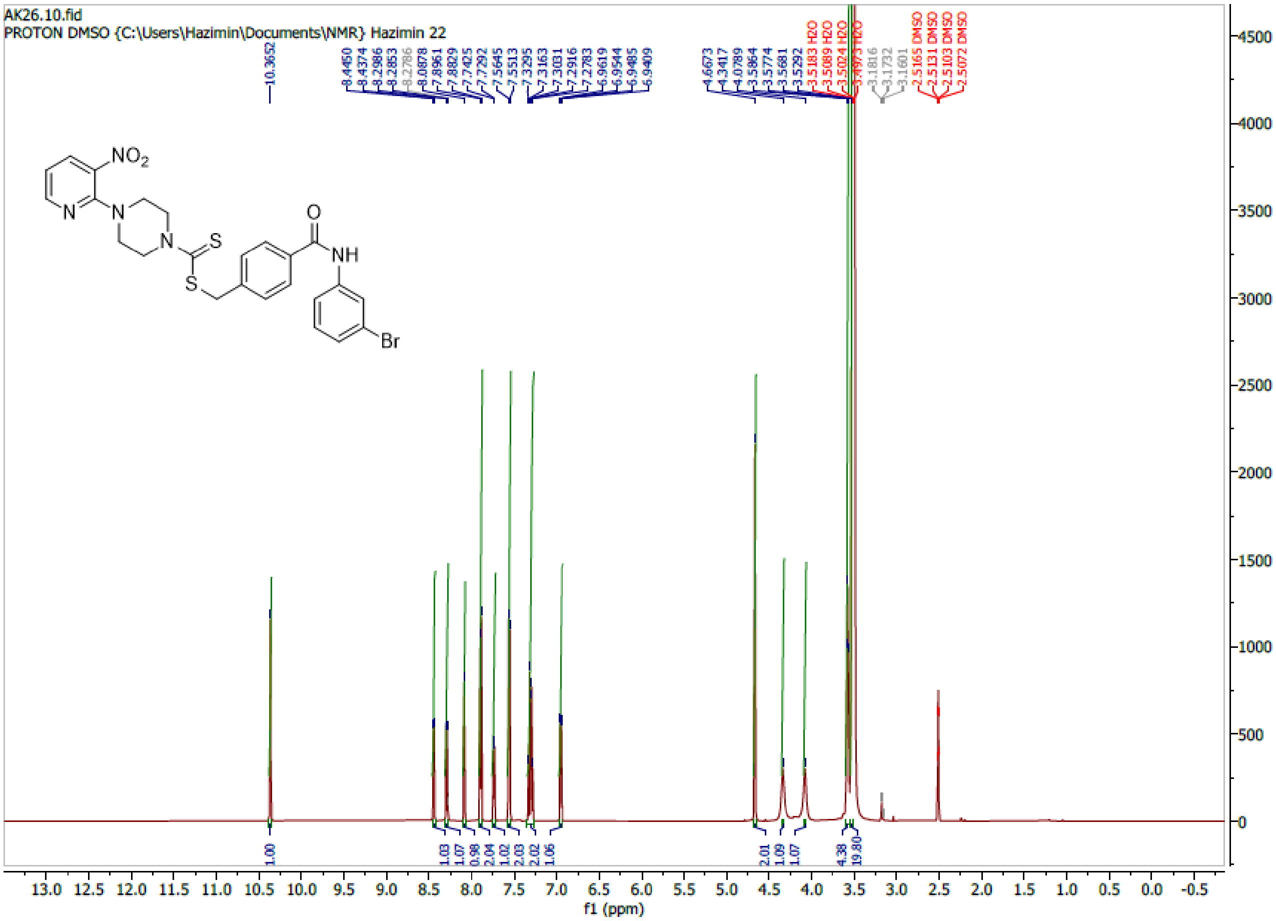

Figure S25: ^{1}HNMR of 4-((3-bromophenyl)carbamoyl)benzyl 4-(3-nitropyridin-2-yl)piperazine-1-carbodithioate **(7e)**

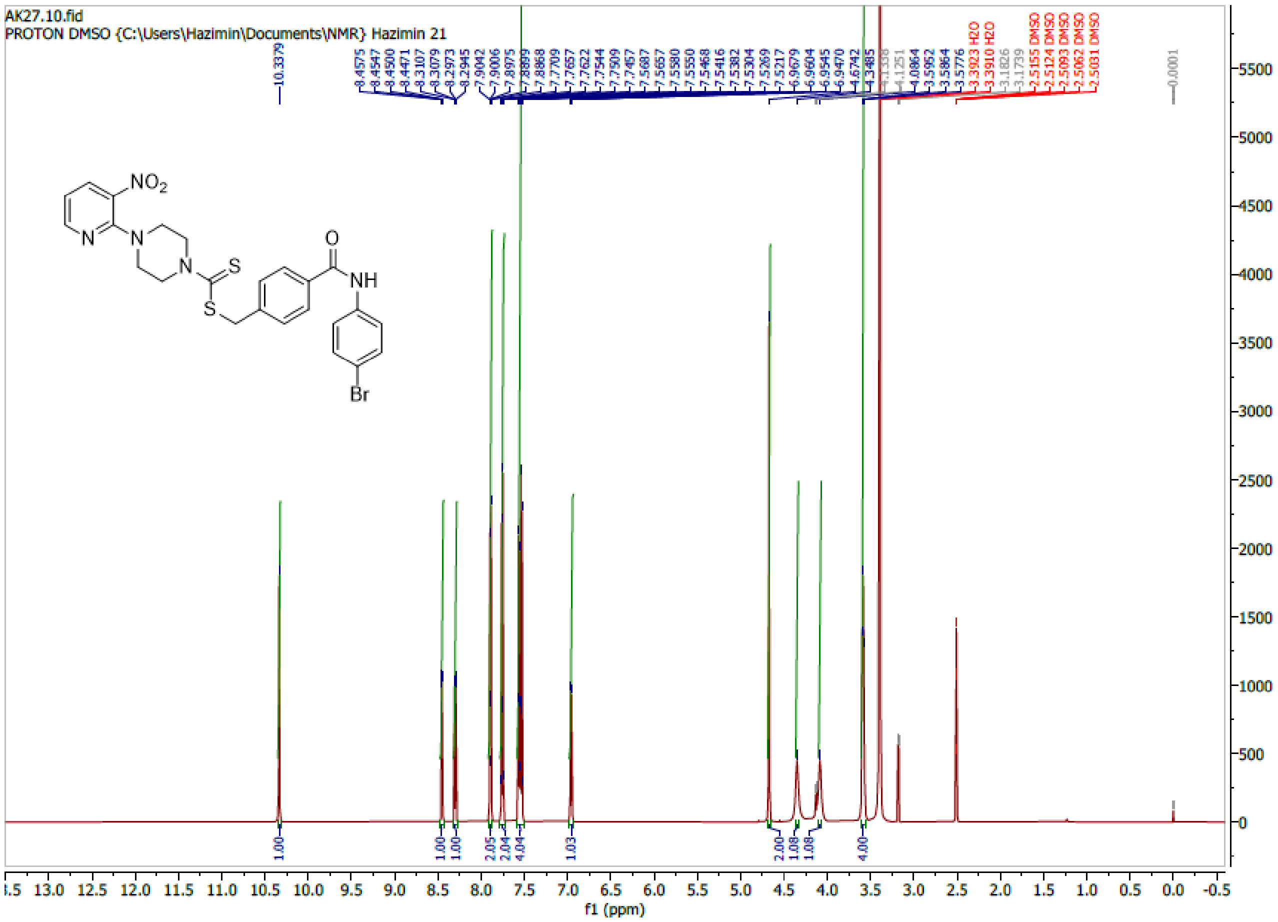

Figure S26: ^{1}HNMR of 4-((4-bromophenyl)carbamoyl)benzyl 4-(3-nitropyridin-2-yl)piperazine-1-carbodithioate **(7f)**

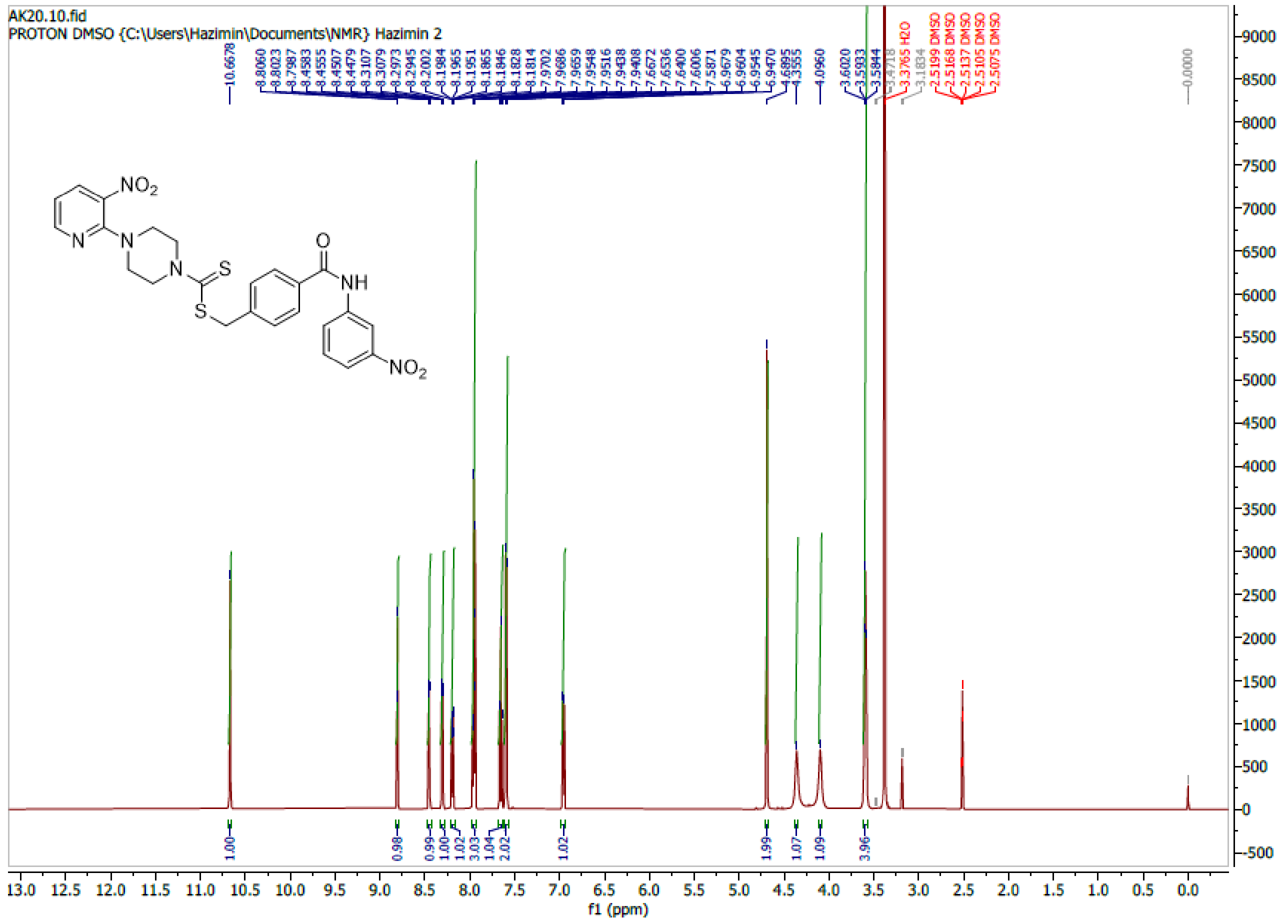

Figure S27: ^{1}HNMR of 4-((3-nitrophenyl)carbamoyl)benzyl 4-(3-nitropyridin-2-yl)piperazine-1-carbodithioate **(7h)**

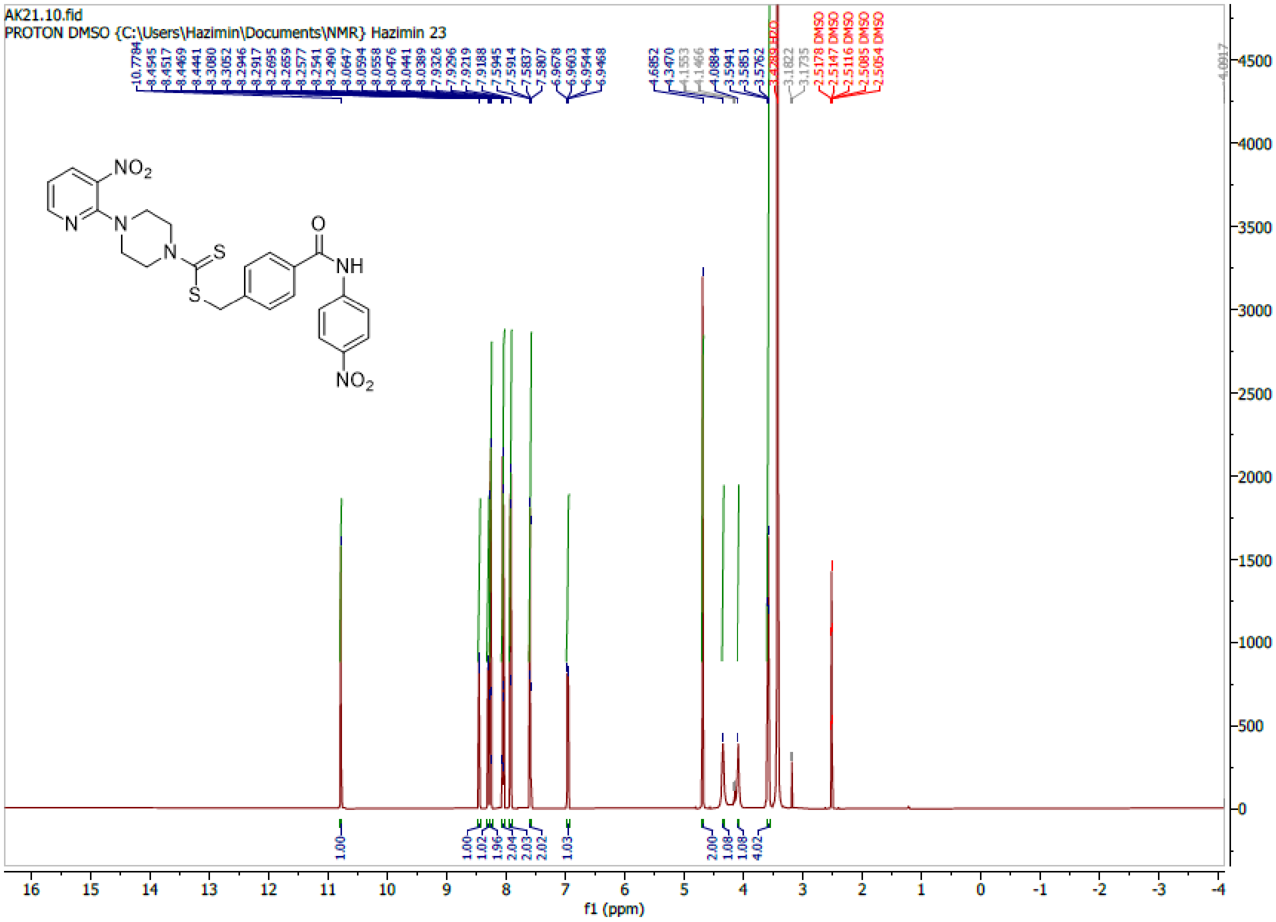
AK21.10.fid
PROTON DMSO {C:\Users\Hazimin\Documents\NMR} Hazimin 23
NO2
N
N
N
S
S
O
NH
NO2
f1 (ppm)

Figure S28: ^{1}HNMR of 4-((4-nitrophenyl)carbamoyl)benzyl 4-(3-nitropyridin-2-yl)piperazine-1-carbodithioate **(7i)**

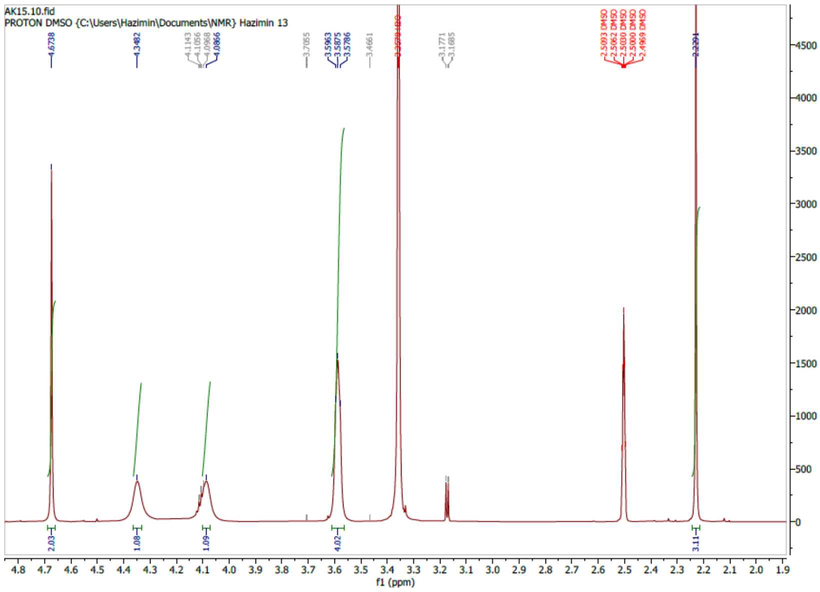
AK15.10.fid
PROTON DMSO {C:\Users\Hazimin\Documents\NMR} Hazimin 13
f1 (ppm)

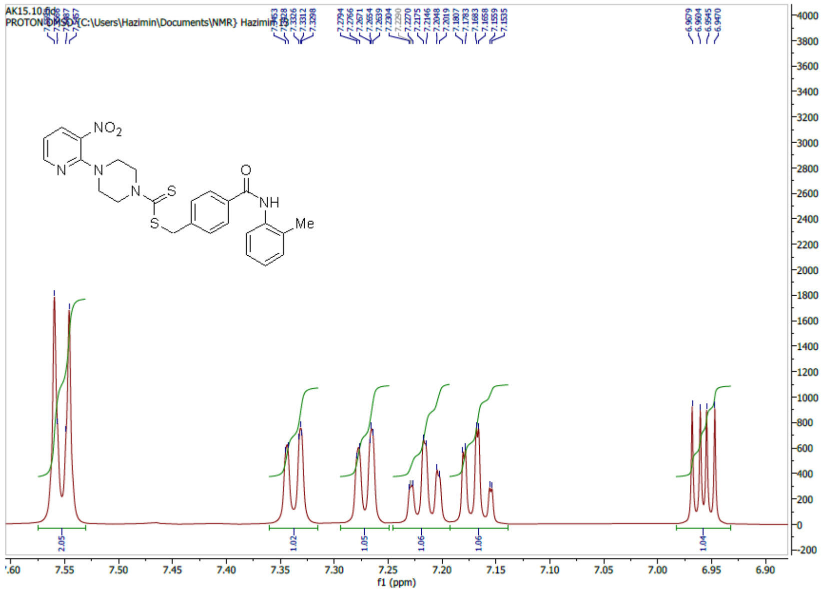
AK15.10.fid
PROTON DMSO {C:\Users\Hazimin\Documents\NMR} Hazimin 13
NO2
N
O
S
NH
Me
f1 (ppm)

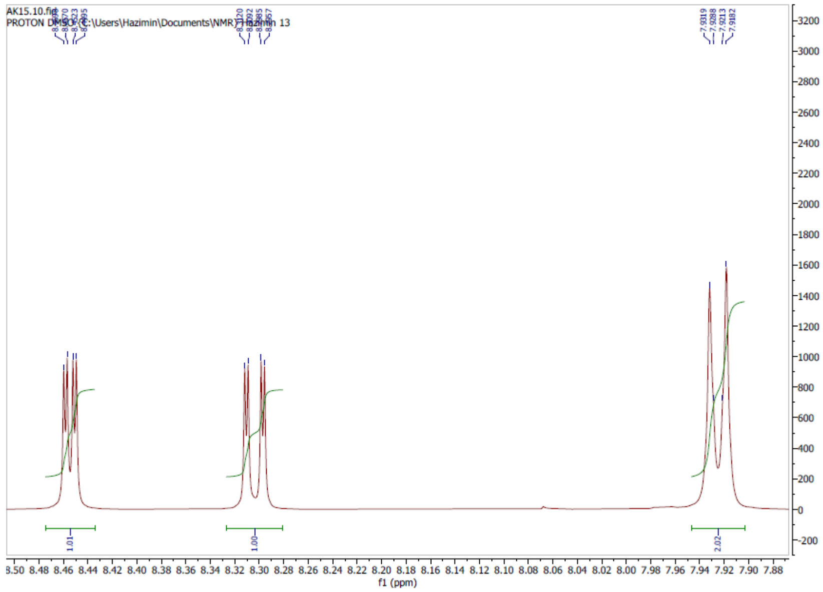
AK15.10.fid
PROTON DMSO {C:\Users\Hazimin\Documents\NMR} Hazimin 13
f1 (ppm)

Figure S29: ^{1}HNMR of 4-(o-tolylcarbamoyl)benzyl 4-(3-nitropyridin-2-yl)piperazine-1-carbodithioate **(7j)**

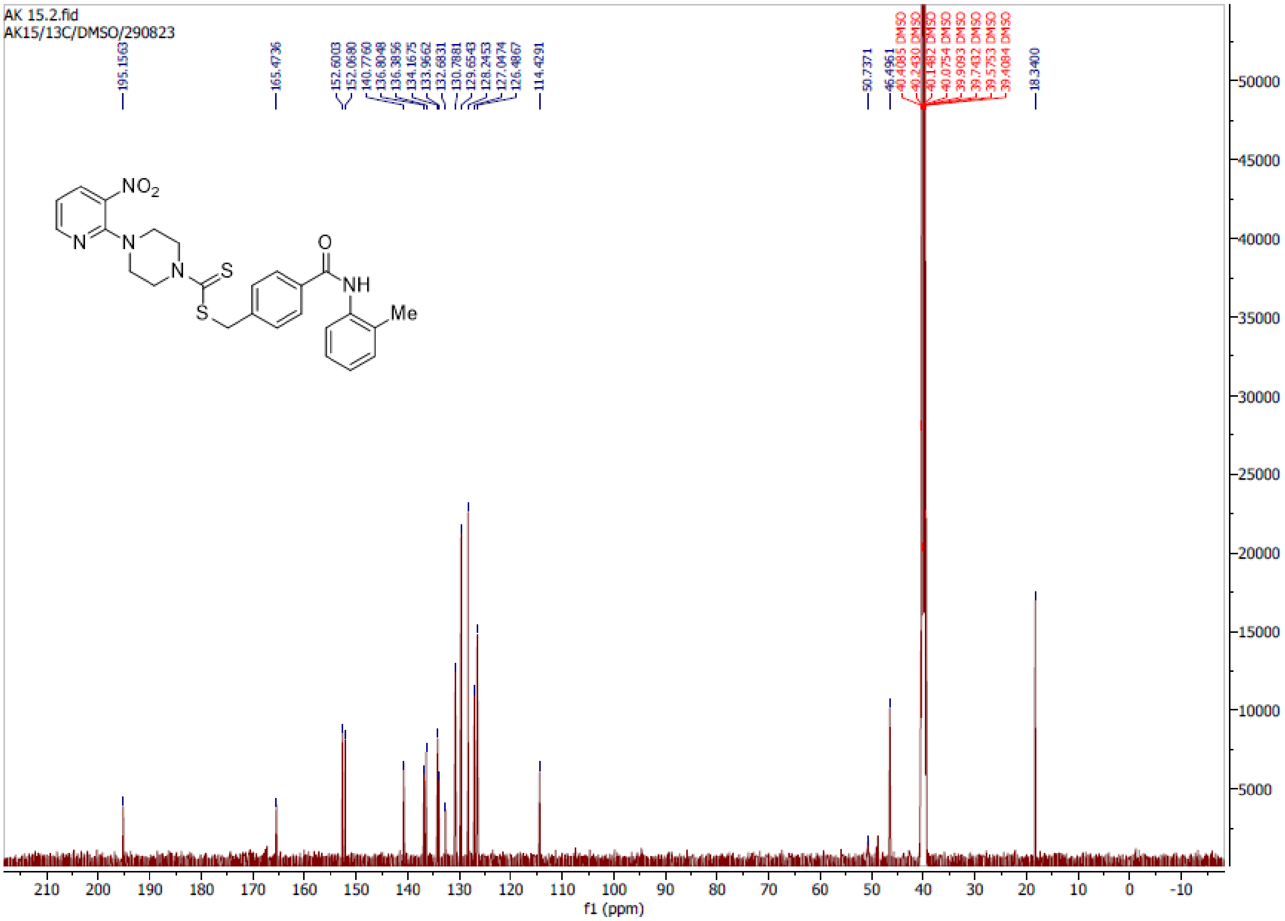

Figure S30: ^{13}CNMR of 4-(o-tolylcarbamoyl)benzyl 4-(3-nitropyridin-2-yl)piperazine-1-carbodithioate **(7j)**

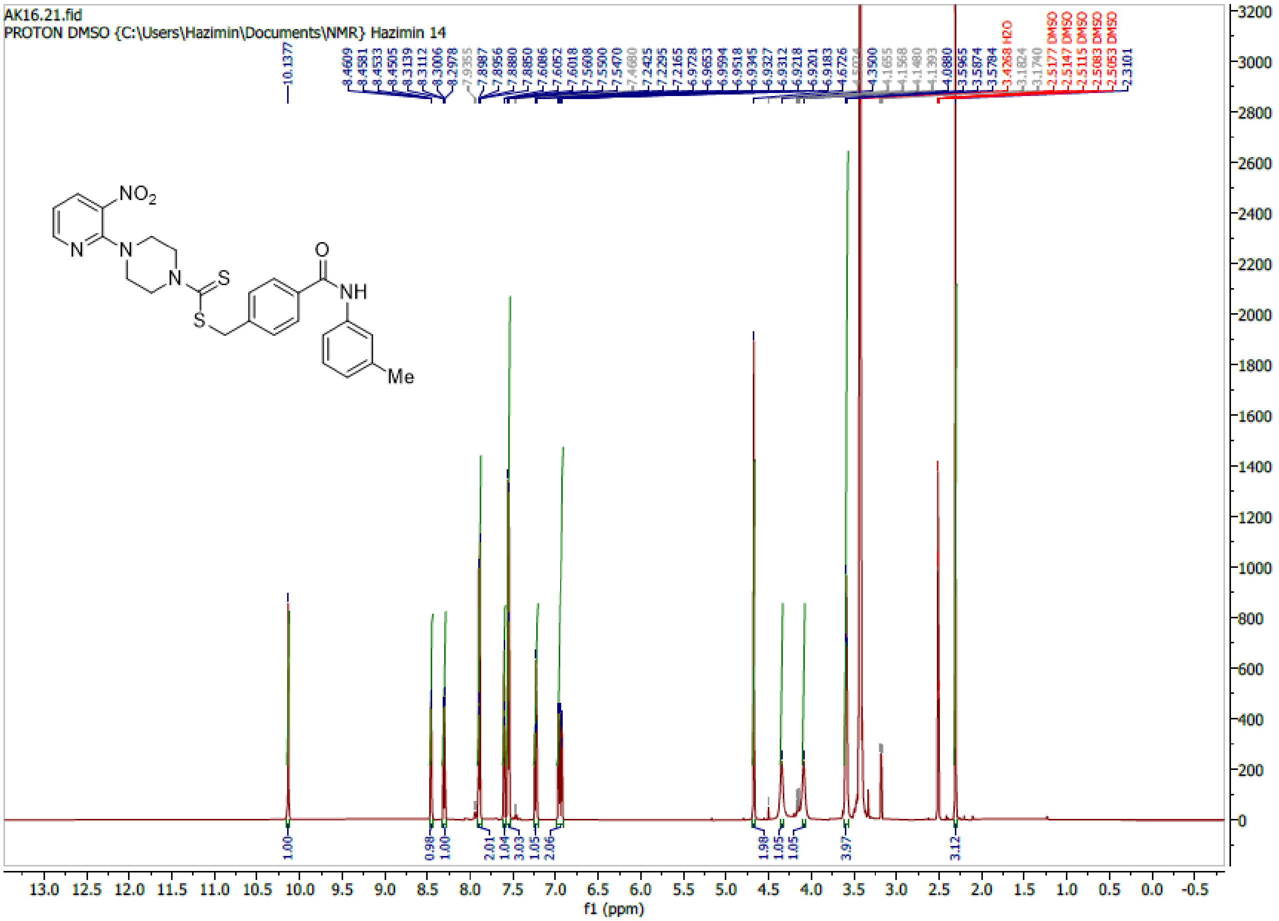

Figure S31: ^{1}HNMR of 4-(m-tolylcarbamoyl)benzyl 4-(3-nitropyridin-2-yl)piperazine-1-carbodithioate **(7k)**

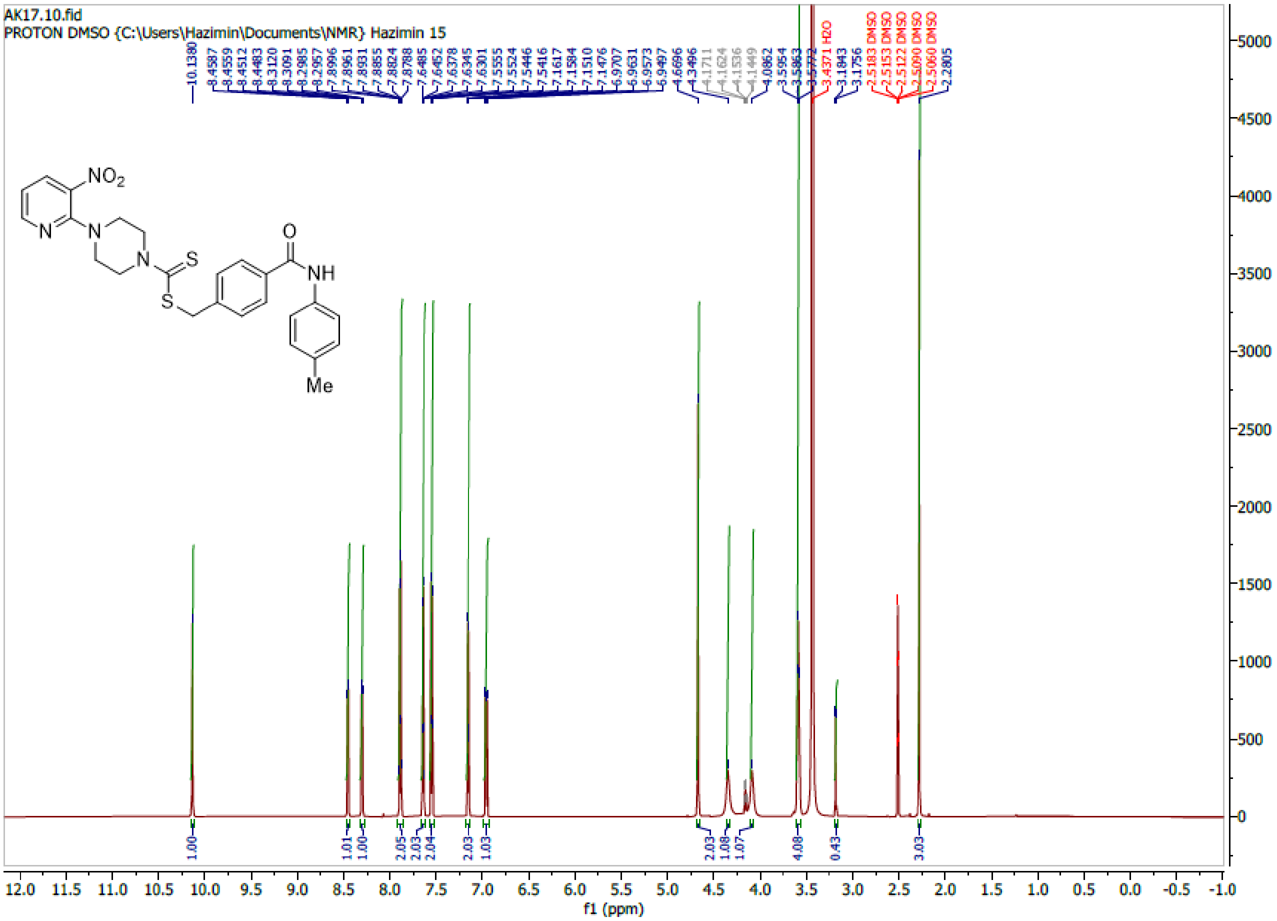

Figure S32: ^{1}HNMR of 4-(p-tolylcarbamoyl)benzyl 4-(3-nitropyridin-2-yl)piperazine-1-carbodithioate **(7l)**

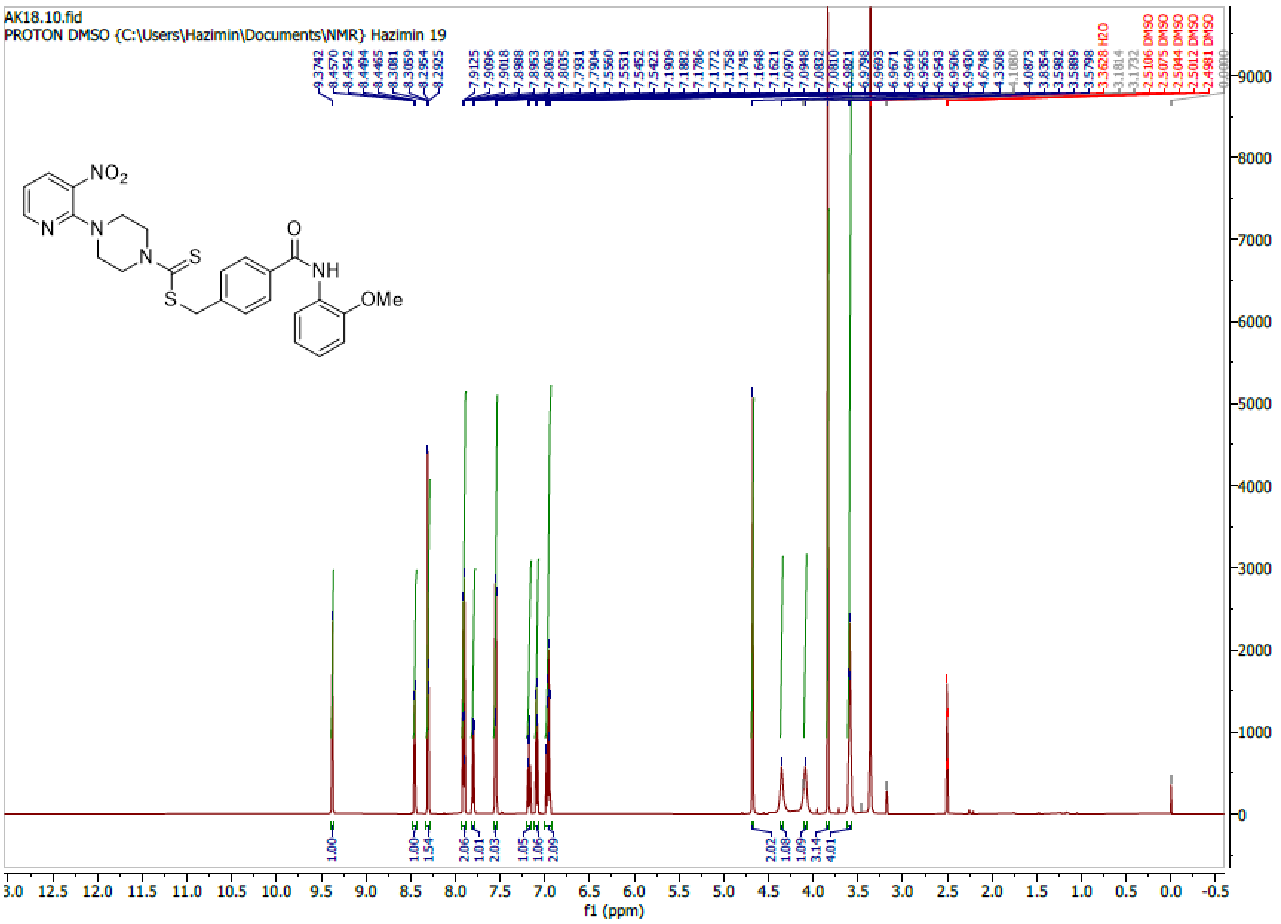

Figure S33: ^{1}HNMR of 4-((2-methoxyphenyl)carbamoyl)benzyl 4-(3-nitropyridin-2-yl)piperazine-1-carbodithioate **(7m)**

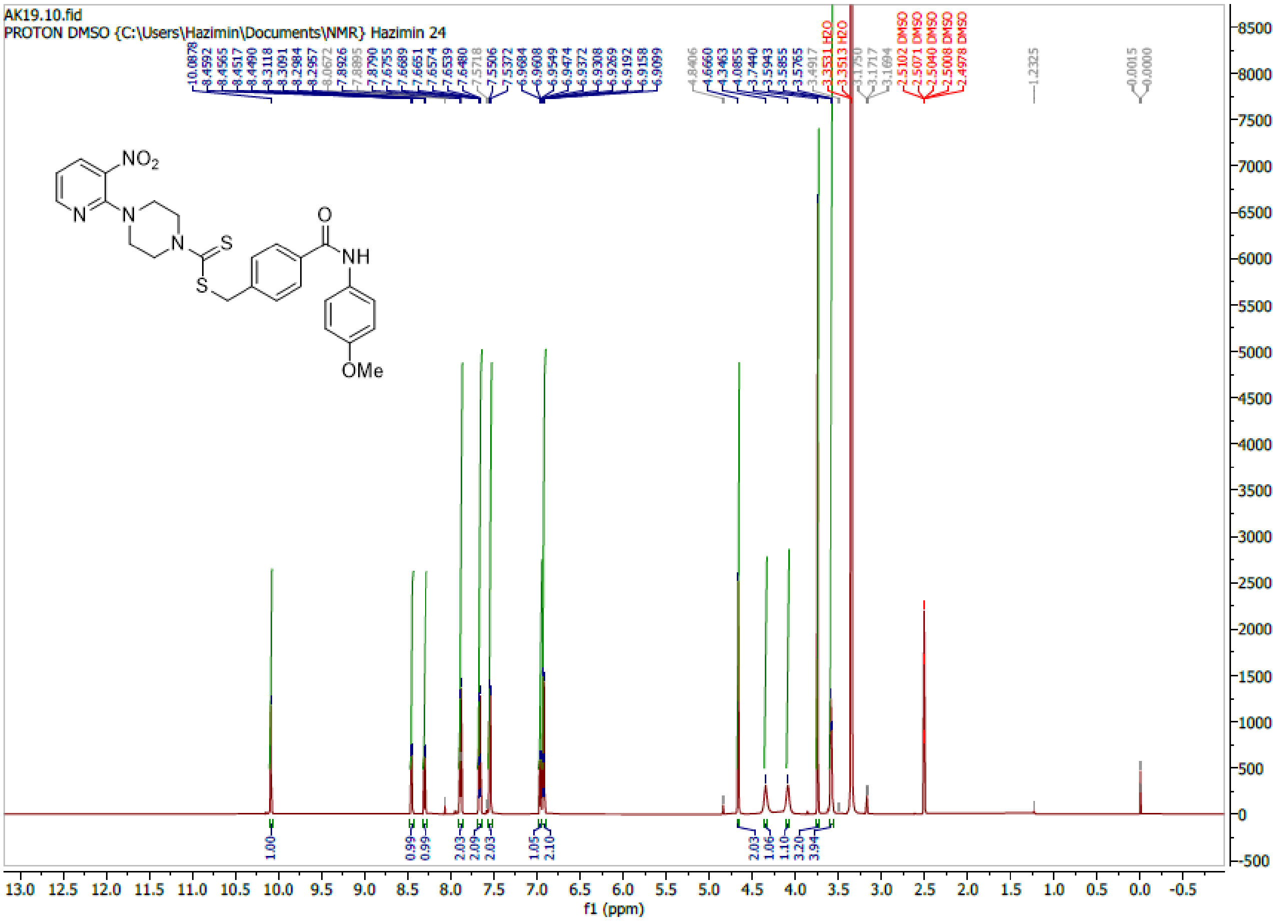
AK19.10.fid
PROTON DMSO {C:\Users\Hazimin\Documents\NMR} Hazimin 24
NO2
N
N
N
S
S
O
NH
OMe
f1 (ppm)

Figure S34: ^{1}HNMR of 4-((4-methoxyphenyl)carbamoyl)benzyl 4-(3-nitropyridin-2-yl)piperazine-1-carbodithioate **(7n)**

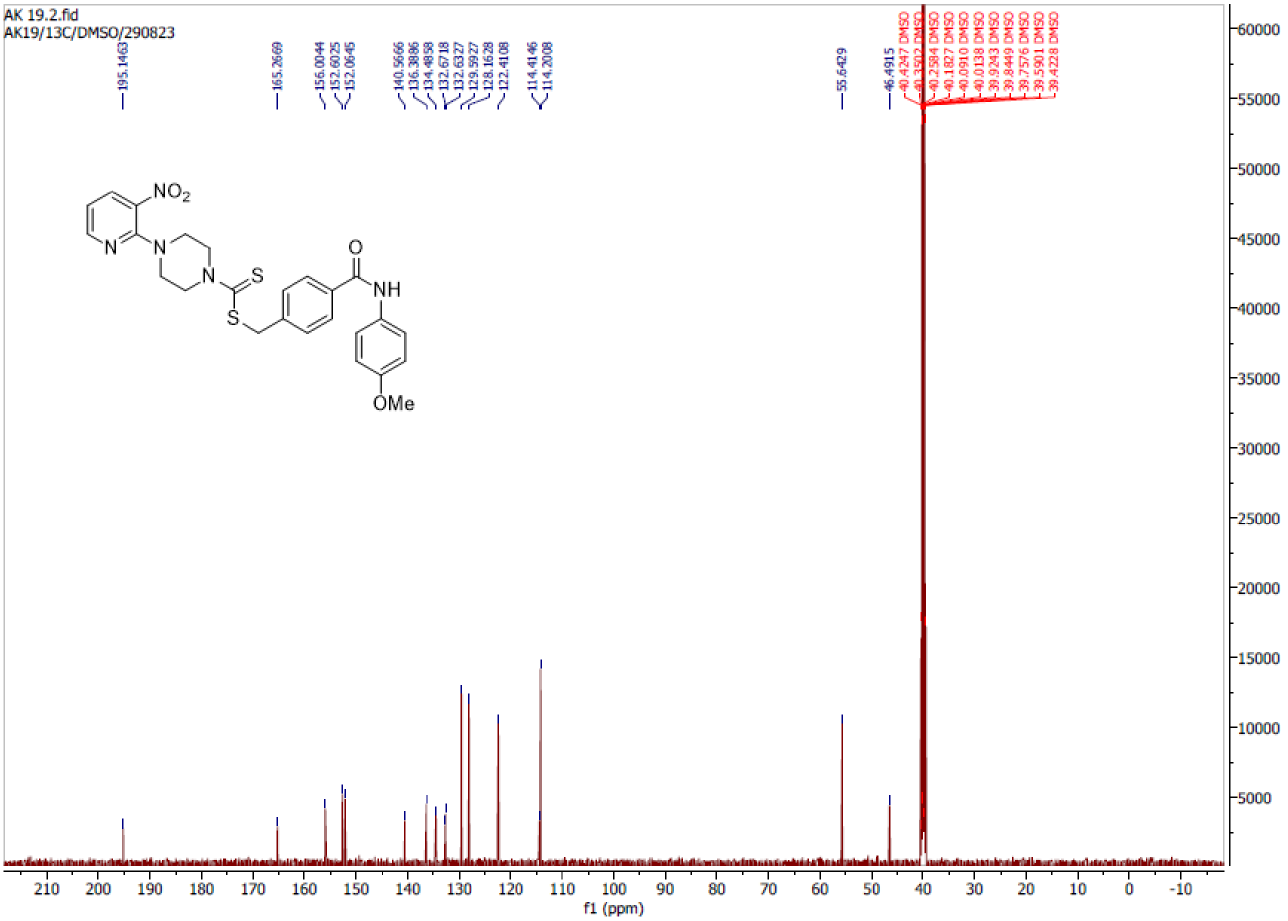

Figure S35: ^{13}CNMR of 4-((4-methoxyphenyl)carbamoyl)benzyl 4-(3-nitropyridin-2-yl)piperazine-1-carbodithioate **(7n)**
